# Correlation between Genes of the ceRNA Network and Tumor-Infiltrating Immune Cells and Their Biomarker Screening in Kidney Renal Clear Cell Carcinoma

**DOI:** 10.1155/2022/4084461

**Published:** 2022-08-29

**Authors:** Aoran Kong, Hui Dong, Guangwen Zhang, Shuang Qiu, Mengyuan Shen, Xiaohan Niu, Lixin Wang

**Affiliations:** ^1^Center of Laboratory Medicine, General Hospital of Ningxia Medical University, Yinchuan 750004, China; ^2^Clinical Laboratory, XiangYang Hospital of Traditional Chinese Medicine, Xiangyang, Hubei 441000, China; ^3^Institute of Medical Sciences, General Hospital of Ningxia Medical University, Yinchuan 750004, China; ^4^Department of Radiology, Xijing Hospital, Fourth Military Medical University, Xi'an, Shaanxi, China; ^5^Clinical Laboratory, Yichang Central Hospital, Yichang, Hubei 443000, China; ^6^Clinical Laboratory, ShangHai Changzheng Hospital, Shanghai 200003, China; ^7^Department of Medical Examination, The People's Hospital of West Coast, Qingdao, Shangdong 2664000, China

## Abstract

This study aimed to using bioinformatics tools, qPCR, and the immunohistochemical analysis to find out factors related to the early diagnosis and prognosis of kidney renal clear cell carcinoma (KIRC). The expression profiles of lncRNA, miRNA, and mRNA of KIRC were downloaded from The Cancer Genome Atlas database. A ceRNA regulatory network was constructed based on the interaction between these three differentially expressed genes. The CIBERSORT deconvolution algorithm was used to analyze the differential distribution of 22 types of immune cells. The Kaplan–Meier survival and Cox analyses were used to screen genes of the ceRNA network and also immune cell subtypes related to the clinical and prognostic prediction of KIRC. Co-expression regulatory relationships were found among LINC01426, LINC00894, CCNA2, L1 cell adhesion molecule (L1CAM), and T follicular helper cells, which served as potential biomarkers. The results of quantitative reverse transcriptase-polymerase chain reaction showed that LINC01426 was upregulated while L1CAM was downregulated in KIRC, but no difference was found in the expression levels of LINC00894 and CCNA2 in cancer and adjacent samples. The immunohistochemical analysis showed that T follicular helper cells were more concentrated in core tissues and metastases of KIRC. In a word, co-expression relationships were found among LINC01426, L1CAM, and T follicular helper cells, and they may serve as biomarkers for early diagnosis and prognostic evaluation of KIRC.

## 1. Introduction

Kidney renal clear cell carcinoma (KIRC) originates from proximal tubular epithelial cells [1]. It is the most common and aggressive subtype of renal cancer, accounting for approximately 75%–80% [2]. Most patients are diagnosed in the advanced stage because the initial clinical symptoms and signs of KIRC are relatively hidden [3]. Compared with other subtypes of kidney cancer, KIRC has a higher recurrence rate and metastasis rate. Although surgical treatment, molecular-targeted therapy (sorafenib and sunitinib), immunotherapy (interleukin-2), and other treatments developed in recent years have greatly improved the survival time of patients; the 5-year survival rate is still less than 10% [4, 5]. Therefore, a biomarker that can detect KIRC early and predict its prognosis needs to be identified.

Salmena et al. formulated a hypothesis about ceRNA in 2011; they believed that long noncoding RNAs (lncRNAs) use some core seed sequences to adsorb the corresponding miRNA, thereby interfering with the abundance of target gene mRNA and affecting gene expression [6]. A large number of studies have shown that ceRNA played a vital role in the occurrence, development, and prognosis of tumors [7]. For example, Wang et al. experimentally proved that lncRNA UCA1 was used as the ceRNA of miR-182-5p to positively regulate the expression of Delta-like4 (DLL4), thereby promoting the malignant phenotype of renal cancer cells and playing a carcinogenic role in the pathogenesis of renal cancer [8]. Human immune surveillance is an important immune function of the body to prevent tumors, and evading the destruction of the body's immune function is one of the important mechanisms of tumors [9, 10]. In recent years, the distribution and density of local immune cells have received wide attention from scholars in tumor diagnosis and prognostic evaluation [11]. Studies have shown differences in infiltrating immune cells in different types of sarcoma [12]. Liang et al. found that Janus Kinase 3 (JAK3) moderately to strongly positively correlated with the abundance of B cells, CD8+ T cells, CD4+ T cells, neutrophils, and dendritic cells in KIRC, which may become potential biomarkers of KIRC [13]. Although a large number of studies have explored the correlation between infiltrating immune cells and tumor occurrence, development, prognosis, and so on, the specific mechanism of action in tumors has not yet been clearly elucidated.

**Figure 1:**
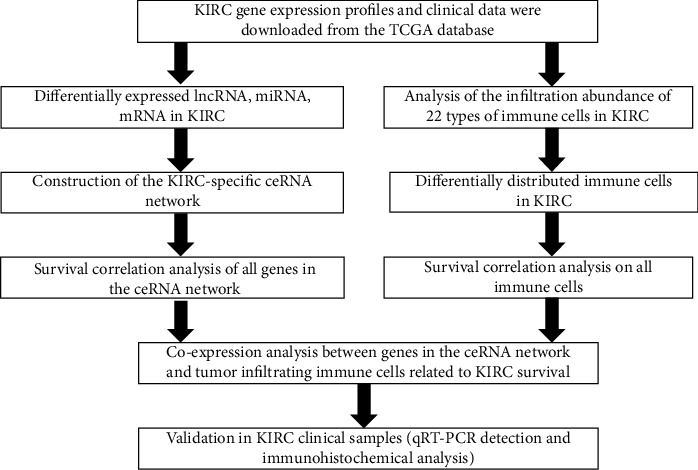
A flowchart depicting the analytical process.

This topic analyzed the potential roles of the ceRNA network and tumor-infiltrating immune cells in KIRC in tumorigenesis, metastasis, and prognosis. A flowchart explaining this process is given in Figure 1. In conclusion, this study might offer new ideas for prognostic monitoring of patients with KIRC and research on new treatment methods.

## 2. Materials and Methods

### 2.1. Data Acquisition and Differential Expression Analysis of Genes

Metadata files, manifest files, and cart files of KIRC transcriptome and miRNA and patient clinical information were downloaded from The Cancer Genome Atlas (TCGA) database. After decompressing the cart file, the Perl script was run to obtain the original transcriptome and miRNA matrix files. The gene names were converted using the human. gtf file downloaded from the Ensembl database and the mature. fa file downloaded from the miRBase database. DESeq2 package in R4.0.2 software was used for differential expression analysis to obtain differentially expressed lncRNAs, miRNAs, and mRNA ((false discovery rate, FDR) < 0.05, |log (fold change)| > 2).

### 2.2. Construction of the ceRNA Network

LncRNA–miRNA and miRNA–mRNA interactions predicted from the miRcode [14] and StarBase [15] databases, respectively, showing significant results in hypergeometric testing and correlation analysis, were selected for the visualization of the ceRNA network using the Cytoscape 3.7.2 software.

### 2.3. Clinical Significance of the ceRNA Network in KIRC

Single-factor Cox regression, lasso regression, and multifactor Cox regression analyses were performed on all genes in the ceRNA network, and a risk scoring model was built for the selected genes. The diagnostic value of the model was assessed through the risk survival curve and receiver operating characteristic (ROC) curve. The Kaplan–Meier survival method was employed to perform the survival analysis of all genes in the network in batches.

### 2.4. Abundance Analysis and Differential Expression Analysis of Infiltrating Immune Cells

The gene expression feature set of 22 types of immune cell subtypes was downloaded from the CIBERSORT website. Based on the gene expression profile, the e1071 package was run to obtain the abundance of infiltrating immune cells and statistical accuracy (*P* value) of 22 types of immune cells in each sample (the number of permutations was set to 1000). The samples with *P* < 0.05 were retained for subsequent analysis. The difference in immune cells between KIRC tissue and adjacent tissues was analyzed by a two independent-sample *t* test.

### 2.5. Survival Correlation Analysis of Infiltrating Immune Cells in KIRC

Single-factor Cox regression, lasso regression, and multifactor Cox regression analyses were conducted on infiltrating immune cells to build a risk assessment model. The risk survival curve and ROC curve were drawn to evaluate the diagnostic value of the model. The correlation between immune cell subtypes and clinical metastasis was predicted using the Wilcoxon rank-sum test (The clinical research objects are all T staging in TNM). The Kaplan–Meier survival method was used to analyze the survival of all immune cells with different distributions.

### 2.6. Co-expression Analysis of Genes in the ceRNA Network and Immune Cells

The relationship between ceRNAs and 22 types of immune cells was investigated using Pearson's correlation coefficient.

### 2.7. Quantitative Reverse Transcriptase–Polymerase Chain Reaction

Quantitative reverse transcriptase-polymerase chain reaction (qRT-PCR) was used to quantitatively express key genes in the ceRNA network. Clinical tissue cDNA chips were purchased from Shanghai Outdo Biotech Co., Ltd. The chip lot number was cDNA-HKidE030CS01 (15 cases of renal clear cell carcinoma, 1 spot on the cancer/adjacent, the RNA of the frozen sample was reverse-transcribed into cDNA and spotted on a 96-well plate, and the samples covered clinical stage 1, stage 2, and stage 3.). The relative expression levels of lncRNA and mRNA in cancer and adjacent cancer samples of KIRC were detected using the PerfectStart Green qPCR SuperMix (TransGen Biotech, China) following manufacturer's instructions. Primers for lncRNA and mRNA are shown in Table 1. The reaction conditions were predenaturation at 94°C for 30 seconds; 94°C for 5 seconds, 60°C for 30 seconds, 40 cycles; finally, the temperature was lowered to 37°C for 20 min until the reaction was completed. The gene expression profiles downloaded from TCGA database were statistically analyzed in the R software (GDCRNATools, ggplot2, DESeq2, survival, glmnet, survminer). The results of qRT-PCR were analyzed by 2^−△△Ct^ and independent *t* test in IBM SPSS statistics 25.0.

### 2.8. Immunohistochemistry in Clinical Tissues

Clinical tissue chips were purchased from Shanghai Outdo Biotech Co., Ltd. The chip lot number was KidE085CS01 (26 cases of renal clear cell carcinoma, one spot on the carcinoma/adjacent/distal, and 7 metastases, one site per metastases, and the samples covered clinical stage 1, stage 2, and stage 3). Immunohistochemical (IHC) staining was performed using a Leica BOND-MAX auto-stainer (Leica Instrument Co., Ltd., Germany), and the CD4 (EP204) rabbit mAb (48274, Cell Signaling Technology, China) was diluted to 1 : 200. The marker of T follicular helper cells was CD4, respectively [16]. IHC was performed as follows. Briefly, 4 *μ*m thick tissue sections were cut with a microtome, deparaffinized in xylene, dehydrated through graded ethanol (100% and 95%), and rinsed with water. Subsequently, the sections were subjected to heat-induced antigen retrieval and finally loaded onto the Benchmark auto-stainer, and the detection was performed using a bond polymer refine detection kit (Leica Instrument Co., Ltd.).

### 2.9. Immunohistochemical Digital Pathological Analysis

The expression levels of CD4 were estimated by QuPath (open source software for Quantitative Pathology, version 0.2.0) [17]. Each slice included 26 cores of tumor tissues, corresponding peritumor normal renal tissue, distal normal renal tissue, and 7 cores of metastatic renal carcinoma. Digitized IHC microarrays of CD4 were acquired at 100× magnification using an Olympus slide scanner (Olympus motorized BX61VS). The annotation of each core was manually delineated on the pathological slice, while the peritumoral region of the tumoral core and nonspecific staining was excluded. Then, cells within the annotations were detected. The positive cell ratio, Allred score, and H score of each core were calculated to assess the expression levels of CD4. The process of digital analysis of IHC is shown in Supplemental Material 3.

### 2.10. Statistical Analysis

All statistical analyses of the gene expression profiles downloaded from the TCGA database and the immune cells obtained by the CIBERSORT algorithm were performed using the R version 4.0.2 software. The qRT-PCR results were calculated using the 2^−△△Cq^ method and the paired samples *t* test, and the IHC results were analyzed using nonparametric tests. The aforementioned analysis was performed using the IBM SPSS statistics 25.0 software. Only the two-sided *P* value <0.05 was considered to be of statistical significance.

## 3. Results and Discussion

### 3.1. Identification of Differentially Expressed Genes

KIRC transcriptome data of 611 cases (72 cases in the normal group and 539 cases in the cancer group) and miRNA data of 616 cases (71 cases in the normal group and 545 cases in the cancer group) were downloaded from the TCGA database. Gene differential expression analysis revealed 126 DElncRNAs (119 upregulated and 7 downregulated), 25 DEmiRNAs (12 upregulated and 13 downregulated), and 957 DEmRNAs (688 upregulated and 269 downregulated). See Supplementary Material 1 for all differential gene names.

### 3.2. Construction of the ceRNA Network Based on Differentially Expressed Genes

**Figure 2:**
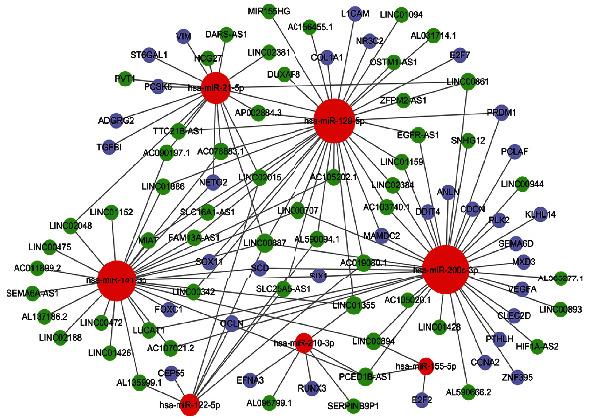
ceRNA network in KIRC.

A ceRNA network, composed of 97 pairs of lncRNA–miRNA and 41 pairs of miRNA–mRNA predicted from the miRcode and StarBase databases, respectively, was constructed (Figure 2), which included 57 lncRNAs, 7 miRNAs, and 34 mRNAs. The lncRNA, miRNA, and mRNA gene names in the ceRNA network are listed in Supplementary Material 2.

### 3.3. Survival Analysis of the ceRNA Network in KIRC

**Figure 3:**
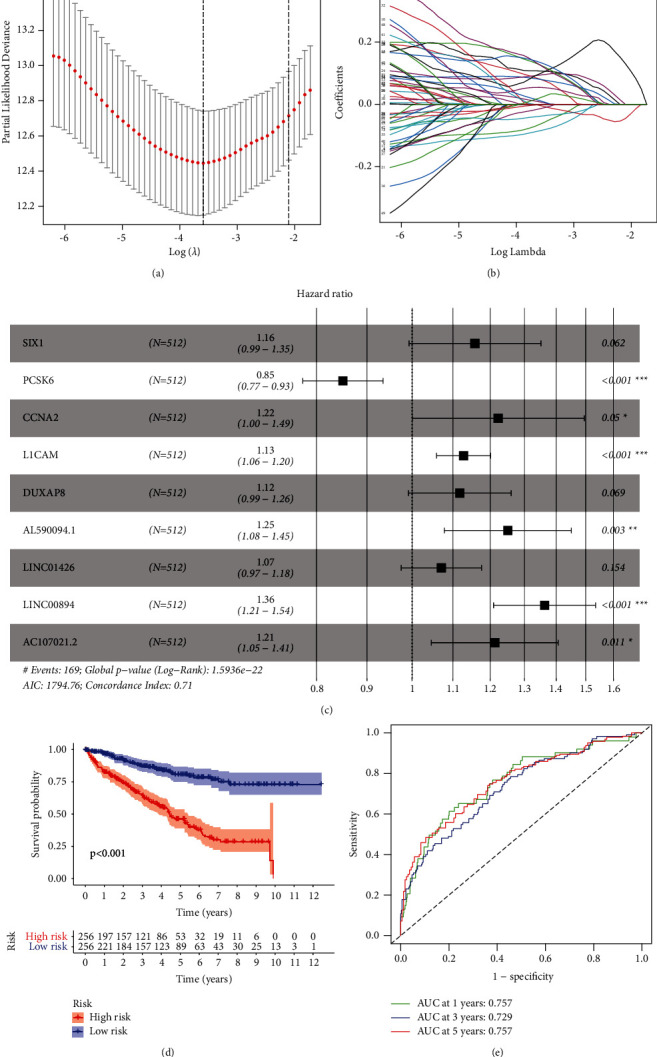
Screening of key genes. ((a)–(c)) Construction of the risk scoring model. (d) Kaplan–Meier risk survival curve of patients with KIRC. (e) ROC curve assessed the diagnostic efficacy of the model.

**Figure 4:**
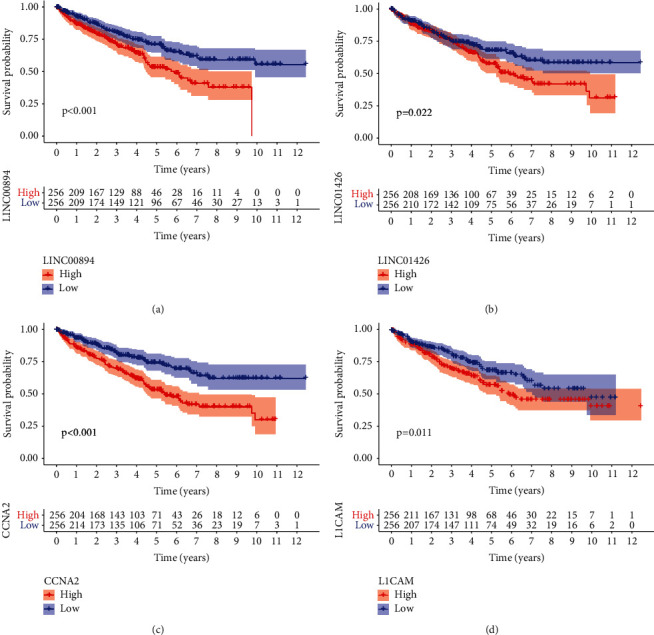
Kaplan–Meier survival curve of key genes. (a) Kaplan–Meier survival curve of LNC00894. (b) Kaplan–Meier survival curve of LINC01426. (c) Kaplan–Meier survival curve of CCNA2. (d) Kaplan–Meier survival curve of L1CAM.

After performing Cox regression analysis on the ceRNA network, nine genes (SIX1, PCSK6, CCNA2, L1 cell adhesion molecule (L1CAM), DUXAP8, AL590094.1, LINC01426, LINC00894, and AC107021.2) were obtained to construct a risk scoring model (Figures 3(a)–3(c)). The risk survival curve indicated that the survival rate of the high-risk group was significantly lower compared with the low-risk group (*P* < 0.001) (Figure 3(d)). The area under the curve (AUC) (1-, 3-, and 5-year survival was 0.757, 0.729, and 0.757, respectively) of the ROC curve indicated a higher diagnostic efficiency of the model (Figure 3(e)). The Kaplan–Meier survival analysis showed that LINC00894, LINC01426, CCNA2, and L1CAM were significant to patients with KIRC (Figures 4(a)–4(d)).

### 3.4. Composition of Immune Cells in KIRC

**Figure 5:**
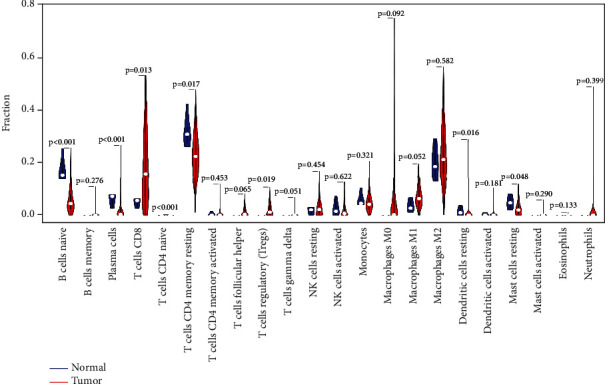
Difference in the proportions of 22 types of immune cells in cancer and adjacent cancer samples.

The CIBERSORT algorithm was used to obtain the immune cell infiltration abundance of all samples, and 223 samples with *P* < 0.05 were retained for subsequent analysis. The heatmap and the violin map showed the difference in the distribution of immune cells between cancer and adjacent cancer samples (Figure 5).

### 3.5. Clinical Correlation Analysis of Immune Cells in KIRC

**Figure 6:**
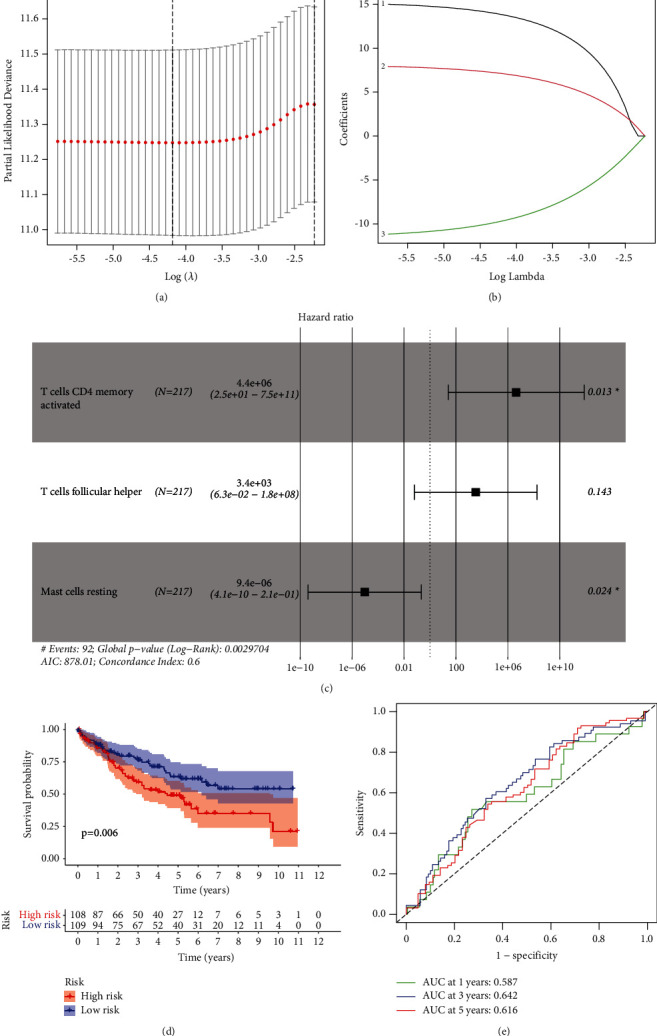
Identification of significant immune cells. ((a)–(c)) Lasso and Cox regression analyses. (d) Risk survival curve of the high- and low-risk groups. (e) ROC curve analysis for predicting the 1-, 3-, and 5-year survival.

Three potential prognostic biomarkers (T-cell CD4 memory activated, T follicular helper cells, and resting mast cells) were regarded as key members among 22 types of immune cells and were integrated into a new multivariable model (Figures 6(a)–6(c)). The risk survival curve suggested that the survival rate of the high-risk group was considerably higher than that of the low-risk group (*P*=0.006) (Figure 6(d)). The ROC curve (AUC of 1-, 3-, and 5-year survival was 0.587, 0.642, and 0.616, respectively) demonstrated the sensitivity and specificity of the model (Figure 6(e)).

**Figure 7:**
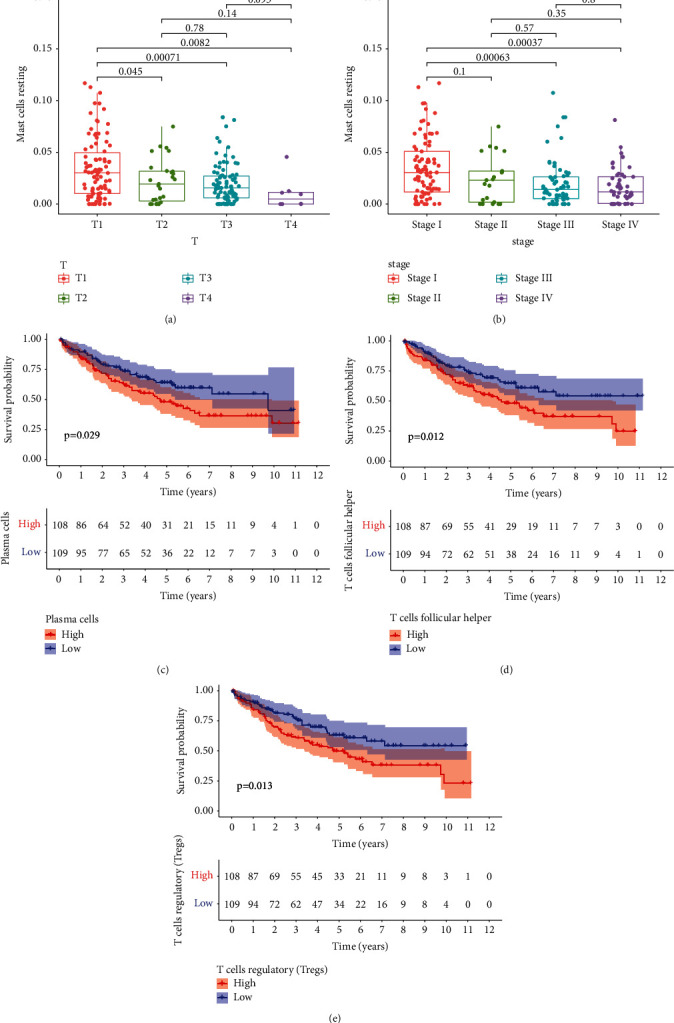
The relationship between significant immune cells and tumor stage and survival. (a) Box plots of T stages of resting mast cells. (b) Box plots of the stage of resting mast cells. (c) Kaplan–Meier survival analysis of plasma cells. (d) Kaplan–Meier survival analysis of T follicular helper cells. (e) Kaplan–Meier survival analysis of regulatory T cells.

The Wilcoxon rank-sum test suggested that resting mast cells had significant differences in T stage and stage (Figures 7(a) and 7(b)). The results of the Kaplan–Meier survival analysis showed that plasma cells (Figure 7(c)), T follicular helper cells (Figure 7(d)), and regulatory T cells (Figure 7(e)) correlated with the survival of patients with KIRC.

### 3.6. Co-Expression Analysis

**Figure 8:**
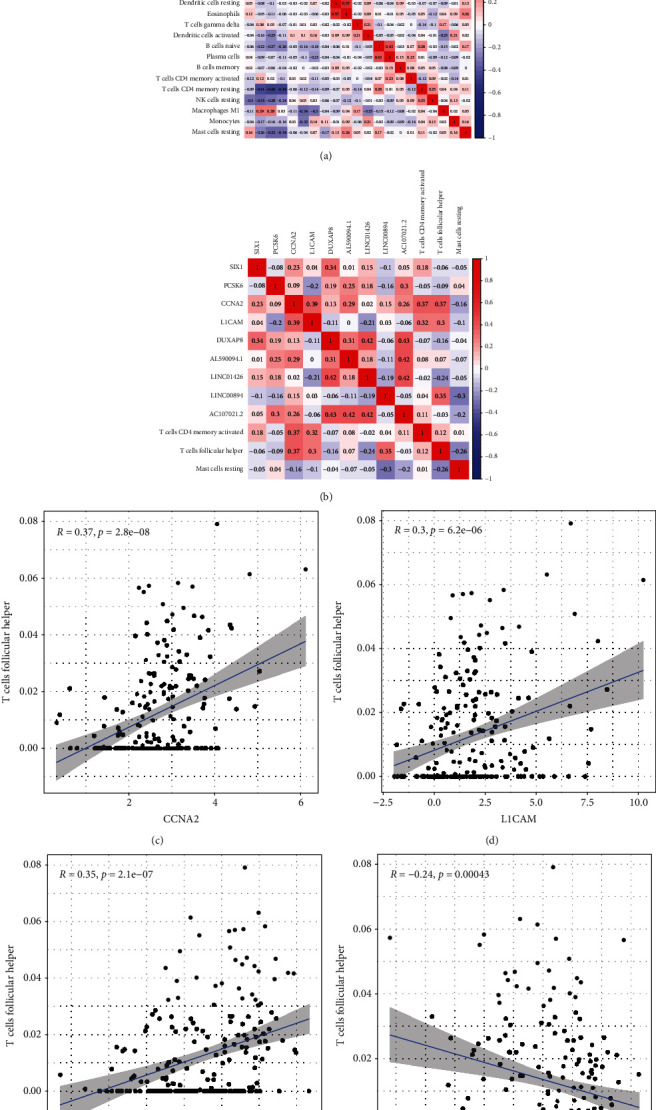
Co-expression analysis between tumor-infiltrating immune cells and key members of the ceRNA network. (a) Co-expression heatmap among immune cells. (b) Co-expression heatmap among two risk scoring models. (c) A positive correlation was found between CCNA2 and T follicular helper cells (*R* = 0.37, *P* < 0.001). (d). A positive correlation was found between L1CAM and T follicular helper cells (*R* = 0.30, *P* < 0.001). (e) A positive correlation was found between LINC00894 and T follicular helper cells (*R* = 0.35, *P* < 0.001) 0.001. (f) A negative correlation was found between LINC01426 and T follicular helper cells (*R* = −0.24, *P* < 0.001).

Important co-expression patterns between immune cells (Figure 8(a)), key members of the ceRNA network, and co-expression of some important co-expression patterns of key members of immune cells (Figure 8(b)) were analyzed. The results showed a positive correlation between CCNA2 and T follicular helper cells (*R* = 0.37, *P* < 0.001) (Figure 8(c)), between L1CAM and T follicular helper cells (*R* = 0.30, *P* < 0.001) (Figure 8(d)), between LINC00894 and T follicular helper cells (*R* = 0.35, *P* < 0.001) (Figure 8(e)), and between LINC01426 and T follicular helper cells (*R* = 0.24, *P* < 0.001) (Figure 8(f)). These results indicated that their relationship might be a biomarker for the diagnosis and prognosis of KIRC.

## 4. Results of Clinical Tissue Specimen Verification

**Figure 9:**
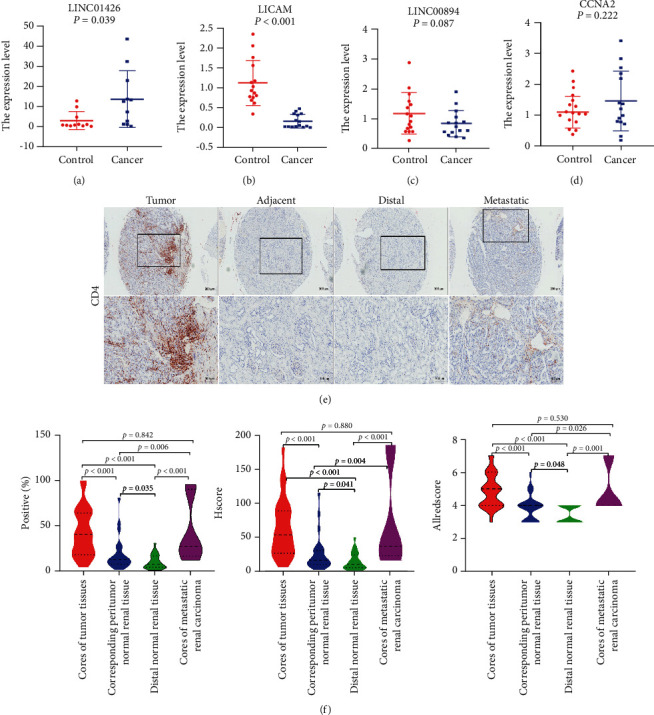
Verification in clinical samples. ((a)-(d)) Differentially expressed CCNA2, L1CAM, LINC01426, and LINC00894 in the cancerous and paracancerous groups. LINC01426 was upregulated, L1CAM was downregulated in kidney cancer tissues (*P* < 0.05), LINC00894 and CCNA2 had no difference (*P* > 0.05). (e) IHC results (including 26 cores of tumor tissues, corresponding to peritumor normal renal tissue, distal normal renal tissue, and seven cores of metastatic renal carcinoma). (f) IHC results using nonparametric tests. T follicular helper cells (CD4 marker positive) displayed difference in the core of the tumor tissues, and the corresponding normal kidney tissue adjacent to the cancer, the distal normal kidney tissue, and the core of metastatic renal cancer (*P* < 0.05).

The qRT-PCR results showed that lncRNA LINC01426 was upregulated while mRNA L1CAM was downregulated in kidney cancer tissues, which was consistent with the expression pattern in the TCGA database (*P* < 0.05) (Figures 9(a) and 9(b)). However, there was no difference in expression levels of lncRNA LINC00894 and CCNA2 mRNA in renal cancer tissue and adjacent tissue (*P* > 0.05) (Figures 9(c) and 9(d)). The IHC results showed that the level of T follicular helper cells (CD4 marker positive) was the highest in the core of tumor tissues, which was significantly different from the corresponding normal renal tissue adjacent to cancer, distal normal renal tissue and metastatic renal cancer core tissue (*P* < 0.05). The level of T-follicular helper cells is the second highest in the metastatic renal cell carcinoma core tissue, which was a significant difference between adjacent normal renal tissues and distal normal renal tissue (*P* < 0.05) (Figures 9(e) and 9(f)). The results demonstrated that the expression characteristics of lncRNA LINC01426 and mRNA L1CAM as well as T follicular helper cells were verified in clinical specimens. This suggested that a co-expression relationship existed between LINC01426, L1CAM, and T follicular helper cells, and they might be used as biomarkers for early diagnosis and prognostic evaluation of KIRC.

## 5. Conclusions

At present, many patients with KIRC whose diagnoses were mainly based on the clinical symptoms and imaging methods have already developed distant metastases at this time; the recurrence rate after surgical radical treatment was high [18]. In recent years, a large number of researchers have focused on exploring the mechanism of genes, tumor-infiltrating immune cells, and the interaction between the two in the occurrence, development, metastasis, and prognosis of KIRC, indicating that genes and immune cells were closely related to tumors, and provided direction for the diagnosis and treatment of KIRC in the future [19–22]. For example, Zhengyan Chang et al. separately constructed the risk scoring model of the ceRNA network and infiltrating immune cells in colon cancer and found that T follicular helper cells and hsa-miR-125b-5p, macrophages M0 and hsa-miR-125b, and macrophages M0 and FAS might become potential biomarkers through co-expression analysis, and this conclusion was verified in clinical tissues [23]. This research model based on bone metastatic melanoma, gastric cancer, breast cancer bone metastasis, mesothelioma bone metastasis, and other tumors has been adopted by various studies [24–26]. The present study also used this model and used bioinformatics analysis to identify co-expression regulation relationships among LINC01426, LINC00894, CCNA2, L1CAM, and T follicular helper cells. These key members might become KIRC diagnostic and therapeutic potential biomarkers.

LINC01426 was upregulated in renal clear cell carcinoma tissues and its overexpression was correlated with a disappointing prognosis [27]. So far, the data on L1CAM expression in renal clear cell carcinoma were contradictory; studies have shown that cell adhesion, metastasis, and invasion abilities were significantly increased with the upregulation of L1CAM expression in KIRC, and in turn, the downregulation of LICAM expression decreased the proliferation of renal cancer cell and reduced the expression of cyclin D1 [28, 29]. However, this just illustrated the importance of L1CAM in the progression of KIRC. T follicular helper cells are a specialized subset of CD4+ T cells that were first identified in tonsils in humans. They play an essential role in forming germinal centers, and Xiaoliang Hua et al. found that tumors from high-risk patients had a higher relative abundance of T follicular helper cells [30]. The present study confirmed the high expression of LINC01426, L1CAM, and tumor infiltration of T follicular helper cells because these cells were closely related to the clinical and prognostic prediction of KIRC. Thus, these cells were found more likely to be KIRC biomarkers.

In conclusion, the present bio-report analysis indicated a relationship among LINC01426, L1CAM, and T follicular helper cells, which was meaningful. As it is difficult to detect the patient's immune cells, the abundance of T follicular helper cells in KIRC was determined by detecting the expression levels of LINC01426 and L1CAM, which have a co-expression relationship to provide new prospects for the early diagnosis of KIRC so as to develop new therapeutic drugs.

## Figures and Tables

**Table 1:** Primers for lncRNA and mRNA.

Gene		Primer sequence (5′-3′)
LINC01426	F	ACTGTCCCTTTATCACCCTT
	R	CGTTGAAGCTCCTTGCCTAT

LINC00894	F	GCTCCTGGGACCACATTA
	R	TAGTACAAGCTGAGGCAAA

L1CAM	F	TGGGAATGTAAATACACCGTGAC
	R	GCACAGGCATACAGGGAGG

CCNA2	F	ATGAGCATGTCACCGTTCC
	R	AAGCCAGGGCATCTTCACG

F : forward, R : reverse.

## Data Availability

The datasets used and/or analyzed in the present study are available from the corresponding author on reasonable request.

## Supplementary Materials

Supplementary Materials Differentially expressed lncRNA, miRNAs, and mRNAs were put in Supplementary Material 1. Gene ID of lncRNAs, miRNAs and mRNAs in the ceRNA network were displayed in Supplementary Material 2. For a comprehensive digital IHC image analysis with Qupath, please refer to the protocol in Supplementary Material 3.
