# Supplementary material for: Correlation between Genes of the ceRNA Network and Tumor-Infiltrating Immune Cells and Their Biomarker Screening in Kidney Renal Clear Cell Carcinoma
Source: J Oncol. 2022 Aug 29;2022:4084461. doi: 10.1155/2022/4084461 (PMC9444395; doi:10.1155/2022/4084461)
Supplement: Supplementary Materials — Differentially expressed lncRNA, miRNAs, and mRNAs were put in Supplementary Material 1. Gene ID of lncRNAs, miRNAs and mRNAs in the ceRNA network were displayed in Supplementary Material 2. For a comprehensive digital IHC image analysis with Qupath, please refer to the protocol in Supplementary Material 3. [file 4084461.f1.zip › Supplementary Material 1.docx]

Table: Gene differential expression analysis revealed 126 DElncRNAs (119 upregulated and 7 downregulated)

| symbol | group | baseMean | logFC | lfcSE | stat | PValue | FDR |
| --- | --- | --- | --- | --- | --- | --- | --- |
| PVT1 | long_non_coding | 878.8177 | 4.63724 | 0.120406 | 38.51341 | 0 | 0 |
| TTC21B-AS1 | long_non_coding | 1726.4 | 8.447422 | 0.238646 | 35.39728 | 1.88E-274 | 9.62E-271 |
| AC019080.1 | long_non_coding | 498.2108 | -3.62906 | 0.103433 | -35.0859 | 1.10E-269 | 4.24E-266 |
| LINC00887 | long_non_coding | 2203.761 | 5.584534 | 0.181365 | 30.79168 | 3.39E-208 | 4.33E-205 |
| AC010655.2 | long_non_coding | 95.67856 | 5.320731 | 0.194283 | 27.3865 | 3.97E-165 | 1.56E-162 |
| AC078883.1 | long_non_coding | 199.1759 | 2.031285 | 0.076403 | 26.58641 | 9.75E-156 | 3.18E-153 |
| AC026369.2 | long_non_coding | 320.9996 | 3.662067 | 0.138612 | 26.41964 | 8.15E-154 | 2.61E-151 |
| DGCR9 | long_non_coding | 418.9277 | 3.945746 | 0.149561 | 26.38225 | 2.19E-153 | 6.86E-151 |
| LINC02048 | long_non_coding | 110.9305 | 5.550968 | 0.21122 | 26.28046 | 3.21E-152 | 9.66E-150 |
| LINC02348 | long_non_coding | 195.3098 | 6.009671 | 0.228697 | 26.27788 | 3.43E-152 | 1.01E-149 |
| DARS-AS1 | long_non_coding | 344.2068 | 3.124073 | 0.119444 | 26.15522 | 8.60E-151 | 2.40E-148 |
| AC079760.2 | long_non_coding | 276.0565 | 5.276925 | 0.201771 | 26.153 | 9.11E-151 | 2.50E-148 |
| SLC16A1-AS1 | long_non_coding | 290.4738 | 3.152966 | 0.120967 | 26.06469 | 9.17E-150 | 2.47E-147 |
| LINC00462 | long_non_coding | 772.8762 | 6.365099 | 0.244596 | 26.02287 | 2.73E-149 | 6.98E-147 |
| MIR210HG | long_non_coding | 1384.06 | 3.265502 | 0.125763 | 25.96559 | 1.21E-148 | 3.05E-146 |
| AC097534.2 | long_non_coding | 477.731 | 2.892083 | 0.111857 | 25.85513 | 2.13E-147 | 5.03E-145 |
| AL590644.1 | long_non_coding | 358.3622 | 7.584131 | 0.293456 | 25.84417 | 2.83E-147 | 6.58E-145 |
| AC019069.1 | long_non_coding | 149.6384 | 3.388998 | 0.132507 | 25.57605 | 2.82E-144 | 6.09E-142 |
| GAS6-AS1 | long_non_coding | 721.2722 | 4.124075 | 0.162496 | 25.37961 | 4.24E-142 | 8.44E-140 |
| LINC02188 | long_non_coding | 825.1894 | 3.807586 | 0.155054 | 24.55651 | 3.68E-133 | 5.83E-131 |
| LINC01587 | long_non_coding | 143.3349 | 5.159497 | 0.211184 | 24.43124 | 7.97E-132 | 1.21E-129 |
| AC007406.3 | long_non_coding | 210.0425 | 3.373438 | 0.13869 | 24.32352 | 1.11E-130 | 1.63E-128 |
| HIF1A-AS2 | long_non_coding | 150.8957 | 4.073667 | 0.172517 | 23.61306 | 2.83E-123 | 3.39E-121 |
| OSTM1-AS1 | long_non_coding | 353.4498 | 7.320045 | 0.313022 | 23.38511 | 6.06E-121 | 6.89E-119 |
| AL357060.2 | long_non_coding | 143.6298 | 2.327136 | 0.099782 | 23.32218 | 2.64E-120 | 2.90E-118 |
| AL096799.1 | long_non_coding | 106.574 | 5.094139 | 0.218431 | 23.32155 | 2.68E-120 | 2.92E-118 |
| AC138207.5 | long_non_coding | 103.1277 | 2.413267 | 0.104019 | 23.20021 | 4.53E-119 | 4.86E-117 |
| AP000439.2 | long_non_coding | 421.241 | 6.256995 | 0.271174 | 23.0737 | 8.51E-118 | 8.77E-116 |
| LINC01094 | long_non_coding | 301.5018 | 2.792948 | 0.125324 | 22.2859 | 5.06E-110 | 4.42E-108 |
| LUCAT1 | long_non_coding | 211.8102 | 5.5358 | 0.249429 | 22.1939 | 3.93E-109 | 3.30E-107 |
| AL109615.3 | long_non_coding | 537.3334 | 2.844129 | 0.130557 | 21.78457 | 3.25E-105 | 2.39E-103 |
| AC083862.2 | long_non_coding | 210.7466 | 2.058635 | 0.095099 | 21.64726 | 6.45E-104 | 4.54E-102 |
| LINP1 | long_non_coding | 98.8638 | 3.624429 | 0.170435 | 21.2658 | 2.35E-100 | 1.43E-98 |
| EGFR-AS1 | long_non_coding | 821.6337 | 5.86606 | 0.276692 | 21.20072 | 9.41E-100 | 5.53E-98 |
| AC008760.2 | long_non_coding | 459.7668 | 5.084908 | 0.241529 | 21.053 | 2.15E-98 | 1.21E-96 |
| SNHG12 | long_non_coding | 838.734 | 2.988715 | 0.143015 | 20.89795 | 5.59E-97 | 2.95E-95 |
| PCED1B-AS1 | long_non_coding | 506.6271 | 2.695783 | 0.129061 | 20.88772 | 6.93E-97 | 3.63E-95 |
| AC105020.1 | long_non_coding | 138.9353 | 3.543127 | 0.170665 | 20.76068 | 9.82E-96 | 4.94E-94 |
| MIAT | long_non_coding | 428.3064 | 4.414886 | 0.218104 | 20.24212 | 4.17E-91 | 1.81E-89 |
| SLC25A5-AS1 | long_non_coding | 114.5794 | -2.021 | 0.099926 | -20.225 | 5.89E-91 | 2.56E-89 |
| LINC00944 | long_non_coding | 109.0787 | 3.882843 | 0.192103 | 20.21227 | 7.63E-91 | 3.27E-89 |
| TRG-AS1 | long_non_coding | 150.6781 | 2.648359 | 0.131209 | 20.18421 | 1.35E-90 | 5.73E-89 |
| AL021328.1 | long_non_coding | 162.0811 | 3.689584 | 0.183329 | 20.12543 | 4.42E-90 | 1.83E-88 |
| AC008105.3 | long_non_coding | 153.4534 | 3.940225 | 0.198675 | 19.8325 | 1.56E-87 | 5.98E-86 |
| AL590666.2 | long_non_coding | 134.3248 | 2.809676 | 0.141673 | 19.83209 | 1.57E-87 | 6.01E-86 |
| LINC00475 | long_non_coding | 164.1348 | 4.140485 | 0.209101 | 19.8014 | 2.90E-87 | 1.09E-85 |
| AL137186.2 | long_non_coding | 187.7254 | 2.258996 | 0.114757 | 19.685 | 2.90E-86 | 1.06E-84 |
| AC011899.2 | long_non_coding | 151.5166 | 2.750509 | 0.139916 | 19.65827 | 4.91E-86 | 1.79E-84 |
| AC107021.2 | long_non_coding | 269.1818 | 2.817384 | 0.144135 | 19.54685 | 4.39E-85 | 1.54E-83 |
| MMP25-AS1 | long_non_coding | 534.8009 | 2.381665 | 0.122735 | 19.40492 | 7.01E-84 | 2.36E-82 |
| AC018553.1 | long_non_coding | 299.7443 | 2.643397 | 0.139202 | 18.98968 | 2.08E-80 | 6.29E-79 |
| LINC01759 | long_non_coding | 81.90752 | 2.098545 | 0.1108 | 18.93997 | 5.34E-80 | 1.59E-78 |
| MIR155HG | long_non_coding | 213.3281 | 3.332455 | 0.176614 | 18.86853 | 2.07E-79 | 6.01E-78 |
| AC090197.1 | long_non_coding | 123.2118 | 2.126347 | 0.113556 | 18.72508 | 3.09E-78 | 8.68E-77 |
| AL731533.2 | long_non_coding | 136.87 | 2.195034 | 0.118101 | 18.58612 | 4.16E-77 | 1.12E-75 |
| AC156455.1 | long_non_coding | 190.3513 | 3.070197 | 0.166649 | 18.42315 | 8.57E-76 | 2.21E-74 |
| AP002884.3 | long_non_coding | 654.3113 | 2.529738 | 0.137749 | 18.36481 | 2.51E-75 | 6.40E-74 |
| LINC01428 | long_non_coding | 117.0813 | 3.698204 | 0.201463 | 18.35673 | 2.92E-75 | 7.40E-74 |
| AL117335.1 | long_non_coding | 171.6604 | 2.730866 | 0.149373 | 18.28221 | 1.15E-74 | 2.86E-73 |
| HCG27 | long_non_coding | 184.3321 | 2.868692 | 0.157011 | 18.2707 | 1.42E-74 | 3.52E-73 |
| LINC01235 | long_non_coding | 565.2923 | 3.69386 | 0.203229 | 18.17584 | 8.02E-74 | 1.95E-72 |
| LINC00472 | long_non_coding | 298.9855 | -2.19235 | 0.120649 | -18.1713 | 8.71E-74 | 2.11E-72 |
| AC040160.1 | long_non_coding | 72.66383 | 2.122372 | 0.117048 | 18.13253 | 1.76E-73 | 4.25E-72 |
| AC147651.3 | long_non_coding | 113.9173 | 2.393408 | 0.132266 | 18.09535 | 3.47E-73 | 8.18E-72 |
| AC023024.1 | long_non_coding | 242.466 | 2.855496 | 0.157899 | 18.08431 | 4.24E-73 | 9.91E-72 |
| LINC00861 | long_non_coding | 204.242 | 2.913232 | 0.162428 | 17.93553 | 6.23E-72 | 1.40E-70 |
| AC073218.1 | long_non_coding | 129.6582 | 3.113095 | 0.174417 | 17.8486 | 2.96E-71 | 6.45E-70 |
| AP006284.1 | long_non_coding | 499.2789 | 3.66463 | 0.210247 | 17.43015 | 4.87E-68 | 9.61E-67 |
| LINC02381 | long_non_coding | 754.0924 | -2.23615 | 0.128928 | -17.3442 | 2.18E-67 | 4.21E-66 |
| AL365181.3 | long_non_coding | 455.5151 | 2.572809 | 0.14982 | 17.17264 | 4.26E-66 | 7.81E-65 |
| PP7080 | long_non_coding | 2194.819 | -2.43767 | 0.142187 | -17.1441 | 6.96E-66 | 1.27E-64 |
| FAM13A-AS1 | long_non_coding | 275.6763 | 2.414923 | 0.14099 | 17.12839 | 9.11E-66 | 1.65E-64 |
| SMIM25 | long_non_coding | 202.3838 | 2.603676 | 0.152615 | 17.06037 | 2.93E-65 | 5.16E-64 |
| PSORS1C3 | long_non_coding | 430.6744 | 3.10378 | 0.183419 | 16.92177 | 3.11E-64 | 5.32E-63 |
| AC019117.2 | long_non_coding | 1022.097 | 5.247889 | 0.310179 | 16.91892 | 3.26E-64 | 5.56E-63 |
| AC073486.1 | long_non_coding | 2698.969 | 3.287446 | 0.195924 | 16.77915 | 3.47E-63 | 5.68E-62 |
| LINC01268 | long_non_coding | 317.071 | 2.35867 | 0.140718 | 16.76165 | 4.66E-63 | 7.60E-62 |
| LINC00173 | long_non_coding | 201.9073 | 3.244514 | 0.19372 | 16.74844 | 5.81E-63 | 9.47E-62 |
| LINC01159 | long_non_coding | 127.8401 | -2.34629 | 0.140687 | -16.6774 | 1.91E-62 | 3.09E-61 |
| AL080317.1 | long_non_coding | 311.6234 | 2.79351 | 0.167659 | 16.66188 | 2.48E-62 | 3.99E-61 |
| AC022144.1 | long_non_coding | 492.7502 | 2.392142 | 0.14498 | 16.49975 | 3.68E-61 | 5.72E-60 |
| AL365181.2 | long_non_coding | 175.9705 | 3.015655 | 0.182877 | 16.49008 | 4.32E-61 | 6.70E-60 |
| LINC00342 | long_non_coding | 421.205 | 2.366489 | 0.144167 | 16.41491 | 1.50E-60 | 2.29E-59 |
| AC078864.1 | long_non_coding | 238.3052 | 2.793086 | 0.171845 | 16.25349 | 2.11E-59 | 3.09E-58 |
| ITGB2-AS1 | long_non_coding | 209.9617 | 2.895722 | 0.180591 | 16.03471 | 7.31E-58 | 1.02E-56 |
| AC022509.2 | long_non_coding | 223.0236 | 2.837869 | 0.177209 | 16.01424 | 1.02E-57 | 1.42E-56 |
| AC136475.3 | long_non_coding | 577.7615 | 3.952096 | 0.249697 | 15.82758 | 2.01E-56 | 2.67E-55 |
| DUXAP8 | long_non_coding | 150.026 | 2.815095 | 0.180472 | 15.59851 | 7.45E-55 | 9.50E-54 |
| AC103740.1 | long_non_coding | 215.0767 | 2.156739 | 0.138659 | 15.55426 | 1.49E-54 | 1.87E-53 |
| AL590094.1 | long_non_coding | 190.5123 | 2.278849 | 0.14672 | 15.53198 | 2.11E-54 | 2.64E-53 |
| LINC01152 | long_non_coding | 115.2411 | 2.791783 | 0.180282 | 15.48567 | 4.34E-54 | 5.34E-53 |
| N4BP2L2-IT2 | long_non_coding | 161.0169 | 2.075098 | 0.135673 | 15.29486 | 8.27E-53 | 9.86E-52 |
| AC009549.1 | long_non_coding | 132.3939 | 2.457063 | 0.161479 | 15.21599 | 2.77E-52 | 3.23E-51 |
| SEMA6A-AS1 | long_non_coding | 94.5255 | 2.121386 | 0.139867 | 15.16715 | 5.84E-52 | 6.72E-51 |
| AL031714.1 | long_non_coding | 184.387 | 2.040887 | 0.136738 | 14.92548 | 2.25E-50 | 2.46E-49 |
| SLC9A3-AS1 | long_non_coding | 3247.198 | 3.056514 | 0.208676 | 14.64714 | 1.40E-48 | 1.44E-47 |
| AC092123.2 | long_non_coding | 204.0302 | 2.010849 | 0.137429 | 14.63195 | 1.76E-48 | 1.80E-47 |
| ARHGAP27P1-BPTFP1-KPNA2P3 | long_non_coding | 211.4825 | 2.047312 | 0.142027 | 14.41491 | 4.17E-47 | 4.04E-46 |
| AL365277.1 | long_non_coding | 235.3134 | 2.061143 | 0.145 | 14.21478 | 7.42E-46 | 6.87E-45 |
| LINC00707 | long_non_coding | 180.2892 | 2.828902 | 0.199268 | 14.19648 | 9.63E-46 | 8.87E-45 |
| AC012615.6 | long_non_coding | 101.7311 | 2.096623 | 0.149724 | 14.00329 | 1.49E-44 | 1.33E-43 |
| HLA-DQB1-AS1 | long_non_coding | 138.6365 | 2.253065 | 0.161255 | 13.97209 | 2.31E-44 | 2.05E-43 |
| LINC01355 | long_non_coding | 304.4622 | 2.099796 | 0.154222 | 13.61541 | 3.24E-42 | 2.65E-41 |
| SERPINB9P1 | long_non_coding | 220.4723 | 2.164873 | 0.161737 | 13.38514 | 7.39E-41 | 5.78E-40 |
| ASMTL-AS1 | long_non_coding | 231.3642 | 2.693486 | 0.20521 | 13.12553 | 2.35E-39 | 1.75E-38 |
| PTOV1-AS2 | long_non_coding | 147.4372 | 2.068107 | 0.157628 | 13.12022 | 2.52E-39 | 1.87E-38 |
| LINC02015 | long_non_coding | 348.1395 | 2.600049 | 0.201247 | 12.91968 | 3.49E-38 | 2.49E-37 |
| AL590999.1 | long_non_coding | 238.9375 | 2.676356 | 0.207735 | 12.8835 | 5.58E-38 | 3.95E-37 |
| LINC00893 | long_non_coding | 146.3877 | 2.164955 | 0.169737 | 12.75475 | 2.93E-37 | 2.03E-36 |
| AL135999.1 | long_non_coding | 148.2708 | 2.007271 | 0.160438 | 12.51118 | 6.48E-36 | 4.27E-35 |
| AC104031.1 | long_non_coding | 126.7836 | 2.297497 | 0.185097 | 12.41238 | 2.24E-35 | 1.45E-34 |
| ZFPM2-AS1 | long_non_coding | 194.7314 | 2.076315 | 0.169419 | 12.25552 | 1.57E-34 | 9.79E-34 |
| AL645608.8 | long_non_coding | 199.895 | 2.075953 | 0.170326 | 12.18813 | 3.60E-34 | 2.20E-33 |
| AC021744.1 | long_non_coding | 469.8208 | 3.149241 | 0.262219 | 12.00999 | 3.15E-33 | 1.85E-32 |
| LINC00894 | long_non_coding | 164.2964 | 2.050855 | 0.172481 | 11.89031 | 1.33E-32 | 7.64E-32 |
| LINC01426 | long_non_coding | 718.7384 | 2.530455 | 0.215316 | 11.75227 | 6.87E-32 | 3.86E-31 |
| LINC02384 | long_non_coding | 723.1891 | 2.490095 | 0.212709 | 11.70659 | 1.18E-31 | 6.56E-31 |
| AC104964.3 | long_non_coding | 120.6986 | 2.003188 | 0.172393 | 11.61988 | 3.27E-31 | 1.79E-30 |
| AC105202.1 | long_non_coding | 214.5624 | 2.039967 | 0.182566 | 11.17387 | 5.47E-29 | 2.75E-28 |
| AL157931.1 | long_non_coding | 288.294 | 2.554074 | 0.244391 | 10.45077 | 1.45E-25 | 6.32E-25 |
| LINC01886 | long_non_coding | 135.3455 | 2.287458 | 0.219274 | 10.43197 | 1.77E-25 | 7.68E-25 |
| AC124854.1 | long_non_coding | 218.199 | 2.295113 | 0.224231 | 10.23548 | 1.38E-24 | 5.71E-24 |
| AC003984.1 | long_non_coding | 256.5186 | 2.242541 | 0.222743 | 10.06784 | 7.66E-24 | 3.07E-23 |
| AC005035.1 | long_non_coding | 1053.016 | 2.910595 | 0.296508 | 9.816245 | 9.58E-23 | 3.68E-22 |
| LINC01510 | long_non_coding | 166.2353 | -2.07579 | 0.227449 | -9.12641 | 7.08E-20 | 2.42E-19 |
| AC104126.1 | long_non_coding | 201.3434 | 2.111674 | 0.266657 | 7.919072 | 2.39E-15 | 6.60E-15 |

Table: Gene differential expression analysis revealed 25 DEmiRNAs (12 upregulated and 13 downregulated)

| symbol | group | baseMean | logFC | lfcSE | stat | PValue | FDR |
| --- | --- | --- | --- | --- | --- | --- | --- |
| hsa-miR-210-3p | MicroRNA | 7886.061 | 3.039134 | 0.122209 | 24.86828 | 1.64E-136 | 6.87E-134 |
| hsa-miR-122-5p | MicroRNA | 60.3687 | 6.472347 | 0.274242 | 23.60083 | 3.78E-123 | 7.92E-121 |
| hsa-miR-155-5p | MicroRNA | 1532.093 | 3.496995 | 0.153517 | 22.77918 | 7.38E-115 | 1.03E-112 |
| hsa-miR-508-3p | MicroRNA | 117.7565 | -4.49079 | 0.203884 | -22.0262 | 1.61E-107 | 1.69E-105 |
| hsa-miR-21-5p | MicroRNA | 445894.2 | 2.172933 | 0.100839 | 21.54847 | 5.47E-103 | 4.59E-101 |
| hsa-miR-514a-3p | MicroRNA | 92.08083 | -4.50248 | 0.213874 | -21.052 | 2.19E-98 | 1.53E-96 |
| hsa-miR-532-3p | MicroRNA | 140.9219 | -2.18061 | 0.105692 | -20.6317 | 1.43E-94 | 8.53E-93 |
| hsa-miR-584-5p | MicroRNA | 121.9996 | 2.111639 | 0.105713 | 19.97522 | 9.05E-89 | 4.74E-87 |
| hsa-miR-509-3p | MicroRNA | 28.84546 | -3.19297 | 0.168366 | -18.9644 | 3.36E-80 | 1.28E-78 |
| hsa-miR-362-5p | MicroRNA | 55.75492 | -2.81267 | 0.155004 | -18.1459 | 1.38E-73 | 4.14E-72 |
| hsa-miR-188-5p | MicroRNA | 5.992613 | -2.08419 | 0.11826 | -17.6237 | 1.62E-69 | 4.24E-68 |
| hsa-miR-224-5p | MicroRNA | 79.28893 | 2.73459 | 0.165608 | 16.51239 | 2.99E-61 | 6.59E-60 |
| hsa-miR-592 | MicroRNA | 62.85847 | 3.063079 | 0.186466 | 16.42697 | 1.23E-60 | 2.57E-59 |
| hsa-miR-129-5p | MicroRNA | 15.86846 | -3.97774 | 0.24807 | -16.0348 | 7.30E-58 | 1.46E-56 |
| hsa-miR-885-5p | MicroRNA | 42.21797 | 3.648476 | 0.231175 | 15.78228 | 4.12E-56 | 7.51E-55 |
| hsa-miR-4772-3p | MicroRNA | 9.289761 | 2.034108 | 0.150736 | 13.49449 | 1.69E-41 | 1.86E-40 |
| hsa-miR-188-3p | MicroRNA | 5.786101 | -2.02045 | 0.15391 | -13.1275 | 2.29E-39 | 2.23E-38 |
| hsa-miR-210-5p | MicroRNA | 3.987157 | 2.412587 | 0.20872 | 11.55897 | 6.65E-31 | 4.16E-30 |
| hsa-miR-6509-5p | MicroRNA | 3.947267 | 2.220853 | 0.20465 | 10.85197 | 1.95E-27 | 1.06E-26 |
| hsa-miR-200c-3p | MicroRNA | 1069.547 | -3.18527 | 0.294647 | -10.8105 | 3.07E-27 | 1.65E-26 |
| hsa-miR-3615 | MicroRNA | 3.89846 | 2.0407 | 0.212585 | 9.599462 | 8.04E-22 | 3.51E-21 |
| hsa-miR-1251-5p | MicroRNA | 24.07423 | -2.30386 | 0.278088 | -8.28464 | 1.18E-16 | 3.91E-16 |
| hsa-miR-203b-3p | MicroRNA | 17.05148 | -2.53611 | 0.310986 | -8.15506 | 3.49E-16 | 1.12E-15 |
| hsa-miR-141-3p | MicroRNA | 161.7599 | -2.61475 | 0.339236 | -7.70776 | 1.28E-14 | 3.75E-14 |
| hsa-miR-141-5p | MicroRNA | 70.25949 | -2.12443 | 0.381455 | -5.56928 | 2.56E-08 | 5.08E-08 |

Table: Gene differential expression analysis revealed 957 DEmiRNAs (688 upregulated and 269 downregulated)

| symbol | group | baseMean | logFC | lfcSE | stat | PValue | FDR |
| --- | --- | --- | --- | --- | --- | --- | --- |
| MFSD4A | protein_coding | 3860.218 | -5.24748 | 0.126654 | -41.4317 | 0 | 0 |
| SPAG4 | protein_coding | 2576.81 | 4.109429 | 0.117824 | 34.87781 | 1.61E-266 | 4.95E-263 |
| NDUFA4L2 | protein_coding | 86522.63 | 5.800707 | 0.172082 | 33.709 | 4.27E-249 | 1.09E-245 |
| EGLN3 | protein_coding | 29830.7 | 4.247944 | 0.129677 | 32.75792 | 2.34E-235 | 5.14E-232 |
| GABRD | protein_coding | 860.8006 | 5.261593 | 0.161744 | 32.53033 | 3.97E-232 | 7.63E-229 |
| HILPDA | protein_coding | 11949.33 | 4.742063 | 0.146584 | 32.35053 | 1.36E-229 | 2.33E-226 |
| NOL3 | protein_coding | 6093.455 | 3.425153 | 0.107356 | 31.90468 | 2.30E-223 | 3.53E-220 |
| SCARB1 | protein_coding | 13982.83 | 4.081006 | 0.130894 | 31.17786 | 2.13E-213 | 2.97E-210 |
| COL23A1 | protein_coding | 11370.16 | 5.181354 | 0.168414 | 30.76556 | 7.57E-208 | 8.94E-205 |
| SIM2 | protein_coding | 378.1356 | -4.50363 | 0.147707 | -30.4903 | 3.50E-204 | 3.84E-201 |
| CA9 | protein_coding | 11898.01 | 6.047035 | 0.198373 | 30.48317 | 4.36E-204 | 4.46E-201 |
| DDB2 | protein_coding | 2888.408 | 2.028416 | 0.066574 | 30.46849 | 6.82E-204 | 6.54E-201 |
| STC2 | protein_coding | 7456.098 | 4.252172 | 0.140515 | 30.26127 | 3.71E-201 | 3.35E-198 |
| HSF4 | protein_coding | 4265.417 | 5.714658 | 0.190094 | 30.06222 | 1.51E-198 | 1.29E-195 |
| CDCA2 | protein_coding | 1180.456 | 4.690685 | 0.15635 | 30.00117 | 9.47E-198 | 7.65E-195 |
| CDKN2A | protein_coding | 444.8251 | 4.950879 | 0.166001 | 29.82436 | 1.89E-195 | 1.45E-192 |
| ANGPTL4 | protein_coding | 44987.34 | 4.947724 | 0.166923 | 29.64069 | 4.47E-193 | 3.27E-190 |
| FABP6 | protein_coding | 1265.789 | 6.434703 | 0.219703 | 29.28815 | 1.47E-188 | 1.02E-185 |
| SAP30 | protein_coding | 1015.456 | 2.477157 | 0.084744 | 29.23118 | 7.79E-188 | 5.20E-185 |
| TREM2 | protein_coding | 1362.104 | 4.259559 | 0.146247 | 29.12572 | 1.70E-186 | 1.09E-183 |
| ATP1A1 | protein_coding | 71824.4 | -2.77783 | 0.095385 | -29.1224 | 1.87E-186 | 1.15E-183 |
| SIGLEC8 | protein_coding | 458.3112 | 5.065547 | 0.176225 | 28.74475 | 1.05E-181 | 5.99E-179 |
| DOC2A | protein_coding | 1798.231 | 6.140299 | 0.213948 | 28.7 | 3.81E-181 | 2.09E-178 |
| VEGFA | protein_coding | 91215.42 | 3.459717 | 0.120778 | 28.64536 | 1.83E-180 | 9.69E-178 |
| SEMA5B | protein_coding | 8847.175 | 3.890644 | 0.136188 | 28.56823 | 1.67E-179 | 8.53E-177 |
| NETO2 | protein_coding | 3894.699 | 3.288859 | 0.116977 | 28.11546 | 6.34E-174 | 3.04E-171 |
| TNFAIP6 | protein_coding | 2830.48 | 5.663579 | 0.201611 | 28.09164 | 1.24E-173 | 5.77E-171 |
| VIM | protein_coding | 169016.3 | 2.57569 | 0.092498 | 27.84604 | 1.20E-170 | 5.43E-168 |
| SLC16A3 | protein_coding | 15115.89 | 3.048926 | 0.109599 | 27.8188 | 2.57E-170 | 1.13E-167 |
| ST8SIA4 | protein_coding | 3248.126 | 3.519375 | 0.127001 | 27.71143 | 5.08E-169 | 2.11E-166 |
| APOC1 | protein_coding | 6010.432 | 5.22463 | 0.18989 | 27.51395 | 1.20E-166 | 4.83E-164 |
| ARHGEF39 | protein_coding | 338.3973 | 2.714323 | 0.099577 | 27.25857 | 1.32E-163 | 5.05E-161 |
| COL5A3 | protein_coding | 2202.836 | 3.545724 | 0.130606 | 27.14825 | 2.66E-162 | 9.94E-160 |
| NPTX2 | protein_coding | 10773.96 | 6.980847 | 0.258306 | 27.02555 | 7.41E-161 | 2.71E-158 |
| GRIK3 | protein_coding | 1585.935 | 5.005005 | 0.18575 | 26.94488 | 6.55E-160 | 2.34E-157 |
| MTURN | protein_coding | 3729.099 | -3.0822 | 0.114481 | -26.9233 | 1.17E-159 | 4.08E-157 |
| LILRB1 | protein_coding | 855.6507 | 2.921396 | 0.109546 | 26.66815 | 1.10E-156 | 3.76E-154 |
| HSPA2 | protein_coding | 3076.899 | -3.89118 | 0.146127 | -26.6288 | 3.15E-156 | 1.05E-153 |
| CD70 | protein_coding | 2982.74 | 6.1024 | 0.232094 | 26.29284 | 2.32E-152 | 7.11E-150 |
| ENPP3 | protein_coding | 15448.96 | 4.710454 | 0.179312 | 26.26965 | 4.26E-152 | 1.23E-149 |
| SLC6A3 | protein_coding | 9475.226 | 6.430264 | 0.245215 | 26.22292 | 1.46E-151 | 4.14E-149 |
| CXCR4 | protein_coding | 11122.62 | 2.782478 | 0.106872 | 26.03571 | 1.95E-149 | 5.08E-147 |
| PNCK | protein_coding | 6632.783 | 6.629622 | 0.25548 | 25.94969 | 1.83E-148 | 4.54E-146 |
| ARHGAP22 | protein_coding | 437.3079 | 2.758717 | 0.106395 | 25.92891 | 3.14E-148 | 7.66E-146 |
| HK2 | protein_coding | 5387.635 | 3.433499 | 0.132519 | 25.90943 | 5.21E-148 | 1.25E-145 |
| ODF3B | protein_coding | 1432.045 | 3.549096 | 0.137822 | 25.75128 | 3.12E-146 | 7.14E-144 |
| ZNF395 | protein_coding | 18557.56 | 2.621663 | 0.101859 | 25.73811 | 4.38E-146 | 9.89E-144 |
| P2RX7 | protein_coding | 1054.946 | 2.658521 | 0.103528 | 25.67935 | 1.99E-145 | 4.42E-143 |
| MYEOV | protein_coding | 1441.456 | 7.095918 | 0.277687 | 25.55369 | 5.00E-144 | 1.07E-141 |
| TMEM74B | protein_coding | 341.3636 | 3.731158 | 0.146034 | 25.54994 | 5.50E-144 | 1.16E-141 |
| HS6ST1 | protein_coding | 2801.375 | -2.15728 | 0.084446 | -25.5463 | 6.03E-144 | 1.25E-141 |
| AGAP2 | protein_coding | 399.3098 | 2.700193 | 0.1061 | 25.44947 | 7.16E-143 | 1.46E-140 |
| MTCP1 | protein_coding | 495.6124 | 3.255437 | 0.128001 | 25.43292 | 1.09E-142 | 2.20E-140 |
| HYKK | protein_coding | 210.3273 | -2.02708 | 0.079964 | -25.35 | 8.99E-142 | 1.77E-139 |
| STAMBPL1 | protein_coding | 829.5099 | 2.448632 | 0.096979 | 25.24915 | 1.16E-140 | 2.22E-138 |
| ENO2 | protein_coding | 10754.42 | 3.210219 | 0.127322 | 25.21344 | 2.85E-140 | 5.41E-138 |
| ESM1 | protein_coding | 14322.09 | 3.571863 | 0.142585 | 25.0507 | 1.72E-138 | 3.17E-136 |
| GSTM3 | protein_coding | 3417.968 | -3.31435 | 0.132785 | -24.9604 | 1.65E-137 | 3.01E-135 |
| SLC4A11 | protein_coding | 491.8621 | -4.47021 | 0.179219 | -24.9427 | 2.56E-137 | 4.63E-135 |
| CYP2J2 | protein_coding | 7959.638 | 5.112741 | 0.20506 | 24.93285 | 3.28E-137 | 5.85E-135 |
| EDA2R | protein_coding | 1003.009 | 2.278733 | 0.09179 | 24.82538 | 4.77E-136 | 8.32E-134 |
| ERMP1 | protein_coding | 4700.863 | -2.49458 | 0.100618 | -24.7926 | 1.08E-135 | 1.86E-133 |
| SDS | protein_coding | 777.3918 | 4.257685 | 0.171907 | 24.76731 | 2.02E-135 | 3.44E-133 |
| KRBA1 | protein_coding | 3538.47 | 2.797963 | 0.11302 | 24.75632 | 2.65E-135 | 4.47E-133 |
| NXPH4 | protein_coding | 1323.594 | 4.536993 | 0.183435 | 24.73359 | 4.66E-135 | 7.69E-133 |
| SOX11 | protein_coding | 332.3922 | 5.406009 | 0.219142 | 24.66898 | 2.30E-134 | 3.76E-132 |
| TMEM91 | protein_coding | 2580.591 | 3.752622 | 0.15214 | 24.66562 | 2.50E-134 | 4.04E-132 |
| INHBB | protein_coding | 4215.544 | 3.828957 | 0.155498 | 24.62377 | 7.03E-134 | 1.12E-131 |
| SLC4A8 | protein_coding | 214.8608 | -2.73763 | 0.1115 | -24.5529 | 4.03E-133 | 6.31E-131 |
| DOK3 | protein_coding | 792.4634 | 2.639956 | 0.107778 | 24.49441 | 1.69E-132 | 2.63E-130 |
| AHNAK2 | protein_coding | 7313.01 | 4.081021 | 0.166629 | 24.49168 | 1.81E-132 | 2.78E-130 |
| IGFBP3 | protein_coding | 134755.9 | 3.485026 | 0.142705 | 24.4212 | 1.02E-131 | 1.53E-129 |
| KISS1R | protein_coding | 529.0607 | 6.403766 | 0.262619 | 24.38425 | 2.51E-131 | 3.75E-129 |
| TNFRSF4 | protein_coding | 504.922 | 3.308483 | 0.136352 | 24.26433 | 4.67E-130 | 6.76E-128 |
| FGF1 | protein_coding | 1402.716 | -4.23153 | 0.174454 | -24.2559 | 5.72E-130 | 8.21E-128 |
| RASAL3 | protein_coding | 976.3313 | 2.674512 | 0.110544 | 24.19416 | 2.56E-129 | 3.64E-127 |
| PARVG | protein_coding | 1136.705 | 2.87326 | 0.119325 | 24.07934 | 4.12E-128 | 5.69E-126 |
| PIK3R5 | protein_coding | 992.7438 | 2.791184 | 0.115953 | 24.07158 | 4.96E-128 | 6.80E-126 |
| FAM78A | protein_coding | 862.3985 | 2.364991 | 0.098323 | 24.05318 | 7.73E-128 | 1.05E-125 |
| ITGAX | protein_coding | 1920.289 | 3.283995 | 0.136559 | 24.04816 | 8.73E-128 | 1.18E-125 |
| RAB42 | protein_coding | 1659.397 | 4.867178 | 0.202473 | 24.03868 | 1.10E-127 | 1.46E-125 |
| C14orf37 | protein_coding | 640.1274 | -3.59052 | 0.149459 | -24.0235 | 1.58E-127 | 2.09E-125 |
| HLA-F | protein_coding | 8554.127 | 2.451261 | 0.10216 | 23.99431 | 3.19E-127 | 4.18E-125 |
| TYMP | protein_coding | 5852.787 | 2.952834 | 0.123098 | 23.98771 | 3.74E-127 | 4.86E-125 |
| IDO1 | protein_coding | 2076.841 | 3.955886 | 0.164998 | 23.97541 | 5.02E-127 | 6.48E-125 |
| BHLHE41 | protein_coding | 7838.363 | 2.753596 | 0.115129 | 23.91746 | 2.02E-126 | 2.56E-124 |
| CD300A | protein_coding | 1234.454 | 2.966272 | 0.124102 | 23.9019 | 2.93E-126 | 3.68E-124 |
| PHKA2 | protein_coding | 7873.721 | 2.176694 | 0.091188 | 23.8703 | 6.23E-126 | 7.78E-124 |
| NNMT | protein_coding | 42202.19 | 3.925717 | 0.165065 | 23.78289 | 5.02E-125 | 6.22E-123 |
| C5orf46 | protein_coding | 903.7819 | 7.707455 | 0.3261 | 23.63527 | 1.67E-123 | 2.05E-121 |
| CD1D | protein_coding | 492.1218 | 2.269123 | 0.096013 | 23.6335 | 1.74E-123 | 2.13E-121 |
| GAL3ST4 | protein_coding | 977.6077 | 2.893345 | 0.122527 | 23.61388 | 2.78E-123 | 3.35E-121 |
| LAMA4 | protein_coding | 9806.196 | 2.646637 | 0.112435 | 23.53931 | 1.62E-122 | 1.92E-120 |
| AKAP3 | protein_coding | 97.60849 | -2.35211 | 0.099991 | -23.5232 | 2.36E-122 | 2.79E-120 |
| CD300LF | protein_coding | 295.5864 | 3.00629 | 0.128253 | 23.44033 | 1.66E-121 | 1.93E-119 |
| FCGR3A | protein_coding | 8079.101 | 3.324145 | 0.141852 | 23.43386 | 1.93E-121 | 2.23E-119 |
| GAS2L3 | protein_coding | 2284.901 | 3.085492 | 0.131874 | 23.39735 | 4.55E-121 | 5.21E-119 |
| PRDM1 | protein_coding | 2043.097 | 2.260448 | 0.096666 | 23.38413 | 6.20E-121 | 7.00E-119 |
| PLA2R1 | protein_coding | 1390.28 | -3.13319 | 0.134231 | -23.3417 | 1.67E-120 | 1.86E-118 |
| ACSF2 | protein_coding | 1941.954 | -3.28341 | 0.140676 | -23.3402 | 1.73E-120 | 1.91E-118 |
| ITGAD | protein_coding | 133.1151 | 4.774974 | 0.205554 | 23.22979 | 2.28E-119 | 2.46E-117 |
| PLXDC1 | protein_coding | 2814.845 | 2.874243 | 0.124061 | 23.16793 | 9.59E-119 | 1.02E-116 |
| FATE1 | protein_coding | 107.8948 | 3.534682 | 0.152825 | 23.12891 | 2.37E-118 | 2.51E-116 |
| COBLL1 | protein_coding | 6056.52 | -2.43038 | 0.105088 | -23.1271 | 2.47E-118 | 2.60E-116 |
| PTHLH | protein_coding | 2592.632 | 6.400987 | 0.277221 | 23.08984 | 5.86E-118 | 6.08E-116 |
| BIRC7 | protein_coding | 760.883 | 7.065552 | 0.306492 | 23.05296 | 1.37E-117 | 1.41E-115 |
| GJC1 | protein_coding | 2174.796 | 2.72176 | 0.118136 | 23.03919 | 1.89E-117 | 1.92E-115 |
| DGKD | protein_coding | 2722.344 | 2.102238 | 0.09127 | 23.03324 | 2.17E-117 | 2.17E-115 |
| WNK4 | protein_coding | 997.3411 | -4.07176 | 0.176776 | -23.0334 | 2.16E-117 | 2.17E-115 |
| TYMS | protein_coding | 1593.218 | 2.055801 | 0.089382 | 23.00015 | 4.64E-117 | 4.63E-115 |
| FABP7 | protein_coding | 11871.09 | 7.435569 | 0.3245 | 22.91391 | 3.38E-116 | 3.34E-114 |
| PGF | protein_coding | 9316.764 | 4.682529 | 0.20462 | 22.88399 | 6.71E-116 | 6.60E-114 |
| ADM | protein_coding | 11627.85 | 2.715771 | 0.11879 | 22.86187 | 1.11E-115 | 1.09E-113 |
| LCP2 | protein_coding | 2869.399 | 2.211262 | 0.096921 | 22.815 | 3.25E-115 | 3.16E-113 |
| TMIGD3 | protein_coding | 900.2298 | 2.912454 | 0.128245 | 22.71003 | 3.57E-114 | 3.40E-112 |
| IL2RB | protein_coding | 1525.285 | 3.007594 | 0.13255 | 22.69028 | 5.59E-114 | 5.26E-112 |
| EGF | protein_coding | 3720.785 | -5.48541 | 0.241886 | -22.6777 | 7.45E-114 | 6.97E-112 |
| ALDH6A1 | protein_coding | 6907.946 | -3.06775 | 0.13561 | -22.6219 | 2.64E-113 | 2.44E-111 |
| LPCAT1 | protein_coding | 14543.47 | 2.548347 | 0.11306 | 22.53972 | 1.69E-112 | 1.55E-110 |
| KLHL14 | protein_coding | 262.9523 | -3.86565 | 0.171909 | -22.4867 | 5.61E-112 | 5.06E-110 |
| MYO1F | protein_coding | 2086.11 | 2.502877 | 0.111415 | 22.46449 | 9.24E-112 | 8.29E-110 |
| TRPM2 | protein_coding | 838.3565 | 2.755898 | 0.122816 | 22.43915 | 1.63E-111 | 1.45E-109 |
| IL20RB | protein_coding | 1419.216 | 6.597319 | 0.294913 | 22.37035 | 7.65E-111 | 6.75E-109 |
| SLFN13 | protein_coding | 2422.938 | 2.150875 | 0.096201 | 22.35807 | 1.01E-110 | 8.84E-109 |
| PLK2 | protein_coding | 6275.086 | 2.216391 | 0.099493 | 22.27682 | 6.20E-110 | 5.35E-108 |
| SCD | protein_coding | 25597.06 | 3.009229 | 0.135233 | 22.25224 | 1.07E-109 | 9.20E-108 |
| ANGPT2 | protein_coding | 6780.802 | 3.111085 | 0.13992 | 22.23477 | 1.58E-109 | 1.35E-107 |
| VASH1 | protein_coding | 3070.99 | 2.163285 | 0.097425 | 22.20454 | 3.10E-109 | 2.63E-107 |
| DLX5 | protein_coding | 146.7123 | 3.440401 | 0.154981 | 22.19887 | 3.52E-109 | 2.97E-107 |
| TYROBP | protein_coding | 4690.81 | 2.67093 | 0.120358 | 22.19161 | 4.14E-109 | 3.45E-107 |
| EHD2 | protein_coding | 18965.11 | 2.254036 | 0.101622 | 22.18055 | 5.29E-109 | 4.39E-107 |
| LAPTM5 | protein_coding | 17653.25 | 2.541323 | 0.114862 | 22.125 | 1.82E-108 | 1.50E-106 |
| DNAH11 | protein_coding | 2067.327 | 4.52327 | 0.204491 | 22.11967 | 2.04E-108 | 1.68E-106 |
| FAM171A1 | protein_coding | 2471.682 | -2.17454 | 0.09832 | -22.1171 | 2.16E-108 | 1.77E-106 |
| RELT | protein_coding | 469.8369 | 2.084154 | 0.094383 | 22.08189 | 4.72E-108 | 3.79E-106 |
| IGSF6 | protein_coding | 1146.25 | 2.662618 | 0.120594 | 22.07917 | 5.01E-108 | 4.01E-106 |
| PSORS1C1 | protein_coding | 362.3143 | 2.966728 | 0.134479 | 22.06091 | 7.51E-108 | 5.94E-106 |
| PIK3R6 | protein_coding | 267.4607 | 3.385612 | 0.15374 | 22.02165 | 1.79E-107 | 1.39E-105 |
| PFKFB4 | protein_coding | 1631.32 | 2.419596 | 0.110081 | 21.98015 | 4.46E-107 | 3.44E-105 |
| LGALS9 | protein_coding | 2928.781 | 2.196058 | 0.100002 | 21.96019 | 6.92E-107 | 5.31E-105 |
| STAC3 | protein_coding | 241.0063 | 2.286947 | 0.10424 | 21.93919 | 1.10E-106 | 8.39E-105 |
| SRGAP3 | protein_coding | 485.9683 | -2.49924 | 0.114037 | -21.916 | 1.83E-106 | 1.39E-104 |
| MYO3A | protein_coding | 603.8333 | 3.274822 | 0.149546 | 21.89844 | 2.69E-106 | 2.03E-104 |
| PSMB9 | protein_coding | 4037.989 | 2.228483 | 0.102173 | 21.81088 | 1.83E-105 | 1.36E-103 |
| NLRC5 | protein_coding | 3107.838 | 2.230239 | 0.10237 | 21.78602 | 3.15E-105 | 2.32E-103 |
| LAIR1 | protein_coding | 2375.597 | 2.639293 | 0.121181 | 21.77974 | 3.61E-105 | 2.64E-103 |
| CSPG4 | protein_coding | 8384.342 | 2.729302 | 0.125402 | 21.76437 | 5.05E-105 | 3.67E-103 |
| POU5F1 | protein_coding | 1816.895 | 3.393491 | 0.155933 | 21.76244 | 5.27E-105 | 3.81E-103 |
| NHLRC4 | protein_coding | 172.6731 | -3.20646 | 0.147828 | -21.6906 | 2.52E-104 | 1.81E-102 |
| PLPPR5 | protein_coding | 394.4654 | 6.402033 | 0.295273 | 21.68176 | 3.05E-104 | 2.18E-102 |
| PRELID2 | protein_coding | 479.2479 | 2.002673 | 0.09247 | 21.65762 | 5.15E-104 | 3.66E-102 |
| CES4A | protein_coding | 1794.444 | 4.152519 | 0.191775 | 21.65304 | 5.69E-104 | 4.03E-102 |
| TNFRSF14 | protein_coding | 7627.73 | 2.137905 | 0.098924 | 21.6115 | 1.40E-103 | 9.81E-102 |
| DPEP2 | protein_coding | 329.1255 | 2.27585 | 0.10536 | 21.6008 | 1.77E-103 | 1.23E-101 |
| SIGLEC10 | protein_coding | 897.2132 | 2.974163 | 0.13783 | 21.57841 | 2.87E-103 | 1.99E-101 |
| LGI2 | protein_coding | 498.4963 | -3.28695 | 0.152385 | -21.5701 | 3.43E-103 | 2.35E-101 |
| TNFSF9 | protein_coding | 537.0315 | 3.481302 | 0.161449 | 21.56284 | 4.01E-103 | 2.74E-101 |
| SFXN2 | protein_coding | 1285.849 | -2.3885 | 0.110804 | -21.5561 | 4.64E-103 | 3.15E-101 |
| ADAMTS7 | protein_coding | 758.4677 | 2.443443 | 0.113379 | 21.55115 | 5.17E-103 | 3.49E-101 |
| VSIG1 | protein_coding | 222.6716 | 3.952474 | 0.183487 | 21.5409 | 6.44E-103 | 4.34E-101 |
| SLC37A2 | protein_coding | 1078.977 | 2.723577 | 0.126477 | 21.5341 | 7.46E-103 | 4.98E-101 |
| TMEM45B | protein_coding | 572.1332 | -3.94449 | 0.183563 | -21.4885 | 2.00E-102 | 1.33E-100 |
| CD68 | protein_coding | 233.1031 | 3.181142 | 0.148136 | 21.47446 | 2.70E-102 | 1.79E-100 |
| NUSAP1 | protein_coding | 1086.499 | 2.133772 | 0.099381 | 21.47053 | 2.94E-102 | 1.93E-100 |
| RNASET2 | protein_coding | 13735.36 | 3.360072 | 0.156557 | 21.46235 | 3.50E-102 | 2.30E-100 |
| PRKAR2B | protein_coding | 561.0734 | -2.25333 | 0.105072 | -21.4456 | 5.02E-102 | 3.26E-100 |
| LILRB4 | protein_coding | 1312.272 | 3.121713 | 0.145611 | 21.43875 | 5.82E-102 | 3.77E-100 |
| TCF21 | protein_coding | 540.132 | -3.53503 | 0.164929 | -21.4336 | 6.50E-102 | 4.19E-100 |
| SFRP1 | protein_coding | 3974.461 | -5.27503 | 0.24662 | -21.3893 | 1.68E-101 | 1.07E-99 |
| CYFIP2 | protein_coding | 16324.05 | -2.27471 | 0.106466 | -21.3656 | 2.79E-101 | 1.77E-99 |
| VWF | protein_coding | 61993.21 | 3.005919 | 0.140851 | 21.3411 | 4.72E-101 | 2.96E-99 |
| HS3ST2 | protein_coding | 353.0818 | 4.895711 | 0.229438 | 21.33786 | 5.06E-101 | 3.14E-99 |
| RASD2 | protein_coding | 820.3538 | 2.89389 | 0.136007 | 21.27747 | 1.84E-100 | 1.12E-98 |
| FCER1G | protein_coding | 2726.934 | 2.545136 | 0.11981 | 21.24318 | 3.81E-100 | 2.29E-98 |
| CACNA2D2 | protein_coding | 181.5031 | -2.29997 | 0.108482 | -21.2013 | 9.29E-100 | 5.48E-98 |
| NKG7 | protein_coding | 1652.001 | 3.496914 | 0.165059 | 21.18587 | 1.29E-99 | 7.55E-98 |
| FAM111B | protein_coding | 239.0885 | 2.513077 | 0.118897 | 21.13655 | 3.67E-99 | 2.13E-97 |
| CDK18 | protein_coding | 17423.12 | 2.368163 | 0.11208 | 21.1293 | 4.28E-99 | 2.46E-97 |
| PKMYT1 | protein_coding | 140.4214 | 2.955057 | 0.140011 | 21.10587 | 7.02E-99 | 4.01E-97 |
| CLIC5 | protein_coding | 2247.949 | -3.82701 | 0.181334 | -21.1048 | 7.19E-99 | 4.09E-97 |
| LAT2 | protein_coding | 933.2204 | 2.151181 | 0.102352 | 21.01752 | 4.54E-98 | 2.51E-96 |
| FAM153C | protein_coding | 237.5951 | 5.216687 | 0.248265 | 21.01255 | 5.04E-98 | 2.78E-96 |
| OSCAR | protein_coding | 282.889 | 2.59084 | 0.123336 | 21.00633 | 5.74E-98 | 3.16E-96 |
| C1QB | protein_coding | 14988.59 | 2.930103 | 0.139644 | 20.98266 | 9.45E-98 | 5.18E-96 |
| FCGR1A | protein_coding | 483.7732 | 3.102946 | 0.147934 | 20.97527 | 1.10E-97 | 6.01E-96 |
| CLEC2D | protein_coding | 858.5663 | 2.533416 | 0.120828 | 20.96712 | 1.31E-97 | 7.10E-96 |
| FMNL1 | protein_coding | 1972.144 | 2.244319 | 0.107161 | 20.94338 | 2.16E-97 | 1.16E-95 |
| LILRB2 | protein_coding | 832.7329 | 2.288248 | 0.109267 | 20.94188 | 2.23E-97 | 1.19E-95 |
| PYGL | protein_coding | 4095.206 | 2.009909 | 0.096114 | 20.9118 | 4.18E-97 | 2.23E-95 |
| GNLY | protein_coding | 694.2849 | 3.129324 | 0.149732 | 20.89951 | 5.41E-97 | 2.86E-95 |
| C1QTNF6 | protein_coding | 1278.225 | 2.293244 | 0.10991 | 20.86483 | 1.12E-96 | 5.84E-95 |
| LTB4R | protein_coding | 710.6455 | 2.53806 | 0.121688 | 20.85704 | 1.32E-96 | 6.82E-95 |
| PLA2G7 | protein_coding | 499.3094 | 3.659048 | 0.175492 | 20.85019 | 1.52E-96 | 7.85E-95 |
| PTPRN | protein_coding | 347.2619 | 5.353306 | 0.256812 | 20.84519 | 1.69E-96 | 8.68E-95 |
| MS4A14 | protein_coding | 229.6105 | 3.198577 | 0.153467 | 20.84215 | 1.80E-96 | 9.22E-95 |
| SPI1 | protein_coding | 1836.949 | 2.367318 | 0.113622 | 20.83513 | 2.08E-96 | 1.06E-94 |
| PROX1 | protein_coding | 549.134 | -4.35796 | 0.209527 | -20.7991 | 4.41E-96 | 2.24E-94 |
| OLFML2A | protein_coding | 8689.328 | 2.678725 | 0.128989 | 20.76711 | 8.59E-96 | 4.34E-94 |
| SASH3 | protein_coding | 1418.596 | 2.477092 | 0.11935 | 20.75491 | 1.11E-95 | 5.55E-94 |
| IL12RB1 | protein_coding | 386.7544 | 2.591259 | 0.124959 | 20.73692 | 1.61E-95 | 8.05E-94 |
| CAV1 | protein_coding | 14772.33 | 2.233599 | 0.107814 | 20.71715 | 2.43E-95 | 1.21E-93 |
| KLHL6 | protein_coding | 743.8944 | 2.589449 | 0.125027 | 20.71117 | 2.75E-95 | 1.36E-93 |
| EFHD1 | protein_coding | 4585.211 | -2.62628 | 0.127069 | -20.6681 | 6.71E-95 | 3.32E-93 |
| KLRD1 | protein_coding | 386.8638 | 2.291453 | 0.110911 | 20.6602 | 7.90E-95 | 3.90E-93 |
| ABAT | protein_coding | 2746.485 | -3.05951 | 0.148122 | -20.6554 | 8.73E-95 | 4.29E-93 |
| HLX | protein_coding | 1314.379 | 2.551716 | 0.12354 | 20.65503 | 8.80E-95 | 4.31E-93 |
| APOBEC3G | protein_coding | 1624.749 | 2.330169 | 0.112828 | 20.65246 | 9.28E-95 | 4.54E-93 |
| CCND1 | protein_coding | 45420.48 | 2.287628 | 0.110791 | 20.64821 | 1.01E-94 | 4.94E-93 |
| CSF3R | protein_coding | 1090.964 | 2.745833 | 0.133217 | 20.61167 | 2.16E-94 | 1.05E-92 |
| SLC16A5 | protein_coding | 873.5135 | -3.0523 | 0.1482 | -20.5958 | 2.99E-94 | 1.45E-92 |
| CDC45 | protein_coding | 146.9553 | 2.729914 | 0.132548 | 20.59561 | 3.00E-94 | 1.45E-92 |
| C3 | protein_coding | 103265.8 | 3.736819 | 0.181871 | 20.54657 | 8.26E-94 | 3.96E-92 |
| P2RY1 | protein_coding | 840.7488 | 2.405736 | 0.117184 | 20.52961 | 1.17E-93 | 5.60E-92 |
| ETV7 | protein_coding | 519.1173 | 2.680071 | 0.130663 | 20.51138 | 1.70E-93 | 8.12E-92 |
| RUNX3 | protein_coding | 1392.983 | 2.681886 | 0.130845 | 20.49666 | 2.31E-93 | 1.10E-91 |
| LY86 | protein_coding | 709.6767 | 2.483496 | 0.121284 | 20.47664 | 3.48E-93 | 1.63E-91 |
| TRIM9 | protein_coding | 931.4849 | 2.842606 | 0.138898 | 20.46549 | 4.37E-93 | 2.05E-91 |
| KCNK9 | protein_coding | 252.0757 | 4.555218 | 0.223003 | 20.42674 | 9.68E-93 | 4.50E-91 |
| ATP8B3 | protein_coding | 644.921 | 4.241197 | 0.20767 | 20.42276 | 1.05E-92 | 4.87E-91 |
| GOLGA7B | protein_coding | 280.7361 | 3.865997 | 0.189476 | 20.40366 | 1.55E-92 | 7.13E-91 |
| KCNK13 | protein_coding | 196.2458 | -2.63741 | 0.129285 | -20.4 | 1.67E-92 | 7.66E-91 |
| CCDC88B | protein_coding | 1004.851 | 2.588404 | 0.126963 | 20.387 | 2.18E-92 | 9.94E-91 |
| C1orf162 | protein_coding | 1739.913 | 2.345089 | 0.115076 | 20.37853 | 2.59E-92 | 1.18E-90 |
| MECOM | protein_coding | 3642.737 | -2.59953 | 0.127591 | -20.374 | 2.85E-92 | 1.29E-90 |
| C4orf47 | protein_coding | 377.3719 | 2.518821 | 0.123664 | 20.36825 | 3.20E-92 | 1.44E-90 |
| C1QC | protein_coding | 12166.46 | 2.755211 | 0.135279 | 20.36682 | 3.29E-92 | 1.48E-90 |
| TRPA1 | protein_coding | 375.7577 | 3.731028 | 0.183353 | 20.34891 | 4.75E-92 | 2.13E-90 |
| RGS1 | protein_coding | 6804.087 | 3.081647 | 0.151464 | 20.34568 | 5.07E-92 | 2.26E-90 |
| RASSF2 | protein_coding | 3017.16 | 2.177666 | 0.107054 | 20.34169 | 5.50E-92 | 2.45E-90 |
| FAM163A | protein_coding | 84.68258 | 3.223704 | 0.158948 | 20.28145 | 1.88E-91 | 8.27E-90 |
| FOXC1 | protein_coding | 1498.005 | -2.17979 | 0.107489 | -20.2792 | 1.96E-91 | 8.63E-90 |
| ISG20 | protein_coding | 839.0327 | 2.365293 | 0.116693 | 20.26933 | 2.40E-91 | 1.05E-89 |
| PRF1 | protein_coding | 1413.295 | 2.636431 | 0.130141 | 20.25826 | 3.00E-91 | 1.31E-89 |
| APOBEC3H | protein_coding | 105.4066 | 2.91728 | 0.144254 | 20.22315 | 6.12E-91 | 2.65E-89 |
| SEMA6D | protein_coding | 1107.885 | -2.95322 | 0.146032 | -20.2231 | 6.14E-91 | 2.65E-89 |
| PRDM16 | protein_coding | 486.8335 | -3.73893 | 0.184921 | -20.2191 | 6.65E-91 | 2.86E-89 |
| BTBD16 | protein_coding | 196.7834 | 3.580925 | 0.177194 | 20.20909 | 8.14E-91 | 3.48E-89 |
| ITGB2 | protein_coding | 8626.503 | 2.389477 | 0.118276 | 20.20253 | 9.30E-91 | 3.97E-89 |
| LZTS1 | protein_coding | 2469.995 | 2.388414 | 0.118367 | 20.17812 | 1.52E-90 | 6.45E-89 |
| CPAMD8 | protein_coding | 850.1449 | -2.79523 | 0.138527 | -20.1782 | 1.52E-90 | 6.45E-89 |
| TMSB10 | protein_coding | 70055.47 | 2.146196 | 0.106415 | 20.16823 | 1.86E-90 | 7.83E-89 |
| PTPRO | protein_coding | 1155.758 | -3.21666 | 0.159605 | -20.1539 | 2.48E-90 | 1.04E-88 |
| PADI1 | protein_coding | 508.6418 | 5.718243 | 0.284088 | 20.12839 | 4.16E-90 | 1.73E-88 |
| LOX | protein_coding | 18846.13 | 4.051386 | 0.201564 | 20.09973 | 7.42E-90 | 3.06E-88 |
| FCGR1B | protein_coding | 89.50679 | 2.854042 | 0.142026 | 20.0952 | 8.13E-90 | 3.35E-88 |
| MS4A7 | protein_coding | 3397.52 | 2.382116 | 0.118565 | 20.09129 | 8.80E-90 | 3.61E-88 |
| NFAM1 | protein_coding | 892.2392 | 2.114024 | 0.105235 | 20.0886 | 9.28E-90 | 3.80E-88 |
| ABHD17C | protein_coding | 669.0167 | -2.05305 | 0.102221 | -20.0844 | 1.01E-89 | 4.13E-88 |
| ARHGAP9 | protein_coding | 794.7358 | 2.554342 | 0.127185 | 20.08372 | 1.02E-89 | 4.17E-88 |
| CD247 | protein_coding | 642.5737 | 2.581857 | 0.128632 | 20.07161 | 1.31E-89 | 5.31E-88 |
| GZMH | protein_coding | 396.4257 | 2.99569 | 0.149487 | 20.0398 | 2.48E-89 | 1.00E-87 |
| MCHR1 | protein_coding | 704.5981 | 5.733242 | 0.286169 | 20.03448 | 2.76E-89 | 1.11E-87 |
| OLFML2B | protein_coding | 3707.308 | 2.691497 | 0.134416 | 20.02361 | 3.43E-89 | 1.38E-87 |
| PPP1R3G | protein_coding | 522.0365 | 2.139141 | 0.106898 | 20.01104 | 4.41E-89 | 1.76E-87 |
| PLCL1 | protein_coding | 1983.676 | -2.65751 | 0.132952 | -19.9885 | 6.93E-89 | 2.76E-87 |
| CCL18 | protein_coding | 906.0648 | 5.695403 | 0.285265 | 19.96531 | 1.10E-88 | 4.38E-87 |
| FGD2 | protein_coding | 811.5046 | 2.4808 | 0.124287 | 19.96024 | 1.22E-88 | 4.83E-87 |
| SIGLEC9 | protein_coding | 284.8605 | 2.304621 | 0.115492 | 19.95478 | 1.36E-88 | 5.38E-87 |
| TMC8 | protein_coding | 1082.403 | 2.526066 | 0.126751 | 19.92931 | 2.27E-88 | 8.92E-87 |
| BIN2 | protein_coding | 951.7836 | 2.010878 | 0.10095 | 19.9196 | 2.75E-88 | 1.08E-86 |
| FASLG | protein_coding | 156.0478 | 3.486608 | 0.175249 | 19.89522 | 4.48E-88 | 1.75E-86 |
| YPEL4 | protein_coding | 139.7855 | 2.654458 | 0.13344 | 19.89255 | 4.72E-88 | 1.84E-86 |
| LILRB3 | protein_coding | 208.4657 | 2.465643 | 0.123972 | 19.88864 | 5.10E-88 | 1.99E-86 |
| UHRF1 | protein_coding | 266.3203 | 2.768479 | 0.139338 | 19.86877 | 7.58E-88 | 2.95E-86 |
| TROAP | protein_coding | 178.9719 | 3.264418 | 0.164433 | 19.8526 | 1.05E-87 | 4.05E-86 |
| ACADSB | protein_coding | 3995.161 | -2.03438 | 0.102486 | -19.8504 | 1.09E-87 | 4.22E-86 |
| PLIN2 | protein_coding | 54741.46 | 2.905169 | 0.146708 | 19.80239 | 2.84E-87 | 1.08E-85 |
| EMX1 | protein_coding | 1197.23 | -2.61485 | 0.132164 | -19.785 | 4.01E-87 | 1.51E-85 |
| PRAM1 | protein_coding | 254.6344 | 2.465085 | 0.124625 | 19.78001 | 4.43E-87 | 1.66E-85 |
| CCL5 | protein_coding | 4139.518 | 3.240949 | 0.163866 | 19.778 | 4.61E-87 | 1.72E-85 |
| C6orf223 | protein_coding | 2940.541 | 3.487758 | 0.176443 | 19.76705 | 5.72E-87 | 2.12E-85 |
| CD72 | protein_coding | 424.999 | 2.724151 | 0.13785 | 19.76165 | 6.37E-87 | 2.36E-85 |
| CRHBP | protein_coding | 403.1042 | -4.08185 | 0.207008 | -19.7183 | 1.50E-86 | 5.54E-85 |
| RNF150 | protein_coding | 1192.522 | -3.15929 | 0.160417 | -19.6943 | 2.41E-86 | 8.87E-85 |
| AIF1L | protein_coding | 15382.76 | -3.22258 | 0.163699 | -19.6861 | 2.84E-86 | 1.04E-84 |
| FAM81A | protein_coding | 317.6732 | -2.55303 | 0.12979 | -19.6704 | 3.87E-86 | 1.41E-84 |
| E2F1 | protein_coding | 365.1696 | 2.195773 | 0.111721 | 19.65414 | 5.33E-86 | 1.93E-84 |
| ALOX15B | protein_coding | 241.9208 | 4.296302 | 0.218637 | 19.65041 | 5.74E-86 | 2.08E-84 |
| TBX21 | protein_coding | 139.1633 | 2.470705 | 0.125924 | 19.62062 | 1.03E-85 | 3.72E-84 |
| GAL3ST1 | protein_coding | 8633.383 | 2.94448 | 0.150124 | 19.6137 | 1.18E-85 | 4.25E-84 |
| TMEM178A | protein_coding | 340.4931 | -3.22514 | 0.164436 | -19.6133 | 1.19E-85 | 4.27E-84 |
| SLC16A1 | protein_coding | 5140.289 | 2.034949 | 0.103774 | 19.60935 | 1.29E-85 | 4.59E-84 |
| GZMA | protein_coding | 1016.73 | 3.13224 | 0.159817 | 19.59898 | 1.58E-85 | 5.62E-84 |
| EYA4 | protein_coding | 276.5432 | -3.51273 | 0.179324 | -19.5888 | 1.93E-85 | 6.85E-84 |
| LST1 | protein_coding | 1052.137 | 2.26313 | 0.115568 | 19.58259 | 2.18E-85 | 7.70E-84 |
| SLA2 | protein_coding | 304.6803 | 2.914365 | 0.148832 | 19.58151 | 2.22E-85 | 7.85E-84 |
| SLC7A8 | protein_coding | 4042.369 | -3.58155 | 0.183243 | -19.5454 | 4.52E-85 | 1.58E-83 |
| PCK2 | protein_coding | 3986.238 | -2.29998 | 0.117708 | -19.5398 | 5.04E-85 | 1.76E-83 |
| VAV1 | protein_coding | 799.024 | 2.302776 | 0.117933 | 19.52609 | 6.59E-85 | 2.29E-83 |
| SCN1B | protein_coding | 1068.742 | 2.000676 | 0.102471 | 19.52436 | 6.82E-85 | 2.37E-83 |
| BTG2 | protein_coding | 10043.43 | -2.10479 | 0.107874 | -19.5116 | 8.75E-85 | 3.02E-83 |
| TNFRSF9 | protein_coding | 512.1865 | 4.051209 | 0.207694 | 19.50567 | 9.83E-85 | 3.38E-83 |
| DLL4 | protein_coding | 5163.822 | 2.429233 | 0.124773 | 19.46916 | 2.01E-84 | 6.89E-83 |
| LGI4 | protein_coding | 5212.231 | 4.590403 | 0.235864 | 19.46207 | 2.30E-84 | 7.89E-83 |
| GBP5 | protein_coding | 1921.821 | 3.485538 | 0.179161 | 19.4548 | 2.65E-84 | 9.07E-83 |
| CP | protein_coding | 25497.37 | 4.569824 | 0.234985 | 19.44728 | 3.07E-84 | 1.05E-82 |
| SLC15A2 | protein_coding | 494.9369 | -3.10606 | 0.15989 | -19.4262 | 4.64E-84 | 1.58E-82 |
| ASS1 | protein_coding | 14122.11 | -2.7193 | 0.140002 | -19.4232 | 4.91E-84 | 1.67E-82 |
| CAPN12 | protein_coding | 1610.218 | 3.15245 | 0.162434 | 19.40754 | 6.66E-84 | 2.25E-82 |
| MYO1G | protein_coding | 991.4495 | 2.640426 | 0.136086 | 19.40257 | 7.34E-84 | 2.47E-82 |
| FKBP10 | protein_coding | 11894.27 | 2.807022 | 0.144753 | 19.39181 | 9.05E-84 | 3.03E-82 |
| CORO1A | protein_coding | 3784.952 | 2.43657 | 0.125669 | 19.38881 | 9.59E-84 | 3.21E-82 |
| FERMT3 | protein_coding | 1720.422 | 2.110788 | 0.108909 | 19.38116 | 1.11E-83 | 3.71E-82 |
| PATL2 | protein_coding | 166.774 | 2.814009 | 0.145232 | 19.37593 | 1.23E-83 | 4.09E-82 |
| BARX2 | protein_coding | 2579.158 | 2.636198 | 0.136283 | 19.34358 | 2.31E-83 | 7.64E-82 |
| ZAP70 | protein_coding | 486.7556 | 2.896519 | 0.149742 | 19.34339 | 2.32E-83 | 7.65E-82 |
| HK3 | protein_coding | 316.0636 | 2.593752 | 0.134155 | 19.33395 | 2.78E-83 | 9.15E-82 |
| CASZ1 | protein_coding | 396.7367 | -2.06003 | 0.10658 | -19.3286 | 3.09E-83 | 1.01E-81 |
| KIF18B | protein_coding | 170.5164 | 3.242843 | 0.167841 | 19.32093 | 3.58E-83 | 1.17E-81 |
| C10orf10 | protein_coding | 20573.99 | 2.263615 | 0.117186 | 19.31641 | 3.91E-83 | 1.28E-81 |
| CTSW | protein_coding | 815.7404 | 3.033864 | 0.157169 | 19.30317 | 5.05E-83 | 1.65E-81 |
| KCNN1 | protein_coding | 197.5176 | 4.305462 | 0.223066 | 19.3013 | 5.24E-83 | 1.70E-81 |
| BTK | protein_coding | 598.2375 | 2.17426 | 0.112655 | 19.30016 | 5.35E-83 | 1.74E-81 |
| XPNPEP2 | protein_coding | 1839.791 | -5.38358 | 0.278973 | -19.2978 | 5.60E-83 | 1.81E-81 |
| MCAM | protein_coding | 24735.86 | 2.070669 | 0.107369 | 19.28551 | 7.11E-83 | 2.28E-81 |
| TNFAIP8L2 | protein_coding | 401.0406 | 2.262008 | 0.117294 | 19.28491 | 7.19E-83 | 2.30E-81 |
| EFNA3 | protein_coding | 266.3718 | 2.419541 | 0.125608 | 19.26259 | 1.11E-82 | 3.53E-81 |
| EOMES | protein_coding | 387.7496 | 3.495681 | 0.181583 | 19.25115 | 1.38E-82 | 4.38E-81 |
| PNMA2 | protein_coding | 4411.708 | 2.822274 | 0.146919 | 19.20968 | 3.07E-82 | 9.70E-81 |
| CST7 | protein_coding | 909.5617 | 3.126325 | 0.163105 | 19.16752 | 6.91E-82 | 2.17E-80 |
| PRSS53 | protein_coding | 182.6968 | 3.521397 | 0.183844 | 19.15424 | 8.92E-82 | 2.79E-80 |
| C1QA | protein_coding | 12749.93 | 2.618176 | 0.136708 | 19.15157 | 9.39E-82 | 2.94E-80 |
| TRIB3 | protein_coding | 3404.899 | 3.145972 | 0.164664 | 19.10539 | 2.28E-81 | 7.09E-80 |
| ITGAL | protein_coding | 2400.98 | 2.517986 | 0.131975 | 19.07931 | 3.75E-81 | 1.16E-79 |
| APOBEC3C | protein_coding | 3696.826 | 2.109793 | 0.110758 | 19.04866 | 6.74E-81 | 2.08E-79 |
| HLA-G | protein_coding | 2240.327 | 3.852891 | 0.202514 | 19.02531 | 1.05E-80 | 3.24E-79 |
| MUC3A | protein_coding | 1266.737 | 3.429516 | 0.180287 | 19.02253 | 1.11E-80 | 3.41E-79 |
| KCNE3 | protein_coding | 4826.119 | 2.117989 | 0.111496 | 18.99602 | 1.84E-80 | 5.60E-79 |
| PPP2R2B | protein_coding | 217.2508 | -2.29688 | 0.120923 | -18.9946 | 1.89E-80 | 5.75E-79 |
| CAVIN3 | protein_coding | 2889.409 | 2.316155 | 0.122003 | 18.98438 | 2.30E-80 | 6.94E-79 |
| TMEM52B | protein_coding | 2016.025 | -5.04675 | 0.266015 | -18.9717 | 2.92E-80 | 8.77E-79 |
| DEF6 | protein_coding | 777.4163 | 2.275687 | 0.119953 | 18.97154 | 2.93E-80 | 8.77E-79 |
| MYBL2 | protein_coding | 386.2329 | 3.426507 | 0.180666 | 18.96593 | 3.26E-80 | 9.74E-79 |
| HCK | protein_coding | 1397.788 | 2.042964 | 0.107893 | 18.93515 | 5.86E-80 | 1.74E-78 |
| MRO | protein_coding | 531.0516 | -2.99865 | 0.158403 | -18.9305 | 6.40E-80 | 1.88E-78 |
| CD8A | protein_coding | 2097.498 | 3.593549 | 0.18985 | 18.92832 | 6.66E-80 | 1.96E-78 |
| SELPLG | protein_coding | 1744.071 | 2.05652 | 0.108705 | 18.91837 | 8.05E-80 | 2.36E-78 |
| THEMIS2 | protein_coding | 2145.17 | 2.056278 | 0.10882 | 18.89621 | 1.23E-79 | 3.56E-78 |
| FAM26F | protein_coding | 753.2559 | 2.965222 | 0.15717 | 18.86638 | 2.16E-79 | 6.23E-78 |
| APLN | protein_coding | 5741.596 | 2.464547 | 0.130684 | 18.85883 | 2.49E-79 | 7.17E-78 |
| CGREF1 | protein_coding | 1653.639 | 3.044393 | 0.161651 | 18.83316 | 4.04E-79 | 1.16E-77 |
| ACY1 | protein_coding | 548.883 | -2.19025 | 0.116379 | -18.82 | 5.18E-79 | 1.48E-77 |
| CD37 | protein_coding | 1609.393 | 2.146644 | 0.114089 | 18.81551 | 5.64E-79 | 1.61E-77 |
| MCM10 | protein_coding | 114.068 | 2.516615 | 0.133776 | 18.81215 | 6.00E-79 | 1.71E-77 |
| PSTPIP1 | protein_coding | 434.788 | 2.783569 | 0.148128 | 18.79159 | 8.85E-79 | 2.52E-77 |
| NKAIN1 | protein_coding | 145.9061 | 4.037107 | 0.214917 | 18.78449 | 1.01E-78 | 2.87E-77 |
| WAS | protein_coding | 1024.194 | 2.186312 | 0.116657 | 18.74129 | 2.28E-78 | 6.45E-77 |
| PYHIN1 | protein_coding | 246.6698 | 2.952536 | 0.157637 | 18.72998 | 2.82E-78 | 7.96E-77 |
| AURKB | protein_coding | 176.3138 | 3.050932 | 0.162901 | 18.72877 | 2.88E-78 | 8.13E-77 |
| JAK3 | protein_coding | 1790.576 | 2.438205 | 0.130322 | 18.70907 | 4.18E-78 | 1.17E-76 |
| FAM167A | protein_coding | 403.4678 | -3.53779 | 0.189166 | -18.702 | 4.77E-78 | 1.33E-76 |
| SLC1A3 | protein_coding | 1246.253 | 2.697731 | 0.144381 | 18.68478 | 6.58E-78 | 1.83E-76 |
| ZNF683 | protein_coding | 152.7644 | 3.398107 | 0.181869 | 18.6844 | 6.63E-78 | 1.84E-76 |
| TMEM45A | protein_coding | 2588.222 | 3.499047 | 0.187309 | 18.68062 | 7.12E-78 | 1.98E-76 |
| S1PR5 | protein_coding | 236.6786 | 2.407997 | 0.128925 | 18.67756 | 7.54E-78 | 2.09E-76 |
| APOBEC3D | protein_coding | 399.4415 | 2.02094 | 0.108211 | 18.67593 | 7.77E-78 | 2.15E-76 |
| BATF | protein_coding | 200.3798 | 3.032986 | 0.162437 | 18.67173 | 8.41E-78 | 2.32E-76 |
| CD2 | protein_coding | 1307.753 | 3.016333 | 0.161667 | 18.6577 | 1.09E-77 | 3.01E-76 |
| LILRA6 | protein_coding | 126.7742 | 2.518486 | 0.135157 | 18.63383 | 1.71E-77 | 4.69E-76 |
| CD84 | protein_coding | 1774.433 | 2.748712 | 0.147514 | 18.63356 | 1.72E-77 | 4.71E-76 |
| APBB1IP | protein_coding | 3216.956 | 2.424388 | 0.130191 | 18.62176 | 2.14E-77 | 5.85E-76 |
| CRACR2A | protein_coding | 255.3792 | 2.134902 | 0.114674 | 18.61707 | 2.34E-77 | 6.37E-76 |
| TNFSF14 | protein_coding | 398.7086 | 3.797271 | 0.203984 | 18.61554 | 2.40E-77 | 6.54E-76 |
| CDH8 | protein_coding | 119.7356 | 3.431429 | 0.184556 | 18.59289 | 3.67E-77 | 9.90E-76 |
| CAPN11 | protein_coding | 122.4251 | 3.350111 | 0.180192 | 18.59184 | 3.74E-77 | 1.01E-75 |
| HSPB8 | protein_coding | 13354.74 | 2.166781 | 0.11658 | 18.58629 | 4.15E-77 | 1.12E-75 |
| MXD3 | protein_coding | 273.0773 | 2.569018 | 0.138265 | 18.58033 | 4.64E-77 | 1.24E-75 |
| DIRAS2 | protein_coding | 1628.006 | 2.978562 | 0.160446 | 18.56422 | 6.26E-77 | 1.67E-75 |
| AVPR1B | protein_coding | 206.7873 | 3.720177 | 0.200536 | 18.55118 | 7.98E-77 | 2.13E-75 |
| DDIT4 | protein_coding | 15827.32 | 2.171791 | 0.117123 | 18.54282 | 9.32E-77 | 2.48E-75 |
| CXCL9 | protein_coding | 4564.128 | 3.58546 | 0.193367 | 18.54229 | 9.41E-77 | 2.50E-75 |
| CCR5 | protein_coding | 1111.94 | 2.88232 | 0.155535 | 18.53168 | 1.15E-76 | 3.04E-75 |
| CLEC2B | protein_coding | 1077.806 | 2.312209 | 0.124837 | 18.52186 | 1.38E-76 | 3.64E-75 |
| CXCL10 | protein_coding | 2066.415 | 3.362656 | 0.181808 | 18.49567 | 2.24E-76 | 5.90E-75 |
| ALB | protein_coding | 1723.786 | -5.06804 | 0.274041 | -18.4937 | 2.32E-76 | 6.11E-75 |
| MTCL1 | protein_coding | 2084.42 | 2.236261 | 0.120958 | 18.48796 | 2.58E-76 | 6.79E-75 |
| KIF21B | protein_coding | 514.3359 | 2.112098 | 0.114364 | 18.46826 | 3.72E-76 | 9.74E-75 |
| CHSY3 | protein_coding | 314.5253 | 2.204987 | 0.119409 | 18.46585 | 3.89E-76 | 1.02E-74 |
| ACKR3 | protein_coding | 5098.11 | 2.293625 | 0.124229 | 18.46286 | 4.11E-76 | 1.07E-74 |
| STRA8 | protein_coding | 146.0484 | 3.308017 | 0.179214 | 18.45851 | 4.45E-76 | 1.16E-74 |
| MS4A6A | protein_coding | 4467.908 | 2.232966 | 0.121021 | 18.45107 | 5.11E-76 | 1.33E-74 |
| CCNA2 | protein_coding | 353.4753 | 2.245704 | 0.121746 | 18.44586 | 5.63E-76 | 1.46E-74 |
| TYRO3 | protein_coding | 725.3995 | -2.2399 | 0.121502 | -18.4351 | 6.87E-76 | 1.78E-74 |
| KLHL13 | protein_coding | 972.871 | -2.17824 | 0.118408 | -18.3962 | 1.41E-75 | 3.61E-74 |
| TBC1D10C | protein_coding | 615.492 | 2.541538 | 0.138155 | 18.39625 | 1.41E-75 | 3.61E-74 |
| CLEC7A | protein_coding | 824.2253 | 2.320005 | 0.126717 | 18.30855 | 7.07E-75 | 1.78E-73 |
| C1orf226 | protein_coding | 367.8461 | -2.77293 | 0.151616 | -18.2892 | 1.01E-74 | 2.53E-73 |
| SIRPB2 | protein_coding | 349.8875 | 2.119521 | 0.115926 | 18.28345 | 1.12E-74 | 2.80E-73 |
| CDT1 | protein_coding | 326.6019 | 2.287171 | 0.125352 | 18.24596 | 2.23E-74 | 5.52E-73 |
| NCKAP1L | protein_coding | 2899.076 | 2.291016 | 0.125594 | 18.24145 | 2.42E-74 | 5.97E-73 |
| UNC13D | protein_coding | 839.4976 | 2.1803 | 0.119695 | 18.21546 | 3.89E-74 | 9.56E-73 |
| KIF14 | protein_coding | 117.4927 | 2.726921 | 0.14971 | 18.21467 | 3.95E-74 | 9.68E-73 |
| ARHGAP30 | protein_coding | 2084.168 | 2.018135 | 0.110806 | 18.21315 | 4.06E-74 | 9.92E-73 |
| ASF1B | protein_coding | 390.3073 | 2.171022 | 0.119292 | 18.19921 | 5.24E-74 | 1.28E-72 |
| CLEC12A | protein_coding | 234.0863 | 2.786477 | 0.153274 | 18.17967 | 7.48E-74 | 1.82E-72 |
| SLC17A9 | protein_coding | 605.4883 | 3.361298 | 0.185067 | 18.16256 | 1.02E-73 | 2.47E-72 |
| NIPAL1 | protein_coding | 346.8158 | -3.25464 | 0.179375 | -18.1443 | 1.42E-73 | 3.44E-72 |
| SAMD3 | protein_coding | 149.7511 | 2.299911 | 0.126958 | 18.1155 | 2.40E-73 | 5.77E-72 |
| RAB7B | protein_coding | 346.2697 | 2.062257 | 0.113851 | 18.1136 | 2.49E-73 | 5.96E-72 |
| EBF2 | protein_coding | 756.1935 | 2.696409 | 0.148872 | 18.11221 | 2.55E-73 | 6.10E-72 |
| PAPPA | protein_coding | 665.0333 | -3.19381 | 0.176352 | -18.1104 | 2.64E-73 | 6.28E-72 |
| E2F2 | protein_coding | 117.0041 | 2.360784 | 0.130395 | 18.10487 | 2.92E-73 | 6.92E-72 |
| SLCO1C1 | protein_coding | 101.736 | 2.497458 | 0.137944 | 18.10493 | 2.91E-73 | 6.92E-72 |
| IL10RA | protein_coding | 2887.524 | 2.22394 | 0.122869 | 18.10007 | 3.18E-73 | 7.53E-72 |
| HLA-DPB1 | protein_coding | 27440.28 | 2.064159 | 0.114108 | 18.08954 | 3.85E-73 | 9.06E-72 |
| C11orf21 | protein_coding | 127.2615 | 2.638883 | 0.1459 | 18.08692 | 4.04E-73 | 9.48E-72 |
| CD244 | protein_coding | 159.5348 | 2.30333 | 0.127353 | 18.08624 | 4.09E-73 | 9.59E-72 |
| LSP1 | protein_coding | 2935.216 | 2.258611 | 0.125117 | 18.05206 | 7.60E-73 | 1.76E-71 |
| FCRL6 | protein_coding | 155.5196 | 2.491511 | 0.138085 | 18.0433 | 8.91E-73 | 2.06E-71 |
| EPCAM | protein_coding | 5166.514 | -2.86661 | 0.158888 | -18.0417 | 9.17E-73 | 2.12E-71 |
| LTB4R2 | protein_coding | 131.7482 | 2.184261 | 0.121214 | 18.0199 | 1.36E-72 | 3.12E-71 |
| SCGN | protein_coding | 2230.765 | 4.676132 | 0.259528 | 18.01783 | 1.41E-72 | 3.23E-71 |
| HJURP | protein_coding | 209.9925 | 2.904663 | 0.161215 | 18.01736 | 1.42E-72 | 3.26E-71 |
| MPP7 | protein_coding | 894.4308 | -2.83066 | 0.157282 | -17.9974 | 2.04E-72 | 4.65E-71 |
| SORD | protein_coding | 1629.804 | -2.19104 | 0.121842 | -17.9827 | 2.66E-72 | 6.04E-71 |
| CRTAM | protein_coding | 162.6218 | 3.031426 | 0.168656 | 17.974 | 3.11E-72 | 7.05E-71 |
| C9orf172 | protein_coding | 425.122 | 2.071143 | 0.11527 | 17.96779 | 3.48E-72 | 7.87E-71 |
| DLGAP5 | protein_coding | 190.3199 | 2.732098 | 0.152092 | 17.9634 | 3.77E-72 | 8.51E-71 |
| MSC | protein_coding | 2261.868 | 3.10284 | 0.17275 | 17.96143 | 3.91E-72 | 8.81E-71 |
| CCSER1 | protein_coding | 187.4893 | -2.61822 | 0.145888 | -17.9468 | 5.09E-72 | 1.14E-70 |
| GDF6 | protein_coding | 861.3487 | 3.517346 | 0.196009 | 17.94487 | 5.26E-72 | 1.18E-70 |
| PTH1R | protein_coding | 2643.502 | -3.25233 | 0.18138 | -17.931 | 6.75E-72 | 1.51E-70 |
| CXCL11 | protein_coding | 481.5861 | 3.470435 | 0.193562 | 17.92931 | 6.96E-72 | 1.56E-70 |
| PCLAF | protein_coding | 309.439 | 2.234348 | 0.124648 | 17.92532 | 7.48E-72 | 1.67E-70 |
| IL21R | protein_coding | 320.5362 | 2.610485 | 0.145637 | 17.92463 | 7.57E-72 | 1.69E-70 |
| IYD | protein_coding | 471.9355 | -4.27096 | 0.238499 | -17.9076 | 1.03E-71 | 2.28E-70 |
| PPFIA4 | protein_coding | 1707.451 | 3.14256 | 0.175543 | 17.90192 | 1.14E-71 | 2.52E-70 |
| SPTB | protein_coding | 202.8948 | -2.66102 | 0.148701 | -17.8951 | 1.29E-71 | 2.84E-70 |
| HAPLN3 | protein_coding | 587.0598 | 2.400209 | 0.134246 | 17.87921 | 1.71E-71 | 3.77E-70 |
| PCDH9 | protein_coding | 320.0256 | -3.32373 | 0.186092 | -17.8606 | 2.39E-71 | 5.24E-70 |
| SNX20 | protein_coding | 570.5199 | 2.452808 | 0.137335 | 17.86 | 2.42E-71 | 5.29E-70 |
| CCL4 | protein_coding | 919.6252 | 2.652611 | 0.148526 | 17.85958 | 2.43E-71 | 5.32E-70 |
| PEG3 | protein_coding | 443.8724 | -2.40386 | 0.134665 | -17.8507 | 2.85E-71 | 6.23E-70 |
| CEP55 | protein_coding | 302.9903 | 2.616836 | 0.1466 | 17.8502 | 2.88E-71 | 6.28E-70 |
| HRH2 | protein_coding | 913.2892 | 3.437689 | 0.192676 | 17.8418 | 3.35E-71 | 7.27E-70 |
| CXCR3 | protein_coding | 330.4678 | 3.178324 | 0.178169 | 17.83881 | 3.53E-71 | 7.65E-70 |
| GNRH1 | protein_coding | 113.8946 | 2.788837 | 0.156393 | 17.83227 | 3.97E-71 | 8.57E-70 |
| HMOX1 | protein_coding | 16609.58 | 2.332541 | 0.130932 | 17.81486 | 5.42E-71 | 1.17E-69 |
| PGGHG | protein_coding | 6478.143 | 3.559476 | 0.199833 | 17.81227 | 5.68E-71 | 1.22E-69 |
| NDC80 | protein_coding | 247.6633 | 2.047583 | 0.115086 | 17.79175 | 8.19E-71 | 1.76E-69 |
| MMP25 | protein_coding | 151.0835 | 2.088199 | 0.117469 | 17.77658 | 1.07E-70 | 2.29E-69 |
| MAP6 | protein_coding | 358.2965 | -2.40835 | 0.135629 | -17.7569 | 1.52E-70 | 3.25E-69 |
| LGALS1 | protein_coding | 15411.7 | 2.165541 | 0.122034 | 17.74533 | 1.87E-70 | 3.98E-69 |
| MATK | protein_coding | 321.625 | 2.262465 | 0.127539 | 17.73941 | 2.08E-70 | 4.42E-69 |
| EBI3 | protein_coding | 335.3826 | 2.311908 | 0.130456 | 17.72174 | 2.85E-70 | 6.02E-69 |
| ADGRV1 | protein_coding | 273.5111 | -2.88243 | 0.16266 | -17.7205 | 2.91E-70 | 6.15E-69 |
| CXCR6 | protein_coding | 395.5107 | 2.678317 | 0.151297 | 17.7024 | 4.02E-70 | 8.46E-69 |
| NCF4 | protein_coding | 663.639 | 2.023773 | 0.114374 | 17.69439 | 4.63E-70 | 9.74E-69 |
| PLEKHN1 | protein_coding | 176.9004 | 2.478493 | 0.140083 | 17.69304 | 4.74E-70 | 9.96E-69 |
| LPAR5 | protein_coding | 423.1742 | 2.156512 | 0.122022 | 17.67308 | 6.76E-70 | 1.41E-68 |
| DTL | protein_coding | 350.8218 | 2.133059 | 0.12071 | 17.67099 | 7.02E-70 | 1.46E-68 |
| FCHO1 | protein_coding | 342.0855 | 2.641066 | 0.149555 | 17.6595 | 8.60E-70 | 1.78E-68 |
| ADAMDEC1 | protein_coding | 339.7428 | 4.289686 | 0.242913 | 17.65936 | 8.62E-70 | 1.78E-68 |
| ATG16L2 | protein_coding | 1520.864 | 2.307854 | 0.130728 | 17.65387 | 9.50E-70 | 1.96E-68 |
| ABCC3 | protein_coding | 10386.73 | 2.308899 | 0.130862 | 17.64374 | 1.14E-69 | 2.34E-68 |
| AC009779.3 | protein_coding | 209.1612 | 2.020979 | 0.114678 | 17.62302 | 1.64E-69 | 3.35E-68 |
| LZTS3 | protein_coding | 1381.415 | -2.30215 | 0.13065 | -17.6207 | 1.71E-69 | 3.48E-68 |
| DTX1 | protein_coding | 419.0301 | -2.45593 | 0.139381 | -17.6203 | 1.72E-69 | 3.50E-68 |
| CD6 | protein_coding | 621.5696 | 2.369993 | 0.134747 | 17.5885 | 3.02E-69 | 6.13E-68 |
| ANO4 | protein_coding | 502.8222 | 3.668773 | 0.208601 | 17.58753 | 3.07E-69 | 6.22E-68 |
| REEP6 | protein_coding | 325.3106 | -2.95686 | 0.168322 | -17.5667 | 4.43E-69 | 8.95E-68 |
| CD300C | protein_coding | 175.3955 | 2.290818 | 0.130468 | 17.55844 | 5.13E-69 | 1.03E-67 |
| HPCA | protein_coding | 90.16213 | 2.808273 | 0.160007 | 17.55099 | 5.85E-69 | 1.18E-67 |
| SIRPG | protein_coding | 253.558 | 3.380426 | 0.192876 | 17.52643 | 9.00E-69 | 1.81E-67 |
| GZMB | protein_coding | 340.2515 | 2.57343 | 0.14693 | 17.51471 | 1.11E-68 | 2.22E-67 |
| MAP4K1 | protein_coding | 569.7817 | 2.400307 | 0.137049 | 17.51418 | 1.12E-68 | 2.24E-67 |
| LOXL2 | protein_coding | 6963.108 | 2.689455 | 0.153607 | 17.50866 | 1.23E-68 | 2.46E-67 |
| SLC47A2 | protein_coding | 573.1953 | -3.92211 | 0.224383 | -17.4795 | 2.05E-68 | 4.08E-67 |
| PLCB2 | protein_coding | 1372.39 | 2.124312 | 0.121624 | 17.46621 | 2.59E-68 | 5.15E-67 |
| RGS5 | protein_coding | 125494.2 | 2.589284 | 0.148313 | 17.45823 | 2.98E-68 | 5.91E-67 |
| HAPLN1 | protein_coding | 1003.736 | 3.535967 | 0.202689 | 17.44528 | 3.74E-68 | 7.40E-67 |
| QRFPR | protein_coding | 745.5932 | 2.920567 | 0.16744 | 17.44244 | 3.93E-68 | 7.76E-67 |
| PARP15 | protein_coding | 173.7423 | 2.647303 | 0.151933 | 17.42418 | 5.41E-68 | 1.06E-66 |
| CD8B | protein_coding | 405.2165 | 3.407978 | 0.195675 | 17.41653 | 6.18E-68 | 1.21E-66 |
| SLAMF8 | protein_coding | 1130.645 | 2.712988 | 0.155819 | 17.41118 | 6.79E-68 | 1.33E-66 |
| SLC14A1 | protein_coding | 1083.791 | -3.01083 | 0.173018 | -17.4018 | 7.99E-68 | 1.57E-66 |
| SERPINA5 | protein_coding | 3133.441 | -4.86806 | 0.279852 | -17.3951 | 8.98E-68 | 1.75E-66 |
| TPX2 | protein_coding | 834.3267 | 2.513807 | 0.144691 | 17.3736 | 1.31E-67 | 2.54E-66 |
| NCF1 | protein_coding | 229.462 | 2.297765 | 0.13239 | 17.356 | 1.78E-67 | 3.44E-66 |
| CDH4 | protein_coding | 1616.717 | 4.071333 | 0.234804 | 17.33925 | 2.38E-67 | 4.58E-66 |
| GPC3 | protein_coding | 1435.73 | -3.69178 | 0.21298 | -17.3339 | 2.61E-67 | 5.01E-66 |
| NCAPG | protein_coding | 241.0718 | 2.377373 | 0.137198 | 17.32806 | 2.89E-67 | 5.54E-66 |
| LAG3 | protein_coding | 599.3004 | 3.572056 | 0.206247 | 17.31934 | 3.36E-67 | 6.44E-66 |
| OR51E1 | protein_coding | 470.5354 | 2.494946 | 0.144142 | 17.30894 | 4.03E-67 | 7.71E-66 |
| NOD2 | protein_coding | 273.7528 | 2.447662 | 0.14146 | 17.30283 | 4.48E-67 | 8.56E-66 |
| TNFSF13B | protein_coding | 922.9452 | 2.511299 | 0.145178 | 17.29811 | 4.86E-67 | 9.27E-66 |
| TEX11 | protein_coding | 335.7677 | 4.227784 | 0.244607 | 17.28399 | 6.21E-67 | 1.18E-65 |
| CACNA2D3 | protein_coding | 165.8128 | -2.09924 | 0.121578 | -17.2666 | 8.40E-67 | 1.59E-65 |
| SIGLEC1 | protein_coding | 1343.192 | 2.530947 | 0.146596 | 17.26475 | 8.67E-67 | 1.64E-65 |
| UBASH3A | protein_coding | 174.7968 | 2.786653 | 0.161471 | 17.25788 | 9.76E-67 | 1.84E-65 |
| RIMKLA | protein_coding | 1748.332 | 2.324658 | 0.134785 | 17.2471 | 1.18E-66 | 2.22E-65 |
| SPINK13 | protein_coding | 366.2616 | 4.644774 | 0.269424 | 17.23965 | 1.34E-66 | 2.51E-65 |
| FFAR4 | protein_coding | 99.16496 | 2.762676 | 0.160327 | 17.23147 | 1.54E-66 | 2.89E-65 |
| HPD | protein_coding | 2414.44 | -4.85705 | 0.281955 | -17.2263 | 1.69E-66 | 3.15E-65 |
| ST6GAL1 | protein_coding | 10307.82 | -2.18231 | 0.126707 | -17.2233 | 1.78E-66 | 3.31E-65 |
| SH2D2A | protein_coding | 345.6558 | 2.744456 | 0.159556 | 17.20061 | 2.63E-66 | 4.88E-65 |
| CD7 | protein_coding | 467.2347 | 2.757346 | 0.160326 | 17.19832 | 2.73E-66 | 5.07E-65 |
| CSTA | protein_coding | 209.9084 | 2.338903 | 0.136011 | 17.19639 | 2.83E-66 | 5.23E-65 |
| CD86 | protein_coding | 979.5037 | 2.13136 | 0.123949 | 17.19551 | 2.87E-66 | 5.31E-65 |
| FAM222A | protein_coding | 296.2623 | -2.73331 | 0.159073 | -17.1827 | 3.58E-66 | 6.60E-65 |
| ASPHD1 | protein_coding | 707.1591 | 2.666879 | 0.155262 | 17.17666 | 3.97E-66 | 7.31E-65 |
| CDON | protein_coding | 1632.384 | 2.222096 | 0.1294 | 17.17228 | 4.28E-66 | 7.85E-65 |
| RNF43 | protein_coding | 427.5819 | -2.56963 | 0.149902 | -17.1421 | 7.20E-66 | 1.31E-64 |
| HLA-DQB1 | protein_coding | 18470.73 | 2.297599 | 0.134101 | 17.13333 | 8.37E-66 | 1.52E-64 |
| ELOVL2 | protein_coding | 294.32 | 3.222985 | 0.188176 | 17.1275 | 9.25E-66 | 1.67E-64 |
| CHIT1 | protein_coding | 893.0524 | 5.071812 | 0.296165 | 17.12494 | 9.67E-66 | 1.75E-64 |
| CD27 | protein_coding | 540.4719 | 3.310157 | 0.193508 | 17.10606 | 1.34E-65 | 2.41E-64 |
| DEGS2 | protein_coding | 395.3556 | -2.97343 | 0.173855 | -17.1029 | 1.41E-65 | 2.54E-64 |
| SLFN12L | protein_coding | 129.9689 | 2.577656 | 0.150719 | 17.10245 | 1.42E-65 | 2.55E-64 |
| HOMER1 | protein_coding | 509.6925 | -2.20283 | 0.128905 | -17.0888 | 1.80E-65 | 3.21E-64 |
| CD3D | protein_coding | 590.5015 | 2.889088 | 0.169088 | 17.08631 | 1.88E-65 | 3.35E-64 |
| IQGAP3 | protein_coding | 520.9327 | 2.492018 | 0.145906 | 17.07965 | 2.10E-65 | 3.74E-64 |
| ANXA2R | protein_coding | 245.8835 | 2.040391 | 0.119478 | 17.0775 | 2.18E-65 | 3.87E-64 |
| PCSK6 | protein_coding | 5302.269 | 2.967805 | 0.173825 | 17.07353 | 2.34E-65 | 4.14E-64 |
| ADGRE1 | protein_coding | 223.0033 | 2.742649 | 0.160709 | 17.06591 | 2.66E-65 | 4.71E-64 |
| AOAH | protein_coding | 1223.089 | 2.161647 | 0.126931 | 17.03007 | 4.91E-65 | 8.60E-64 |
| TGFBI | protein_coding | 92268.61 | 4.135594 | 0.242929 | 17.02391 | 5.46E-65 | 9.52E-64 |
| CD96 | protein_coding | 667.5425 | 2.467049 | 0.145063 | 17.00674 | 7.32E-65 | 1.27E-63 |
| CYGB | protein_coding | 2550.042 | 2.030365 | 0.119414 | 17.00278 | 7.83E-65 | 1.36E-63 |
| GPRIN1 | protein_coding | 309.5936 | 2.18664 | 0.12876 | 16.98235 | 1.11E-64 | 1.92E-63 |
| ZNF469 | protein_coding | 311.2095 | 2.055446 | 0.121038 | 16.98176 | 1.12E-64 | 1.94E-63 |
| PLEK | protein_coding | 2089.601 | 2.244914 | 0.13224 | 16.97612 | 1.23E-64 | 2.13E-63 |
| FMO5 | protein_coding | 721.5349 | -2.4745 | 0.146231 | -16.9218 | 3.11E-64 | 5.31E-63 |
| UBD | protein_coding | 1457.14 | 3.170285 | 0.187423 | 16.91517 | 3.48E-64 | 5.92E-63 |
| SCG2 | protein_coding | 442.7055 | 3.920085 | 0.231813 | 16.91056 | 3.76E-64 | 6.39E-63 |
| ATP2B2 | protein_coding | 877.4237 | 3.402566 | 0.201644 | 16.87413 | 6.97E-64 | 1.18E-62 |
| SLC11A1 | protein_coding | 735.5174 | 2.39206 | 0.141806 | 16.86857 | 7.66E-64 | 1.30E-62 |
| MICALL2 | protein_coding | 1554.493 | 2.062591 | 0.122357 | 16.85709 | 9.31E-64 | 1.57E-62 |
| ASPM | protein_coding | 367.3659 | 2.626152 | 0.155803 | 16.85557 | 9.55E-64 | 1.61E-62 |
| DOCK2 | protein_coding | 2029.567 | 2.216261 | 0.131533 | 16.84952 | 1.06E-63 | 1.78E-62 |
| KCNE4 | protein_coding | 2058.58 | 2.048983 | 0.121733 | 16.83184 | 1.43E-63 | 2.38E-62 |
| CENPM | protein_coding | 172.4904 | 2.22272 | 0.13207 | 16.82986 | 1.47E-63 | 2.46E-62 |
| GTSE1 | protein_coding | 200.1876 | 2.346694 | 0.139448 | 16.8285 | 1.51E-63 | 2.51E-62 |
| SUSD4 | protein_coding | 524.4631 | -3.6674 | 0.217949 | -16.8269 | 1.55E-63 | 2.58E-62 |
| MARVELD2 | protein_coding | 954.7888 | -2.39374 | 0.14248 | -16.8006 | 2.42E-63 | 4.00E-62 |
| NT5DC3 | protein_coding | 2457.235 | 2.263261 | 0.134762 | 16.79455 | 2.68E-63 | 4.42E-62 |
| ZBP1 | protein_coding | 146.4483 | 2.867809 | 0.171155 | 16.75564 | 5.15E-63 | 8.40E-62 |
| BATF3 | protein_coding | 216.8241 | 2.133327 | 0.127548 | 16.72564 | 8.53E-63 | 1.38E-61 |
| ADA | protein_coding | 738.1137 | 2.242233 | 0.134229 | 16.70455 | 1.21E-62 | 1.97E-61 |
| COX4I2 | protein_coding | 715.6091 | 2.868204 | 0.171995 | 16.67606 | 1.96E-62 | 3.16E-61 |
| GZMK | protein_coding | 939.8879 | 3.334825 | 0.200057 | 16.66935 | 2.19E-62 | 3.53E-61 |
| SKA3 | protein_coding | 129.7824 | 2.087109 | 0.125223 | 16.66718 | 2.27E-62 | 3.66E-61 |
| GFI1 | protein_coding | 159.314 | 2.468812 | 0.148374 | 16.63915 | 3.63E-62 | 5.81E-61 |
| KIF4A | protein_coding | 293.8921 | 2.225932 | 0.133825 | 16.63319 | 4.01E-62 | 6.39E-61 |
| HSD3B7 | protein_coding | 4329.934 | 2.245095 | 0.135143 | 16.61279 | 5.63E-62 | 8.95E-61 |
| PDE1A | protein_coding | 2307.019 | -2.80327 | 0.16875 | -16.612 | 5.70E-62 | 9.05E-61 |
| HES4 | protein_coding | 540.8393 | 2.321447 | 0.139769 | 16.60921 | 5.98E-62 | 9.46E-61 |
| COL4A4 | protein_coding | 2042.834 | -2.17487 | 0.131016 | -16.6001 | 6.96E-62 | 1.10E-60 |
| DPEP1 | protein_coding | 2337.828 | -4.37976 | 0.264039 | -16.5875 | 8.58E-62 | 1.35E-60 |
| KIAA0895L | protein_coding | 1526.868 | 2.052917 | 0.123798 | 16.58274 | 9.29E-62 | 1.46E-60 |
| PTGDR | protein_coding | 92.09363 | 2.183277 | 0.131681 | 16.58006 | 9.71E-62 | 1.53E-60 |
| RTEL1-TNFRSF6B | protein_coding | 198.3641 | 2.462924 | 0.148673 | 16.56605 | 1.23E-61 | 1.92E-60 |
| PTTG1 | protein_coding | 411.1035 | 2.387956 | 0.144201 | 16.55994 | 1.36E-61 | 2.13E-60 |
| PLK1 | protein_coding | 315.6259 | 2.396478 | 0.144742 | 16.55695 | 1.43E-61 | 2.23E-60 |
| SLC26A4 | protein_coding | 233.1911 | -3.27269 | 0.19803 | -16.5263 | 2.37E-61 | 3.70E-60 |
| ADAMTS14 | protein_coding | 190.9108 | 3.40568 | 0.206256 | 16.51187 | 3.01E-61 | 4.70E-60 |
| CDH16 | protein_coding | 21406.09 | -2.36558 | 0.143296 | -16.5083 | 3.20E-61 | 4.97E-60 |
| TBX15 | protein_coding | 339.4553 | 3.023124 | 0.183396 | 16.48413 | 4.77E-61 | 7.39E-60 |
| CD3E | protein_coding | 1679.464 | 2.657462 | 0.161688 | 16.43574 | 1.06E-60 | 1.63E-59 |
| EIF4EBP1 | protein_coding | 2471.728 | 2.140797 | 0.130402 | 16.41693 | 1.45E-60 | 2.22E-59 |
| BUB1 | protein_coding | 340.813 | 2.409664 | 0.146812 | 16.41328 | 1.54E-60 | 2.35E-59 |
| SLAMF7 | protein_coding | 1178.398 | 2.816876 | 0.171707 | 16.4051 | 1.76E-60 | 2.69E-59 |
| DNAJB13 | protein_coding | 208.1705 | 3.709292 | 0.226299 | 16.39114 | 2.21E-60 | 3.38E-59 |
| CLDN10 | protein_coding | 2243.905 | -2.30311 | 0.140592 | -16.3815 | 2.59E-60 | 3.95E-59 |
| NUAK2 | protein_coding | 1870.203 | -2.11607 | 0.129225 | -16.375 | 2.88E-60 | 4.38E-59 |
| BIRC3 | protein_coding | 9949.352 | 2.290616 | 0.139991 | 16.36264 | 3.53E-60 | 5.36E-59 |
| S100A2 | protein_coding | 823.5878 | -3.45316 | 0.211051 | -16.3617 | 3.59E-60 | 5.43E-59 |
| B4GALNT1 | protein_coding | 878.1105 | 4.002803 | 0.244707 | 16.35753 | 3.84E-60 | 5.81E-59 |
| SPN | protein_coding | 1171.004 | 2.124449 | 0.129891 | 16.35566 | 3.96E-60 | 5.98E-59 |
| MELK | protein_coding | 215.0241 | 2.385406 | 0.145864 | 16.35367 | 4.10E-60 | 6.17E-59 |
| SPC24 | protein_coding | 220.6672 | 2.335888 | 0.142978 | 16.33743 | 5.35E-60 | 8.02E-59 |
| TIGIT | protein_coding | 384.3143 | 2.885555 | 0.17685 | 16.31643 | 7.54E-60 | 1.13E-58 |
| TESPA1 | protein_coding | 275.016 | 2.384037 | 0.146189 | 16.30793 | 8.67E-60 | 1.29E-58 |
| HLA-DQA1 | protein_coding | 15119.76 | 2.195329 | 0.134745 | 16.29246 | 1.12E-59 | 1.66E-58 |
| FOXM1 | protein_coding | 607.7758 | 2.440196 | 0.149784 | 16.29146 | 1.13E-59 | 1.68E-58 |
| CA8 | protein_coding | 346.6203 | -3.29439 | 0.20269 | -16.2534 | 2.11E-59 | 3.09E-58 |
| IKZF1 | protein_coding | 1428.413 | 2.049877 | 0.126248 | 16.23696 | 2.76E-59 | 4.04E-58 |
| LIMD2 | protein_coding | 1287.853 | 2.013382 | 0.124074 | 16.22727 | 3.24E-59 | 4.72E-58 |
| TICRR | protein_coding | 108.6813 | 2.288056 | 0.141091 | 16.2169 | 3.83E-59 | 5.57E-58 |
| SLAMF6 | protein_coding | 525.7128 | 2.5212 | 0.155644 | 16.19852 | 5.17E-59 | 7.49E-58 |
| GABRE | protein_coding | 1486.924 | 2.930429 | 0.180992 | 16.19092 | 5.84E-59 | 8.45E-58 |
| RAD54L | protein_coding | 159.3988 | 2.448399 | 0.151344 | 16.17775 | 7.24E-59 | 1.04E-57 |
| CDHR1 | protein_coding | 1574.039 | 4.205362 | 0.260687 | 16.13187 | 1.52E-58 | 2.18E-57 |
| RNF175 | protein_coding | 101.8924 | 2.397705 | 0.14872 | 16.1223 | 1.78E-58 | 2.54E-57 |
| TOP2A | protein_coding | 1301.122 | 2.322138 | 0.144115 | 16.11311 | 2.06E-58 | 2.94E-57 |
| TSPAN32 | protein_coding | 124.9121 | 2.01109 | 0.12484 | 16.10939 | 2.19E-58 | 3.12E-57 |
| PIEZO2 | protein_coding | 1763.93 | 2.127567 | 0.132103 | 16.10539 | 2.34E-58 | 3.32E-57 |
| LRRC25 | protein_coding | 1045.133 | 2.302097 | 0.142939 | 16.10544 | 2.34E-58 | 3.32E-57 |
| FPR3 | protein_coding | 2030.294 | 2.185562 | 0.135728 | 16.10248 | 2.45E-58 | 3.47E-57 |
| MMP9 | protein_coding | 1396.691 | 3.785474 | 0.235283 | 16.08901 | 3.05E-58 | 4.31E-57 |
| SPTBN2 | protein_coding | 2802.349 | -4.35616 | 0.270824 | -16.0849 | 3.26E-58 | 4.60E-57 |
| EME1 | protein_coding | 87.33459 | 2.097571 | 0.130455 | 16.07894 | 3.58E-58 | 5.05E-57 |
| SIRPB1 | protein_coding | 356.3885 | 2.67503 | 0.166515 | 16.06484 | 4.50E-58 | 6.31E-57 |
| FAM193B | protein_coding | 2619.105 | 2.106301 | 0.131241 | 16.04912 | 5.80E-58 | 8.13E-57 |
| E2F8 | protein_coding | 114.5761 | 2.640453 | 0.1646 | 16.04166 | 6.54E-58 | 9.16E-57 |
| MEFV | protein_coding | 114.3463 | 2.2486 | 0.140447 | 16.01033 | 1.08E-57 | 1.51E-56 |
| CDC6 | protein_coding | 292.2654 | 2.000374 | 0.12515 | 15.98376 | 1.66E-57 | 2.30E-56 |
| AC040162.1 | protein_coding | 154.6022 | 2.099891 | 0.131427 | 15.97763 | 1.83E-57 | 2.53E-56 |
| DACH1 | protein_coding | 603.7025 | -2.37988 | 0.149057 | -15.9663 | 2.19E-57 | 3.03E-56 |
| SLC6A1 | protein_coding | 464.7133 | 2.284319 | 0.14313 | 15.95976 | 2.44E-57 | 3.36E-56 |
| PDCD1 | protein_coding | 289.7317 | 3.327655 | 0.208932 | 15.92699 | 4.12E-57 | 5.63E-56 |
| BIRC5 | protein_coding | 446.7764 | 2.494343 | 0.156684 | 15.91958 | 4.63E-57 | 6.32E-56 |
| MMP11 | protein_coding | 1029.062 | 2.041848 | 0.128307 | 15.91379 | 5.08E-57 | 6.93E-56 |
| TMEM233 | protein_coding | 429.139 | 2.073518 | 0.130409 | 15.90018 | 6.32E-57 | 8.59E-56 |
| RAC2 | protein_coding | 2337.437 | 2.092413 | 0.131815 | 15.87384 | 9.62E-57 | 1.30E-55 |
| PYCARD | protein_coding | 915.5559 | 2.083678 | 0.131342 | 15.86454 | 1.12E-56 | 1.50E-55 |
| HLA-DQB2 | protein_coding | 1817.891 | 2.812423 | 0.177326 | 15.86014 | 1.20E-56 | 1.61E-55 |
| SLC29A4 | protein_coding | 2622.354 | 2.19968 | 0.138695 | 15.85988 | 1.20E-56 | 1.61E-55 |
| FAM151A | protein_coding | 1274.436 | -3.96209 | 0.249908 | -15.8542 | 1.31E-56 | 1.76E-55 |
| SIT1 | protein_coding | 244.9003 | 2.703174 | 0.170523 | 15.85225 | 1.36E-56 | 1.82E-55 |
| IKZF3 | protein_coding | 1015.853 | 2.536822 | 0.160032 | 15.85198 | 1.36E-56 | 1.82E-55 |
| CD3G | protein_coding | 420.1243 | 2.541308 | 0.160322 | 15.85132 | 1.38E-56 | 1.84E-55 |
| SH2D5 | protein_coding | 125.9493 | 2.477826 | 0.156437 | 15.83915 | 1.67E-56 | 2.23E-55 |
| ALDOC | protein_coding | 5083.689 | 2.294323 | 0.144944 | 15.829 | 1.96E-56 | 2.61E-55 |
| PRR7 | protein_coding | 169.0912 | 2.395565 | 0.151366 | 15.82635 | 2.05E-56 | 2.72E-55 |
| SLC52A3 | protein_coding | 365.5247 | -2.95128 | 0.186787 | -15.8002 | 3.10E-56 | 4.10E-55 |
| GATA3 | protein_coding | 1063.087 | -3.10275 | 0.197093 | -15.7426 | 7.72E-56 | 1.01E-54 |
| CKAP2L | protein_coding | 168.9897 | 2.249469 | 0.143104 | 15.71911 | 1.12E-55 | 1.46E-54 |
| RIN1 | protein_coding | 565.4298 | 2.05955 | 0.131037 | 15.71735 | 1.15E-55 | 1.50E-54 |
| BCL2A1 | protein_coding | 251.737 | 2.579398 | 0.164244 | 15.70465 | 1.41E-55 | 1.83E-54 |
| UBE2C | protein_coding | 418.958 | 2.964088 | 0.188883 | 15.69271 | 1.70E-55 | 2.20E-54 |
| MAPK8IP3 | protein_coding | 3871.585 | 2.090112 | 0.133244 | 15.6863 | 1.88E-55 | 2.43E-54 |
| HOXB6 | protein_coding | 819.8499 | -2.09595 | 0.133889 | -15.6543 | 3.10E-55 | 4.01E-54 |
| KCNJ12 | protein_coding | 189.2414 | -2.32305 | 0.148733 | -15.6189 | 5.41E-55 | 6.95E-54 |
| PIMREG | protein_coding | 117.3783 | 2.317874 | 0.148534 | 15.60504 | 6.73E-55 | 8.60E-54 |
| RHOH | protein_coding | 379.2977 | 2.204685 | 0.141457 | 15.58557 | 9.12E-55 | 1.16E-53 |
| PTPN22 | protein_coding | 320.2542 | 2.032737 | 0.130502 | 15.57632 | 1.05E-54 | 1.33E-53 |
| CD52 | protein_coding | 1331.93 | 2.11711 | 0.135973 | 15.5701 | 1.16E-54 | 1.47E-53 |
| GZMM | protein_coding | 130.0781 | 2.094718 | 0.134552 | 15.56813 | 1.20E-54 | 1.51E-53 |
| TENM1 | protein_coding | 1353.632 | 2.426783 | 0.155906 | 15.5657 | 1.24E-54 | 1.57E-53 |
| AIM2 | protein_coding | 135.6451 | 3.046827 | 0.195922 | 15.55123 | 1.56E-54 | 1.96E-53 |
| SEZ6L2 | protein_coding | 4518.913 | 2.294738 | 0.147866 | 15.51902 | 2.58E-54 | 3.22E-53 |
| SIGLEC7 | protein_coding | 185.4188 | 2.119178 | 0.136737 | 15.4982 | 3.57E-54 | 4.43E-53 |
| NMB | protein_coding | 1453.716 | 2.587507 | 0.166957 | 15.49806 | 3.58E-54 | 4.43E-53 |
| BMP6 | protein_coding | 736.3333 | -2.115 | 0.136507 | -15.4937 | 3.83E-54 | 4.72E-53 |
| AQP3 | protein_coding | 9552.658 | -2.19788 | 0.14188 | -15.4912 | 3.98E-54 | 4.91E-53 |
| NPIPB5 | protein_coding | 269.4184 | 2.958833 | 0.191093 | 15.48371 | 4.47E-54 | 5.50E-53 |
| MAP3K7CL | protein_coding | 1814.592 | 2.121535 | 0.137275 | 15.45458 | 7.03E-54 | 8.60E-53 |
| ESRRG | protein_coding | 1427.724 | -3.08811 | 0.200071 | -15.4351 | 9.51E-54 | 1.16E-52 |
| GGACT | protein_coding | 705.6065 | -2.38929 | 0.154948 | -15.4199 | 1.20E-53 | 1.46E-52 |
| ADAMTS10 | protein_coding | 983.8089 | 2.169821 | 0.140797 | 15.41095 | 1.38E-53 | 1.68E-52 |
| ADAMTSL4 | protein_coding | 1106.518 | 2.106297 | 0.136708 | 15.40731 | 1.46E-53 | 1.77E-52 |
| MT1G | protein_coding | 3976.985 | -4.43781 | 0.288076 | -15.405 | 1.51E-53 | 1.84E-52 |
| MMP16 | protein_coding | 328.719 | 2.717428 | 0.176817 | 15.36862 | 2.66E-53 | 3.21E-52 |
| PLD4 | protein_coding | 672.5942 | 2.259544 | 0.14705 | 15.36586 | 2.77E-53 | 3.34E-52 |
| P4HA3 | protein_coding | 455.4524 | 2.858741 | 0.186636 | 15.31721 | 5.87E-53 | 7.03E-52 |
| COL5A2 | protein_coding | 6369.363 | 2.068386 | 0.135039 | 15.31697 | 5.89E-53 | 7.05E-52 |
| PRRT2 | protein_coding | 216.7835 | 2.427821 | 0.158967 | 15.27253 | 1.17E-52 | 1.38E-51 |
| EIF4A1 | protein_coding | 521.3001 | 2.11615 | 0.138765 | 15.24988 | 1.65E-52 | 1.95E-51 |
| SSC4D | protein_coding | 155.2364 | -2.23491 | 0.146687 | -15.2358 | 2.05E-52 | 2.40E-51 |
| STMN3 | protein_coding | 4173.609 | 2.160924 | 0.141954 | 15.22271 | 2.50E-52 | 2.92E-51 |
| FBP1 | protein_coding | 3709.726 | -2.34675 | 0.154355 | -15.2036 | 3.35E-52 | 3.89E-51 |
| SLC17A4 | protein_coding | 2849.431 | 3.579224 | 0.235543 | 15.19564 | 3.78E-52 | 4.38E-51 |
| DEPDC1 | protein_coding | 151.6679 | 2.389047 | 0.15727 | 15.19073 | 4.07E-52 | 4.71E-51 |
| RASD1 | protein_coding | 3375.078 | -2.52757 | 0.166389 | -15.1907 | 4.07E-52 | 4.71E-51 |
| CTHRC1 | protein_coding | 1582.362 | 3.163736 | 0.208452 | 15.17731 | 5.00E-52 | 5.77E-51 |
| MSR1 | protein_coding | 3407.818 | 2.049468 | 0.135239 | 15.15438 | 7.09E-52 | 8.15E-51 |
| DCXR | protein_coding | 2487.225 | -2.062 | 0.136452 | -15.1115 | 1.36E-51 | 1.55E-50 |
| SIX1 | protein_coding | 129.4062 | 2.426361 | 0.160715 | 15.09727 | 1.69E-51 | 1.92E-50 |
| UBAP1L | protein_coding | 311.9207 | 2.137065 | 0.141574 | 15.09506 | 1.75E-51 | 1.98E-50 |
| CCDC160 | protein_coding | 145.1961 | -2.39606 | 0.158846 | -15.0841 | 2.06E-51 | 2.33E-50 |
| KIF20A | protein_coding | 413.1077 | 2.369375 | 0.157107 | 15.08128 | 2.15E-51 | 2.44E-50 |
| SH2D1A | protein_coding | 273.0298 | 2.469937 | 0.163887 | 15.07094 | 2.52E-51 | 2.85E-50 |
| CARMIL2 | protein_coding | 206.84 | 2.518881 | 0.167487 | 15.03929 | 4.06E-51 | 4.56E-50 |
| AHSA2 | protein_coding | 2572.402 | 2.088223 | 0.138931 | 15.03069 | 4.62E-51 | 5.17E-50 |
| CXCL13 | protein_coding | 375.8491 | 4.213309 | 0.280645 | 15.01296 | 6.04E-51 | 6.74E-50 |
| AC023509.3 | protein_coding | 104.8536 | 2.320983 | 0.154721 | 15.00107 | 7.22E-51 | 8.05E-50 |
| PADI2 | protein_coding | 1366.366 | -2.47064 | 0.164942 | -14.9789 | 1.01E-50 | 1.12E-49 |
| CD180 | protein_coding | 423.6802 | 2.115446 | 0.141343 | 14.96675 | 1.21E-50 | 1.34E-49 |
| ATG9B | protein_coding | 288.6714 | 2.947968 | 0.196984 | 14.96555 | 1.23E-50 | 1.36E-49 |
| ADSSL1 | protein_coding | 4226.348 | 2.464785 | 0.164697 | 14.96554 | 1.23E-50 | 1.36E-49 |
| GALNT3 | protein_coding | 602.9321 | -2.58181 | 0.172684 | -14.9511 | 1.53E-50 | 1.69E-49 |
| CGN | protein_coding | 1481.387 | -2.44525 | 0.16367 | -14.9401 | 1.81E-50 | 1.98E-49 |
| MPPED2 | protein_coding | 423.4156 | -2.55506 | 0.171308 | -14.915 | 2.63E-50 | 2.88E-49 |
| PAQR6 | protein_coding | 187.0893 | 2.611719 | 0.175124 | 14.91352 | 2.69E-50 | 2.94E-49 |
| NLGN1 | protein_coding | 1901.009 | 2.006909 | 0.134814 | 14.88648 | 4.03E-50 | 4.39E-49 |
| NFKBID | protein_coding | 461.4915 | 2.181034 | 0.146577 | 14.87983 | 4.46E-50 | 4.84E-49 |
| IL4I1 | protein_coding | 920.6829 | 2.128455 | 0.143087 | 14.8752 | 4.78E-50 | 5.18E-49 |
| RAP1GAP | protein_coding | 4820.202 | -2.57866 | 0.173355 | -14.8751 | 4.79E-50 | 5.19E-49 |
| AGER | protein_coding | 349.1342 | 2.093405 | 0.140758 | 14.87234 | 4.98E-50 | 5.40E-49 |
| ACOT11 | protein_coding | 795.7402 | -2.02548 | 0.136356 | -14.8544 | 6.52E-50 | 7.03E-49 |
| POLQ | protein_coding | 104.457 | 2.136868 | 0.143866 | 14.85322 | 6.63E-50 | 7.14E-49 |
| SLC13A3 | protein_coding | 4220.128 | -4.6788 | 0.315063 | -14.8504 | 6.92E-50 | 7.45E-49 |
| TMEM72 | protein_coding | 2608.024 | -3.02579 | 0.203993 | -14.8328 | 8.99E-50 | 9.65E-49 |
| IFI30 | protein_coding | 127.0726 | 2.328326 | 0.157063 | 14.82414 | 1.02E-49 | 1.09E-48 |
| C16orf74 | protein_coding | 498.7078 | 3.094269 | 0.208756 | 14.82241 | 1.05E-49 | 1.12E-48 |
| SLC9A3 | protein_coding | 1835.266 | -3.50932 | 0.236809 | -14.8192 | 1.10E-49 | 1.17E-48 |
| CD5 | protein_coding | 463.4349 | 2.112077 | 0.142589 | 14.81235 | 1.22E-49 | 1.30E-48 |
| MKI67 | protein_coding | 1261.682 | 2.219709 | 0.14991 | 14.80697 | 1.32E-49 | 1.40E-48 |
| ALDOB | protein_coding | 38628.28 | -4.71867 | 0.319652 | -14.7619 | 2.58E-49 | 2.72E-48 |
| KIAA0319 | protein_coding | 137.2467 | 2.824803 | 0.191392 | 14.75928 | 2.68E-49 | 2.82E-48 |
| PLEKHG4 | protein_coding | 944.4441 | 2.122815 | 0.143974 | 14.74445 | 3.34E-49 | 3.51E-48 |
| NGF | protein_coding | 423.155 | 2.137586 | 0.14498 | 14.74396 | 3.36E-49 | 3.54E-48 |
| GPM6B | protein_coding | 385.2735 | -2.16154 | 0.14668 | -14.7364 | 3.76E-49 | 3.95E-48 |
| CGNL1 | protein_coding | 8389.979 | -2.14476 | 0.14574 | -14.7163 | 5.06E-49 | 5.30E-48 |
| PIPOX | protein_coding | 1584.139 | -2.97009 | 0.201869 | -14.7129 | 5.33E-49 | 5.57E-48 |
| HHLA2 | protein_coding | 3716.549 | 3.126004 | 0.212601 | 14.70362 | 6.11E-49 | 6.37E-48 |
| NR3C2 | protein_coding | 1418.268 | -2.06282 | 0.140479 | -14.6842 | 8.14E-49 | 8.44E-48 |
| PLAU | protein_coding | 3900.114 | -2.15082 | 0.146523 | -14.6791 | 8.78E-49 | 9.08E-48 |
| ARHGAP33 | protein_coding | 649.6054 | 2.111247 | 0.143896 | 14.67205 | 9.74E-49 | 1.01E-47 |
| COL4A5 | protein_coding | 930.7618 | -2.3265 | 0.158669 | -14.6626 | 1.12E-48 | 1.15E-47 |
| E2F7 | protein_coding | 110.146 | 2.430565 | 0.165952 | 14.64616 | 1.43E-48 | 1.46E-47 |
| LYZ | protein_coding | 10485.99 | 2.442 | 0.166803 | 14.64002 | 1.56E-48 | 1.60E-47 |
| PBK | protein_coding | 151.1916 | 2.108921 | 0.144295 | 14.61535 | 2.24E-48 | 2.29E-47 |
| DLK2 | protein_coding | 120.1681 | 2.349 | 0.160786 | 14.60947 | 2.44E-48 | 2.49E-47 |
| LY6G5B | protein_coding | 146.7097 | 2.353359 | 0.161121 | 14.60612 | 2.57E-48 | 2.62E-47 |
| SLC2A3 | protein_coding | 7493.649 | 2.029679 | 0.139079 | 14.59368 | 3.08E-48 | 3.13E-47 |
| CRYGS | protein_coding | 147.7447 | 2.104024 | 0.144523 | 14.55842 | 5.16E-48 | 5.21E-47 |
| RAB33A | protein_coding | 105.1914 | 2.158199 | 0.148644 | 14.51922 | 9.15E-48 | 9.14E-47 |
| ANLN | protein_coding | 670.7846 | 2.313332 | 0.159414 | 14.51147 | 1.02E-47 | 1.02E-46 |
| SKA1 | protein_coding | 90.92095 | 2.142435 | 0.147718 | 14.50352 | 1.15E-47 | 1.14E-46 |
| ITK | protein_coding | 510.5272 | 2.081184 | 0.143606 | 14.49227 | 1.36E-47 | 1.34E-46 |
| CACNA2D4 | protein_coding | 408.3937 | 2.08755 | 0.144061 | 14.49072 | 1.39E-47 | 1.37E-46 |
| TEN1-CDK3 | protein_coding | 91.37336 | 2.015929 | 0.139365 | 14.46506 | 2.01E-47 | 1.98E-46 |
| ARL4D | protein_coding | 793.465 | -2.68721 | 0.185805 | -14.4625 | 2.09E-47 | 2.05E-46 |
| LINC01125 | protein_coding | 156.4022 | 2.035257 | 0.140791 | 14.45586 | 2.30E-47 | 2.26E-46 |
| HSPB7 | protein_coding | 681.9767 | -3.28849 | 0.227922 | -14.4281 | 3.44E-47 | 3.35E-46 |
| SLC17A2 | protein_coding | 242.5634 | 3.293416 | 0.228508 | 14.41272 | 4.30E-47 | 4.17E-46 |
| PERM1 | protein_coding | 412.4014 | 3.104532 | 0.215479 | 14.40758 | 4.64E-47 | 4.48E-46 |
| MAL2 | protein_coding | 4643.526 | -2.75667 | 0.191413 | -14.4017 | 5.05E-47 | 4.87E-46 |
| ACAN | protein_coding | 2531.8 | 2.609501 | 0.181257 | 14.39666 | 5.43E-47 | 5.23E-46 |
| APOB | protein_coding | 1515.995 | 4.180992 | 0.290889 | 14.37317 | 7.63E-47 | 7.31E-46 |
| CCL20 | protein_coding | 1295.317 | 3.649717 | 0.254301 | 14.35197 | 1.04E-46 | 9.91E-46 |
| ZNF831 | protein_coding | 170.1549 | 2.219152 | 0.154739 | 14.34128 | 1.21E-46 | 1.15E-45 |
| C8orf4 | protein_coding | 4058.552 | -2.17012 | 0.151363 | -14.3372 | 1.28E-46 | 1.22E-45 |
| HOXB9 | protein_coding | 747.8612 | -3.29928 | 0.230378 | -14.3212 | 1.61E-46 | 1.53E-45 |
| HCST | protein_coding | 335.2275 | 2.031925 | 0.141887 | 14.32077 | 1.62E-46 | 1.54E-45 |
| MSH5 | protein_coding | 243.0211 | 2.171268 | 0.151663 | 14.31638 | 1.73E-46 | 1.64E-45 |
| DHRS9 | protein_coding | 108.5841 | 2.406142 | 0.168103 | 14.31351 | 1.80E-46 | 1.70E-45 |
| TIMP1 | protein_coding | 25177.34 | 2.085279 | 0.145712 | 14.31098 | 1.87E-46 | 1.77E-45 |
| ADAM8 | protein_coding | 657.6123 | 2.145663 | 0.150835 | 14.22522 | 6.39E-46 | 5.95E-45 |
| NPIPB3 | protein_coding | 111.6042 | 2.16242 | 0.15207 | 14.21993 | 6.89E-46 | 6.41E-45 |
| RRM2 | protein_coding | 769.0478 | 2.172822 | 0.152868 | 14.2137 | 7.53E-46 | 6.97E-45 |
| CHL1 | protein_coding | 906.0201 | -3.34106 | 0.235267 | -14.2012 | 9.01E-46 | 8.32E-45 |
| FAM46B | protein_coding | 221.0695 | -2.56033 | 0.180303 | -14.2002 | 9.14E-46 | 8.43E-45 |
| FCRL3 | protein_coding | 181.4757 | 2.799999 | 0.197411 | 14.18361 | 1.16E-45 | 1.07E-44 |
| KCNMA1 | protein_coding | 3464.071 | 2.267112 | 0.160158 | 14.15544 | 1.73E-45 | 1.58E-44 |
| GAPT | protein_coding | 349.1536 | 2.068291 | 0.146228 | 14.14427 | 2.03E-45 | 1.85E-44 |
| LY9 | protein_coding | 182.3936 | 2.096844 | 0.148915 | 14.08082 | 4.98E-45 | 4.50E-44 |
| RPS6KA6 | protein_coding | 565.3946 | -3.01341 | 0.214999 | -14.0159 | 1.25E-44 | 1.11E-43 |
| SCNN1D | protein_coding | 235.1858 | 2.399875 | 0.171425 | 13.99959 | 1.57E-44 | 1.40E-43 |
| MALL | protein_coding | 262.277 | 2.060559 | 0.147504 | 13.96951 | 2.39E-44 | 2.12E-43 |
| AP1M2 | protein_coding | 1323.198 | -2.4607 | 0.176308 | -13.9568 | 2.86E-44 | 2.52E-43 |
| CLDN11 | protein_coding | 217.8754 | -2.47709 | 0.177525 | -13.9535 | 3.00E-44 | 2.64E-43 |
| CD36 | protein_coding | 8584.411 | 2.172497 | 0.155709 | 13.95231 | 3.05E-44 | 2.68E-43 |
| NPNT | protein_coding | 4420.788 | -2.05198 | 0.147098 | -13.9497 | 3.16E-44 | 2.77E-43 |
| OTOGL | protein_coding | 183.8632 | -2.51587 | 0.180583 | -13.932 | 4.05E-44 | 3.54E-43 |
| COL4A3 | protein_coding | 1674.736 | -2.03444 | 0.146046 | -13.9301 | 4.16E-44 | 3.64E-43 |
| ALOX5 | protein_coding | 2486.372 | 2.088241 | 0.150113 | 13.91116 | 5.42E-44 | 4.72E-43 |
| C1QL1 | protein_coding | 1930.669 | 3.71318 | 0.267098 | 13.90195 | 6.16E-44 | 5.36E-43 |
| GOLGA8A | protein_coding | 1307.815 | 2.825054 | 0.203318 | 13.89478 | 6.81E-44 | 5.91E-43 |
| SLITRK5 | protein_coding | 399.4508 | 3.413659 | 0.24594 | 13.88007 | 8.37E-44 | 7.23E-43 |
| VWCE | protein_coding | 841.863 | 2.782009 | 0.20088 | 13.84913 | 1.29E-43 | 1.11E-42 |
| TACSTD2 | protein_coding | 4673.409 | -3.51614 | 0.254083 | -13.8386 | 1.49E-43 | 1.28E-42 |
| COL1A1 | protein_coding | 60218.38 | 2.817631 | 0.204402 | 13.78473 | 3.15E-43 | 2.67E-42 |
| SYT7 | protein_coding | 475.0205 | -3.36291 | 0.244361 | -13.7621 | 4.31E-43 | 3.65E-42 |
| GATM | protein_coding | 27006.89 | -2.20152 | 0.160159 | -13.7459 | 5.39E-43 | 4.54E-42 |
| BICDL1 | protein_coding | 3188.061 | -2.01643 | 0.146768 | -13.7389 | 5.93E-43 | 4.99E-42 |
| TPRG1 | protein_coding | 152.3568 | 2.21731 | 0.161426 | 13.73573 | 6.20E-43 | 5.21E-42 |
| FREM1 | protein_coding | 617.9417 | -3.35959 | 0.244712 | -13.7288 | 6.83E-43 | 5.72E-42 |
| TUBB2B | protein_coding | 229.5121 | -2.59872 | 0.189783 | -13.6931 | 1.12E-42 | 9.30E-42 |
| GMPR | protein_coding | 731.5064 | -2.29711 | 0.167842 | -13.6861 | 1.23E-42 | 1.02E-41 |
| ADAMTS16 | protein_coding | 297.305 | -2.67354 | 0.19537 | -13.6845 | 1.26E-42 | 1.05E-41 |
| TRAT1 | protein_coding | 146.9154 | 2.339917 | 0.171215 | 13.66657 | 1.61E-42 | 1.33E-41 |
| ILDR1 | protein_coding | 625.1539 | -2.02356 | 0.148183 | -13.6558 | 1.86E-42 | 1.54E-41 |
| C7 | protein_coding | 12548.83 | -3.34297 | 0.244861 | -13.6526 | 1.95E-42 | 1.61E-41 |
| TLR7 | protein_coding | 758.2664 | 2.060774 | 0.150983 | 13.64905 | 2.05E-42 | 1.69E-41 |
| F2RL3 | protein_coding | 1770.707 | 2.346158 | 0.172092 | 13.63315 | 2.54E-42 | 2.09E-41 |
| DCLK1 | protein_coding | 1683.871 | 2.145214 | 0.157434 | 13.62614 | 2.80E-42 | 2.29E-41 |
| CYS1 | protein_coding | 6941.063 | -2.09695 | 0.154005 | -13.6161 | 3.21E-42 | 2.62E-41 |
| AQP9 | protein_coding | 765.4757 | 3.396747 | 0.249965 | 13.58888 | 4.66E-42 | 3.78E-41 |
| CCL4L2 | protein_coding | 447.1463 | 2.564567 | 0.188953 | 13.57253 | 5.83E-42 | 4.72E-41 |
| AGMAT | protein_coding | 2993.681 | -2.46501 | 0.181778 | -13.5606 | 6.86E-42 | 5.55E-41 |
| BTNL9 | protein_coding | 3359.824 | 2.160201 | 0.159328 | 13.55823 | 7.08E-42 | 5.73E-41 |
| CD200R1 | protein_coding | 221.0224 | 2.213554 | 0.164368 | 13.46705 | 2.44E-41 | 1.95E-40 |
| MAN1C1 | protein_coding | 1296.65 | -2.20443 | 0.164076 | -13.4354 | 3.75E-41 | 2.97E-40 |
| CNGA1 | protein_coding | 176.1328 | -2.50607 | 0.186859 | -13.4115 | 5.18E-41 | 4.08E-40 |
| NEK2 | protein_coding | 139.8159 | 2.068735 | 0.154343 | 13.4035 | 5.77E-41 | 4.54E-40 |
| FBLN7 | protein_coding | 522.2489 | 2.191608 | 0.163873 | 13.37378 | 8.61E-41 | 6.72E-40 |
| CDH23 | protein_coding | 526.1227 | 2.349791 | 0.175985 | 13.35221 | 1.15E-40 | 8.95E-40 |
| CLSTN2 | protein_coding | 969.9669 | -2.72055 | 0.203833 | -13.347 | 1.23E-40 | 9.59E-40 |
| GLDC | protein_coding | 1375.121 | -2.35537 | 0.177303 | -13.2845 | 2.85E-40 | 2.19E-39 |
| TLR8 | protein_coding | 396.2101 | 2.101219 | 0.158561 | 13.25181 | 4.40E-40 | 3.37E-39 |
| OR51E2 | protein_coding | 182.9502 | 2.205196 | 0.166696 | 13.22881 | 5.98E-40 | 4.55E-39 |
| DNASE1L3 | protein_coding | 605.533 | -2.17205 | 0.164269 | -13.2226 | 6.50E-40 | 4.93E-39 |
| PLEKHB1 | protein_coding | 569.1993 | -2.3194 | 0.175722 | -13.1992 | 8.86E-40 | 6.69E-39 |
| MLIP | protein_coding | 120.1342 | 2.126435 | 0.161244 | 13.18765 | 1.03E-39 | 7.78E-39 |
| HIST1H3H | protein_coding | 178.4397 | 2.573825 | 0.195525 | 13.16367 | 1.42E-39 | 1.06E-38 |
| ABCA12 | protein_coding | 487.5567 | 2.456473 | 0.186997 | 13.13643 | 2.04E-39 | 1.52E-38 |
| THEMIS | protein_coding | 250.6952 | 2.173725 | 0.165612 | 13.12544 | 2.35E-39 | 1.75E-38 |
| EXOC3L4 | protein_coding | 737.8573 | 2.053506 | 0.156522 | 13.11957 | 2.54E-39 | 1.89E-38 |
| COL9A2 | protein_coding | 514.9372 | -2.19013 | 0.167234 | -13.0962 | 3.46E-39 | 2.56E-38 |
| CLEC5A | protein_coding | 299.1737 | 2.024987 | 0.155087 | 13.05712 | 5.79E-39 | 4.27E-38 |
| C19orf33 | protein_coding | 1705.925 | 2.556828 | 0.19584 | 13.05571 | 5.90E-39 | 4.34E-38 |
| CARD11 | protein_coding | 1012.489 | 2.11934 | 0.162897 | 13.01029 | 1.07E-38 | 7.81E-38 |
| C3orf36 | protein_coding | 161.4395 | 2.099244 | 0.161411 | 13.00558 | 1.14E-38 | 8.29E-38 |
| SCART1 | protein_coding | 140.6582 | 2.221546 | 0.1711 | 12.98387 | 1.51E-38 | 1.09E-37 |
| ADD2 | protein_coding | 230.3104 | 2.703113 | 0.208435 | 12.96862 | 1.84E-38 | 1.33E-37 |
| CDCA7 | protein_coding | 220.0792 | 2.013865 | 0.155373 | 12.9615 | 2.02E-38 | 1.46E-37 |
| PRTG | protein_coding | 166.7449 | -2.05159 | 0.158423 | -12.9501 | 2.35E-38 | 1.69E-37 |
| GPAT3 | protein_coding | 1855.464 | -2.49393 | 0.192767 | -12.9375 | 2.76E-38 | 1.98E-37 |
| DAO | protein_coding | 651.5949 | -3.23247 | 0.249885 | -12.9358 | 2.83E-38 | 2.03E-37 |
| ITGB6 | protein_coding | 2211.721 | -2.59095 | 0.200358 | -12.9316 | 2.99E-38 | 2.14E-37 |
| ADH6 | protein_coding | 686.5196 | -2.62212 | 0.202874 | -12.9248 | 3.26E-38 | 2.33E-37 |
| ASPDH | protein_coding | 325.7257 | -2.73684 | 0.212379 | -12.8866 | 5.36E-38 | 3.80E-37 |
| COL21A1 | protein_coding | 743.9829 | 2.201585 | 0.171609 | 12.82911 | 1.13E-37 | 7.91E-37 |
| FAM153A | protein_coding | 263.1633 | 3.236236 | 0.252291 | 12.82742 | 1.15E-37 | 8.08E-37 |
| PABPC1L | protein_coding | 1129.875 | 2.226192 | 0.173575 | 12.82552 | 1.18E-37 | 8.28E-37 |
| HSD11B2 | protein_coding | 7996.275 | -2.80918 | 0.219194 | -12.8159 | 1.34E-37 | 9.34E-37 |
| CARD14 | protein_coding | 187.8233 | 2.648592 | 0.206713 | 12.8129 | 1.39E-37 | 9.71E-37 |
| MAL | protein_coding | 5804.382 | -3.30225 | 0.258258 | -12.7866 | 1.95E-37 | 1.36E-36 |
| TREX2 | protein_coding | 186.5749 | 2.659889 | 0.208061 | 12.7842 | 2.01E-37 | 1.40E-36 |
| TMEM30B | protein_coding | 1030.041 | -2.92779 | 0.229362 | -12.7649 | 2.57E-37 | 1.78E-36 |
| LRAT | protein_coding | 261.0649 | 2.261056 | 0.177374 | 12.7474 | 3.22E-37 | 2.22E-36 |
| SMIM5 | protein_coding | 511.9631 | -3.16425 | 0.24966 | -12.6742 | 8.21E-37 | 5.59E-36 |
| CACNA1F | protein_coding | 109.3872 | 2.279127 | 0.180108 | 12.6542 | 1.06E-36 | 7.21E-36 |
| ALDH4A1 | protein_coding | 5149.348 | -2.06118 | 0.16373 | -12.5889 | 2.43E-36 | 1.63E-35 |
| HLA-DOB | protein_coding | 283.1736 | 2.173688 | 0.173067 | 12.55984 | 3.51E-36 | 2.34E-35 |
| LDHD | protein_coding | 1199.159 | -2.27153 | 0.1812 | -12.536 | 4.74E-36 | 3.14E-35 |
| ABCB4 | protein_coding | 333.0788 | 2.078229 | 0.166077 | 12.51368 | 6.28E-36 | 4.15E-35 |
| PRND | protein_coding | 345.439 | 3.290549 | 0.263747 | 12.47616 | 1.01E-35 | 6.60E-35 |
| MAMDC2 | protein_coding | 202.4412 | -2.02097 | 0.162761 | -12.4167 | 2.12E-35 | 1.37E-34 |
| PILRB | protein_coding | 533.5096 | 2.425948 | 0.196033 | 12.37523 | 3.56E-35 | 2.29E-34 |
| GAS1 | protein_coding | 352.7368 | -2.09248 | 0.169275 | -12.3615 | 4.22E-35 | 2.70E-34 |
| OVOL1 | protein_coding | 234.2904 | -2.34363 | 0.189819 | -12.3466 | 5.08E-35 | 3.24E-34 |
| KDF1 | protein_coding | 298.9187 | -2.04054 | 0.165727 | -12.3127 | 7.74E-35 | 4.90E-34 |
| B4GALNT2 | protein_coding | 583.5855 | -3.60512 | 0.293319 | -12.2908 | 1.01E-34 | 6.38E-34 |
| TJP3 | protein_coding | 290.3892 | -2.77928 | 0.226185 | -12.2877 | 1.05E-34 | 6.63E-34 |
| CRB2 | protein_coding | 312.4541 | -2.91006 | 0.237183 | -12.2693 | 1.32E-34 | 8.29E-34 |
| PROM2 | protein_coding | 2007.908 | -3.5258 | 0.287911 | -12.2461 | 1.76E-34 | 1.10E-33 |
| SUCNR1 | protein_coding | 1366.669 | -2.15715 | 0.176518 | -12.2205 | 2.41E-34 | 1.49E-33 |
| HOXB8 | protein_coding | 633.6969 | -2.24486 | 0.183712 | -12.2194 | 2.45E-34 | 1.51E-33 |
| GRIA4 | protein_coding | 914.121 | 3.646263 | 0.299985 | 12.15482 | 5.41E-34 | 3.30E-33 |
| OCLN | protein_coding | 643.6063 | -2.09757 | 0.172676 | -12.1475 | 5.92E-34 | 3.60E-33 |
| ACSL6 | protein_coding | 216.6229 | -2.16217 | 0.17811 | -12.1395 | 6.52E-34 | 3.96E-33 |
| SNAP25 | protein_coding | 461.4181 | 2.003543 | 0.165127 | 12.13333 | 7.03E-34 | 4.27E-33 |
| LAX1 | protein_coding | 154.0468 | 2.021325 | 0.167197 | 12.08952 | 1.20E-33 | 7.21E-33 |
| ANK2 | protein_coding | 5941.966 | -2.15014 | 0.178267 | -12.0614 | 1.69E-33 | 1.01E-32 |
| CORO2B | protein_coding | 414.0253 | -2.01647 | 0.167239 | -12.0574 | 1.77E-33 | 1.06E-32 |
| GPR35 | protein_coding | 445.2443 | 2.434363 | 0.202194 | 12.03973 | 2.20E-33 | 1.30E-32 |
| FBXO2 | protein_coding | 475.2095 | -2.48732 | 0.20821 | -11.9462 | 6.80E-33 | 3.95E-32 |
| LAD1 | protein_coding | 1224.998 | -2.67258 | 0.224542 | -11.9024 | 1.15E-32 | 6.63E-32 |
| CYP3A5 | protein_coding | 1917.946 | 2.080033 | 0.174772 | 11.9014 | 1.16E-32 | 6.71E-32 |
| MGARP | protein_coding | 427.8882 | 2.988765 | 0.251138 | 11.90087 | 1.17E-32 | 6.75E-32 |
| NAP1L2 | protein_coding | 353.269 | -2.2585 | 0.189866 | -11.8953 | 1.25E-32 | 7.21E-32 |
| WISP2 | protein_coding | 371.5521 | 3.363305 | 0.28393 | 11.84554 | 2.27E-32 | 1.30E-31 |
| DERL3 | protein_coding | 613.7996 | 2.184661 | 0.1851 | 11.80263 | 3.78E-32 | 2.15E-31 |
| BDNF | protein_coding | 250.1746 | 2.120475 | 0.179785 | 11.79451 | 4.17E-32 | 2.36E-31 |
| PHYHD1 | protein_coding | 706.6472 | -2.24019 | 0.190202 | -11.778 | 5.07E-32 | 2.86E-31 |
| IL2RA | protein_coding | 270.3046 | 2.222798 | 0.189937 | 11.70282 | 1.23E-31 | 6.85E-31 |
| APOL1 | protein_coding | 20516.52 | 2.068902 | 0.177051 | 11.68533 | 1.51E-31 | 8.40E-31 |
| G6PC | protein_coding | 1229.762 | -3.51359 | 0.300871 | -11.6781 | 1.65E-31 | 9.13E-31 |
| CHST13 | protein_coding | 614.043 | 2.011467 | 0.172395 | 11.66778 | 1.86E-31 | 1.03E-30 |
| ARC | protein_coding | 226.1013 | -2.09256 | 0.179507 | -11.6573 | 2.11E-31 | 1.16E-30 |
| MRGPRF | protein_coding | 285.129 | -2.19569 | 0.188677 | -11.6373 | 2.66E-31 | 1.46E-30 |
| MT1F | protein_coding | 1764.291 | -2.1489 | 0.185466 | -11.5865 | 4.82E-31 | 2.62E-30 |
| ANK1 | protein_coding | 645.1907 | 2.196879 | 0.189901 | 11.56854 | 5.95E-31 | 3.22E-30 |
| YJEFN3 | protein_coding | 185.1736 | 2.099016 | 0.18282 | 11.4813 | 1.64E-30 | 8.72E-30 |
| TMEM92 | protein_coding | 481.9682 | 2.795993 | 0.244486 | 11.43619 | 2.76E-30 | 1.45E-29 |
| SCNN1A | protein_coding | 5613.262 | -2.92899 | 0.256925 | -11.4002 | 4.17E-30 | 2.19E-29 |
| PRSS35 | protein_coding | 197.6895 | -2.1636 | 0.189903 | -11.3932 | 4.52E-30 | 2.37E-29 |
| MT3 | protein_coding | 913.4995 | 3.353728 | 0.294579 | 11.38482 | 4.98E-30 | 2.60E-29 |
| COL5A1 | protein_coding | 7318.554 | 2.046762 | 0.18039 | 11.34633 | 7.73E-30 | 4.03E-29 |
| C1QL4 | protein_coding | 443.9021 | 3.056599 | 0.26958 | 11.33839 | 8.47E-30 | 4.40E-29 |
| ANGPTL1 | protein_coding | 395.9623 | -2.56258 | 0.227281 | -11.275 | 1.74E-29 | 8.96E-29 |
| PRIMA1 | protein_coding | 2210.26 | 3.072936 | 0.273141 | 11.25036 | 2.31E-29 | 1.18E-28 |
| HLA-DQA2 | protein_coding | 3169.694 | 2.468213 | 0.220384 | 11.19962 | 4.09E-29 | 2.06E-28 |
| C2 | protein_coding | 2729.936 | 2.192062 | 0.196577 | 11.15119 | 7.07E-29 | 3.54E-28 |
| SIM1 | protein_coding | 1192.493 | -3.12567 | 0.28283 | -11.0514 | 2.16E-28 | 1.06E-27 |
| S100A14 | protein_coding | 313.5626 | -2.06007 | 0.186726 | -11.0326 | 2.66E-28 | 1.30E-27 |
| PCK1 | protein_coding | 15495.86 | -2.84807 | 0.258803 | -11.0048 | 3.62E-28 | 1.76E-27 |
| TFCP2L1 | protein_coding | 4486.064 | -3.25555 | 0.29619 | -10.9914 | 4.20E-28 | 2.03E-27 |
| PLXNA4 | protein_coding | 271.7666 | -2.41597 | 0.220222 | -10.9706 | 5.29E-28 | 2.55E-27 |
| C1orf116 | protein_coding | 496.0034 | -2.9034 | 0.264831 | -10.9632 | 5.74E-28 | 2.77E-27 |
| CADM3 | protein_coding | 963.898 | 3.007834 | 0.274783 | 10.94622 | 6.93E-28 | 3.33E-27 |
| DNASE1 | protein_coding | 1745.171 | -2.15815 | 0.197263 | -10.9405 | 7.38E-28 | 3.54E-27 |
| NTRK2 | protein_coding | 2980.114 | -2.07426 | 0.190157 | -10.9081 | 1.05E-27 | 5.03E-27 |
| SERPINE1 | protein_coding | 21482.91 | 2.265959 | 0.208185 | 10.88437 | 1.37E-27 | 6.49E-27 |
| KCNK3 | protein_coding | 5024.938 | 2.171047 | 0.199657 | 10.87387 | 1.54E-27 | 7.27E-27 |
| GRM8 | protein_coding | 496.3873 | 2.022388 | 0.186232 | 10.85951 | 1.80E-27 | 8.48E-27 |
| MOXD1 | protein_coding | 1282.437 | -2.26918 | 0.209471 | -10.8329 | 2.40E-27 | 1.13E-26 |
| CREB3L3 | protein_coding | 1192.84 | 2.698689 | 0.250277 | 10.78282 | 4.15E-27 | 1.93E-26 |
| L1CAM | protein_coding | 1997.798 | -3.19335 | 0.297244 | -10.7432 | 6.38E-27 | 2.95E-26 |
| B4GALNT3 | protein_coding | 602.9123 | -2.23307 | 0.208124 | -10.7295 | 7.40E-27 | 3.41E-26 |
| CHI3L2 | protein_coding | 380.4459 | 2.310384 | 0.216455 | 10.67374 | 1.35E-26 | 6.16E-26 |
| ADCY2 | protein_coding | 313.943 | 2.567076 | 0.24114 | 10.64557 | 1.83E-26 | 8.29E-26 |
| LGALS4 | protein_coding | 2275.225 | 3.540579 | 0.332796 | 10.63887 | 1.96E-26 | 8.88E-26 |
| PSAT1 | protein_coding | 1698.007 | -2.48634 | 0.234417 | -10.6065 | 2.78E-26 | 1.25E-25 |
| C21orf62 | protein_coding | 415.7575 | -2.25042 | 0.213643 | -10.5335 | 6.05E-26 | 2.68E-25 |
| ADGRG2 | protein_coding | 455.4984 | 2.446142 | 0.232316 | 10.52937 | 6.33E-26 | 2.80E-25 |
| CR1 | protein_coding | 258.808 | -2.01223 | 0.191106 | -10.5294 | 6.32E-26 | 2.80E-25 |
| SLC16A7 | protein_coding | 2514.94 | -2.15707 | 0.205618 | -10.4907 | 9.53E-26 | 4.18E-25 |
| FRZB | protein_coding | 7698.288 | 2.041298 | 0.194698 | 10.4844 | 1.02E-25 | 4.45E-25 |
| BSPRY | protein_coding | 526.3385 | -2.08172 | 0.199502 | -10.4346 | 1.72E-25 | 7.48E-25 |
| LRRC19 | protein_coding | 1280.708 | -2.01947 | 0.193541 | -10.4344 | 1.73E-25 | 7.50E-25 |
| PLXNB3 | protein_coding | 188.664 | 2.037604 | 0.195367 | 10.42963 | 1.82E-25 | 7.86E-25 |
| DCN | protein_coding | 7714.844 | -2.52804 | 0.243174 | -10.396 | 2.58E-25 | 1.11E-24 |
| TREM1 | protein_coding | 232.7204 | 2.130092 | 0.205547 | 10.36305 | 3.65E-25 | 1.56E-24 |
| HAO2 | protein_coding | 2095.858 | -2.48724 | 0.240628 | -10.3364 | 4.82E-25 | 2.05E-24 |
| CREG2 | protein_coding | 179.8106 | 2.007935 | 0.19496 | 10.29921 | 7.10E-25 | 3.00E-24 |
| SLC22A7 | protein_coding | 538.8492 | -3.13199 | 0.304308 | -10.2922 | 7.64E-25 | 3.22E-24 |
| TMC4 | protein_coding | 1569.158 | -2.08427 | 0.202512 | -10.2921 | 7.65E-25 | 3.22E-24 |
| HAVCR1 | protein_coding | 2939.516 | 2.125651 | 0.206775 | 10.28002 | 8.67E-25 | 3.64E-24 |
| PDGFRA | protein_coding | 1235.854 | -2.35822 | 0.229736 | -10.2649 | 1.01E-24 | 4.24E-24 |
| PLCXD3 | protein_coding | 410.4838 | -2.09631 | 0.204245 | -10.2637 | 1.03E-24 | 4.29E-24 |
| TREH | protein_coding | 269.2649 | -2.32779 | 0.227256 | -10.243 | 1.27E-24 | 5.29E-24 |
| C2orf40 | protein_coding | 331.9644 | -2.05461 | 0.201696 | -10.1867 | 2.27E-24 | 9.36E-24 |
| MELTF | protein_coding | 945.8226 | -2.47401 | 0.243316 | -10.1679 | 2.76E-24 | 1.13E-23 |
| MARCO | protein_coding | 292.6303 | 2.394727 | 0.236044 | 10.14526 | 3.48E-24 | 1.42E-23 |
| SORCS3 | protein_coding | 993.7854 | 3.189428 | 0.314855 | 10.12984 | 4.07E-24 | 1.65E-23 |
| LRRN2 | protein_coding | 628.5627 | -2.31939 | 0.230331 | -10.0698 | 7.51E-24 | 3.01E-23 |
| PRODH2 | protein_coding | 1827.878 | -2.26206 | 0.224709 | -10.0666 | 7.76E-24 | 3.11E-23 |
| SOSTDC1 | protein_coding | 539.9227 | -2.91228 | 0.289835 | -10.0481 | 9.37E-24 | 3.75E-23 |
| CBLN4 | protein_coding | 261.6254 | 2.071379 | 0.207865 | 9.965017 | 2.17E-23 | 8.55E-23 |
| PPP1R14D | protein_coding | 389.7085 | 2.675587 | 0.269427 | 9.930648 | 3.06E-23 | 1.20E-22 |
| ADTRP | protein_coding | 290.5124 | -2.00236 | 0.201828 | -9.92112 | 3.37E-23 | 1.32E-22 |
| SPINK5 | protein_coding | 218.6275 | 2.127689 | 0.214475 | 9.920444 | 3.39E-23 | 1.33E-22 |
| ADH1B | protein_coding | 2031.699 | -2.5921 | 0.263345 | -9.84298 | 7.35E-23 | 2.83E-22 |
| PTGER3 | protein_coding | 6673.343 | -2.09829 | 0.213918 | -9.80883 | 1.03E-22 | 3.95E-22 |
| MIOX | protein_coding | 6837.408 | -2.31621 | 0.237021 | -9.77219 | 1.48E-22 | 5.65E-22 |
| SCIN | protein_coding | 5108.054 | -2.08167 | 0.213672 | -9.74234 | 1.99E-22 | 7.55E-22 |
| MAP7D2 | protein_coding | 2074.549 | 2.39492 | 0.247767 | 9.666001 | 4.20E-22 | 1.57E-21 |
| ANXA3 | protein_coding | 845.4022 | -2.01058 | 0.208383 | -9.64849 | 4.99E-22 | 1.86E-21 |
| ATP6V1C2 | protein_coding | 454.4819 | -2.18111 | 0.228024 | -9.56526 | 1.12E-21 | 4.13E-21 |
| PRAME | protein_coding | 820.836 | 2.808163 | 0.293726 | 9.56048 | 1.17E-21 | 4.31E-21 |
| CLEC18A | protein_coding | 753.1516 | 2.062067 | 0.216761 | 9.513079 | 1.85E-21 | 6.73E-21 |
| MOGAT3 | protein_coding | 236.3286 | 2.259196 | 0.23811 | 9.488035 | 2.35E-21 | 8.52E-21 |
| PAH | protein_coding | 3915.783 | -2.95152 | 0.32162 | -9.17704 | 4.43E-20 | 1.53E-19 |
| DPT | protein_coding | 323.2252 | -2.15901 | 0.236296 | -9.13689 | 6.43E-20 | 2.20E-19 |
| CDH3 | protein_coding | 470.8631 | -2.27023 | 0.248871 | -9.12212 | 7.37E-20 | 2.51E-19 |
| TOX3 | protein_coding | 588.3599 | -2.23845 | 0.248272 | -9.01611 | 1.95E-19 | 6.52E-19 |
| FGA | protein_coding | 1997.696 | 3.325796 | 0.370236 | 8.982895 | 2.64E-19 | 8.77E-19 |
| SLC22A6 | protein_coding | 3233.488 | -2.90147 | 0.324574 | -8.93932 | 3.92E-19 | 1.29E-18 |
| SHISA3 | protein_coding | 1029.622 | -2.14353 | 0.240253 | -8.92197 | 4.58E-19 | 1.51E-18 |
| HMGCS2 | protein_coding | 4320.672 | -2.62819 | 0.294822 | -8.9145 | 4.90E-19 | 1.61E-18 |
| WT1 | protein_coding | 388.4019 | -2.48938 | 0.279386 | -8.9102 | 5.09E-19 | 1.68E-18 |
| VAT1L | protein_coding | 435.0035 | -2.39094 | 0.273371 | -8.74614 | 2.21E-18 | 7.06E-18 |
| IL1RL1 | protein_coding | 942.4098 | -2.083 | 0.243228 | -8.56398 | 1.09E-17 | 3.37E-17 |
| TFAP2A | protein_coding | 427.3506 | -2.08246 | 0.243911 | -8.5378 | 1.37E-17 | 4.21E-17 |
| MMP1 | protein_coding | 425.6776 | 2.110831 | 0.251626 | 8.388777 | 4.91E-17 | 1.47E-16 |
| ATRNL1 | protein_coding | 268.8792 | -2.0258 | 0.242529 | -8.35282 | 6.67E-17 | 1.99E-16 |
| GREM1 | protein_coding | 472.3398 | -2.41367 | 0.290671 | -8.30379 | 1.01E-16 | 2.98E-16 |
| SLC5A1 | protein_coding | 3072.442 | 2.116833 | 0.256587 | 8.249967 | 1.58E-16 | 4.63E-16 |
| PPP1R1A | protein_coding | 3004.987 | -2.63673 | 0.321465 | -8.20222 | 2.36E-16 | 6.84E-16 |
| REG1A | protein_coding | 3151.166 | 2.651607 | 0.323867 | 8.187342 | 2.67E-16 | 7.72E-16 |
| PKP1 | protein_coding | 569.1111 | 2.108123 | 0.264423 | 7.972553 | 1.55E-15 | 4.33E-15 |
| ANO5 | protein_coding | 381.1576 | -2.08013 | 0.261619 | -7.95098 | 1.85E-15 | 5.13E-15 |
| TMEM174 | protein_coding | 1409.843 | -2.25833 | 0.284709 | -7.93207 | 2.16E-15 | 5.96E-15 |
| IGLL5 | protein_coding | 1569.706 | 2.212853 | 0.279222 | 7.925075 | 2.28E-15 | 6.30E-15 |
| FGB | protein_coding | 7970.672 | 3.07275 | 0.401505 | 7.653072 | 1.96E-14 | 5.20E-14 |
| CYP4F3 | protein_coding | 660.952 | -2.14964 | 0.282329 | -7.61397 | 2.66E-14 | 7.01E-14 |
| PRR15L | protein_coding | 618.1925 | -2.11236 | 0.278597 | -7.58214 | 3.40E-14 | 8.92E-14 |
| ATP6V1B1 | protein_coding | 2204.651 | -2.70853 | 0.358407 | -7.55714 | 4.12E-14 | 1.08E-13 |
| NDNF | protein_coding | 1403.556 | -2.44349 | 0.338588 | -7.21669 | 5.33E-13 | 1.32E-12 |
| ALPI | protein_coding | 628.2234 | 2.455111 | 0.342716 | 7.163683 | 7.85E-13 | 1.93E-12 |
| SLC22A12 | protein_coding | 4310.653 | -2.03292 | 0.298687 | -6.80621 | 1.00E-11 | 2.31E-11 |
| SLC6A19 | protein_coding | 4478.364 | -2.20231 | 0.333963 | -6.59446 | 4.27E-11 | 9.47E-11 |
