# Supplementary material for: Correlation between Genes of the ceRNA Network and Tumor-Infiltrating Immune Cells and Their Biomarker Screening in Kidney Renal Clear Cell Carcinoma
Source: J Oncol. 2022 Aug 29;2022:4084461. doi: 10.1155/2022/4084461 (PMC9444395; doi:10.1155/2022/4084461)
Supplement: Supplementary Materials — Differentially expressed lncRNA, miRNAs, and mRNAs were put in Supplementary Material 1. Gene ID of lncRNAs, miRNAs and mRNAs in the ceRNA network were displayed in Supplementary Material 2. For a comprehensive digital IHC image analysis with Qupath, please refer to the protocol in Supplementary Material 3. [file 4084461.f1.zip › Supplementary Material 2.docx]

Table: LncRNAs, miRNAs and mRNAs in the ceRNA network.

| RNA type | Gene ID |
| --- | --- |
| LncRNA | AL365277.1, SNHG12, AC105020.1, DUXAP8, HCG27, AC019080.1,  LINC00887, EGFR-AS1, SLC25A5-AS1, TTC21B-AS1, LINC01886, OSTM1-AS1, AC078883.1, LINC00475, MIAT, SLC16A1-AS1, LINC02048, LINC01428, LINC01159, ALA590666.2, SERPINB9P1, LINC02015, DARS-AS1, AL096799.1, AL137186.2, LINC00342, AC011899.2, LINC00472, AL590094.1, LINC01426, MIR155HG, LINC00894, LINC00707, LINC00893, LINC00861, AC105202.1, PCED1B-AS1, FAM13A-AS1, LUCAT1, SEMA6A-AS1, PVT1, AP002884.3, LINC02381, ZFPM2-AS1, LINC02384, LINC01094, AC090197.1, LINC01152, LINC00944, AC156455.1, HIF1A-AS2, AL135999.1, AC103740.1, AC107021.2, LINC02188, LINC01355, AL031714.1 |
| miRNA | miR-122-5p, hsa-miR-129-5p, hsa-miR-141-3p, hsa-miR-155-5p, hsa-miR-200c-3p, hsa-miR-21-5p, hsa-miR-210-3p |
| mRNA | E2F2, ANLN, RUNX3, VIM, FOXC1, PRDM1, CDON, CLEC2D, ST6GAL1, PTHLH, SCD, COL1A1, VEGFA, TGFBI, SIX1, SEMA6D, CEP55, PCSK6, EFNA3, CCNA2, PLK2, NR3C2, MAMDC2, E2F7, PCLAF, DDIT4, NETO2, ADGRG2, SOX11, ZNF395, KLHL14, OCLN, L1CAM, MXD3 |
