# Supplementary material for: Correlation between Genes of the ceRNA Network and Tumor-Infiltrating Immune Cells and Their Biomarker Screening in Kidney Renal Clear Cell Carcinoma
Source: J Oncol. 2022 Aug 29;2022:4084461. doi: 10.1155/2022/4084461 (PMC9444395; doi:10.1155/2022/4084461)

Protocol for digital IHC analysis with Qupath(open source software for Quantitative Pathology, version 0.2.0). As an example, we here illustrate the process of CD4 expression analysis.

Step1: Estimating stain vectors. Set image type to “H-DAB” when opening the microarray. Then, draw a region that include tissue and background to estimate the vectors.


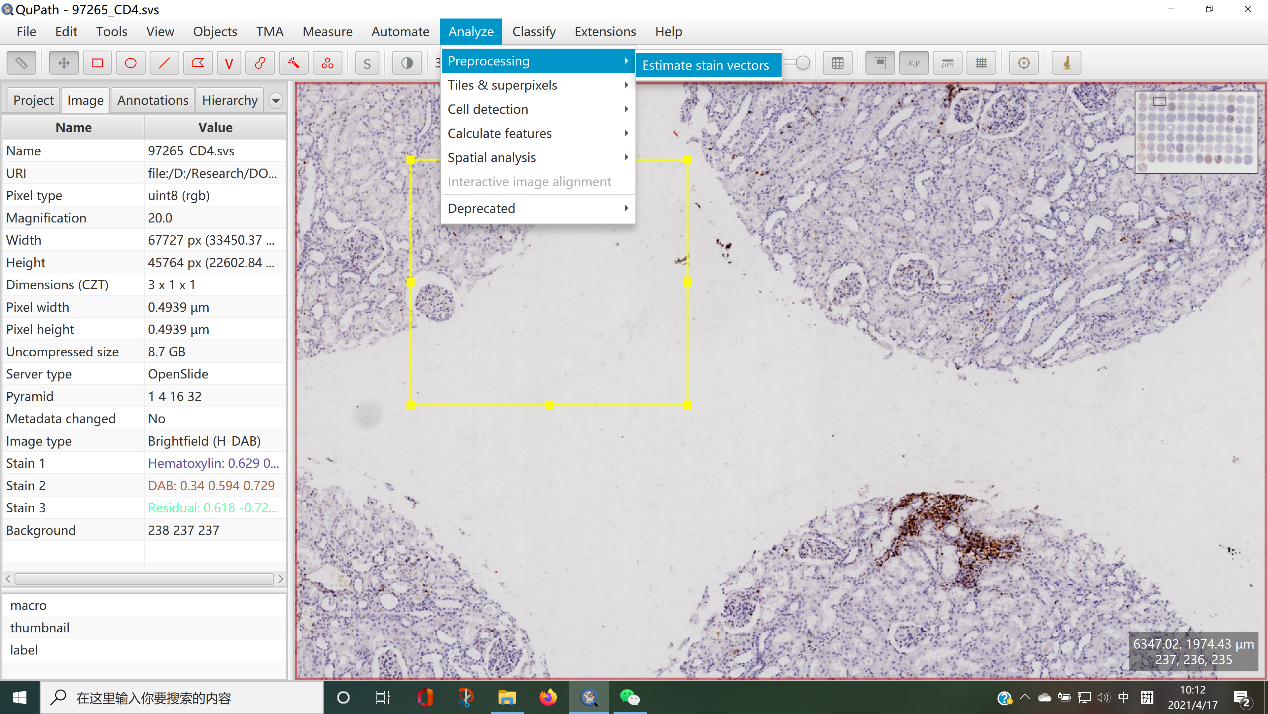


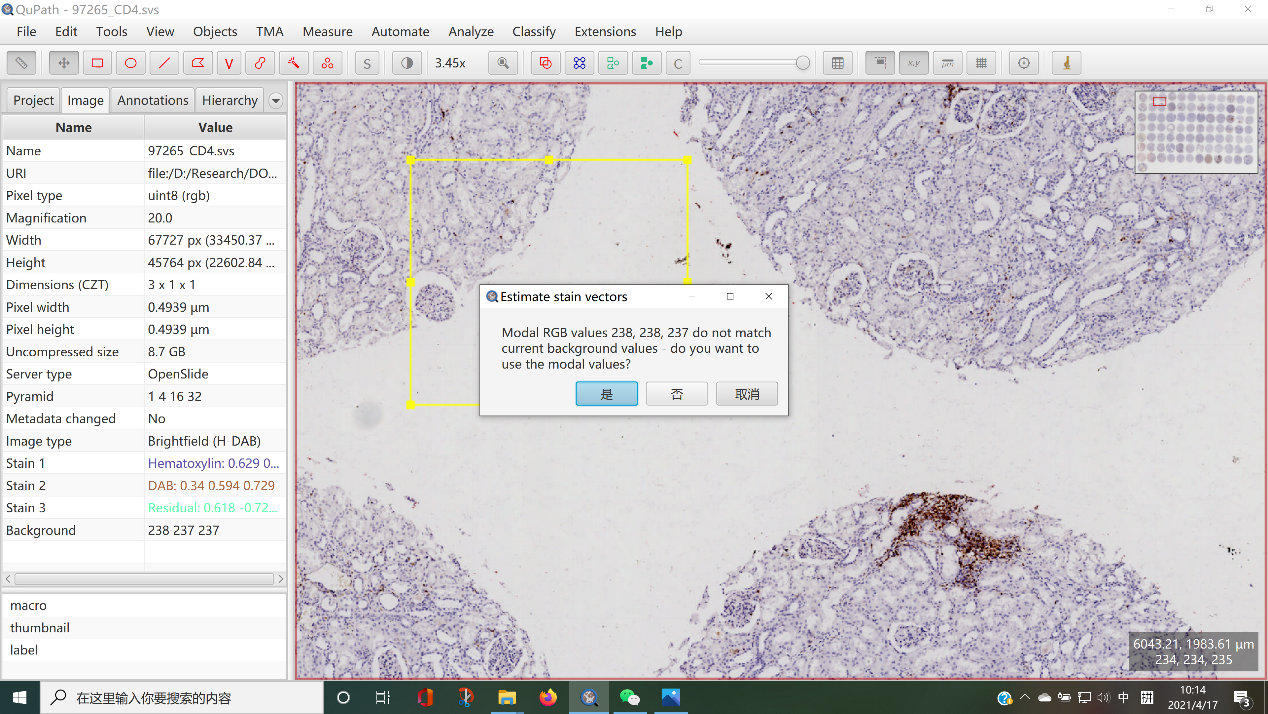


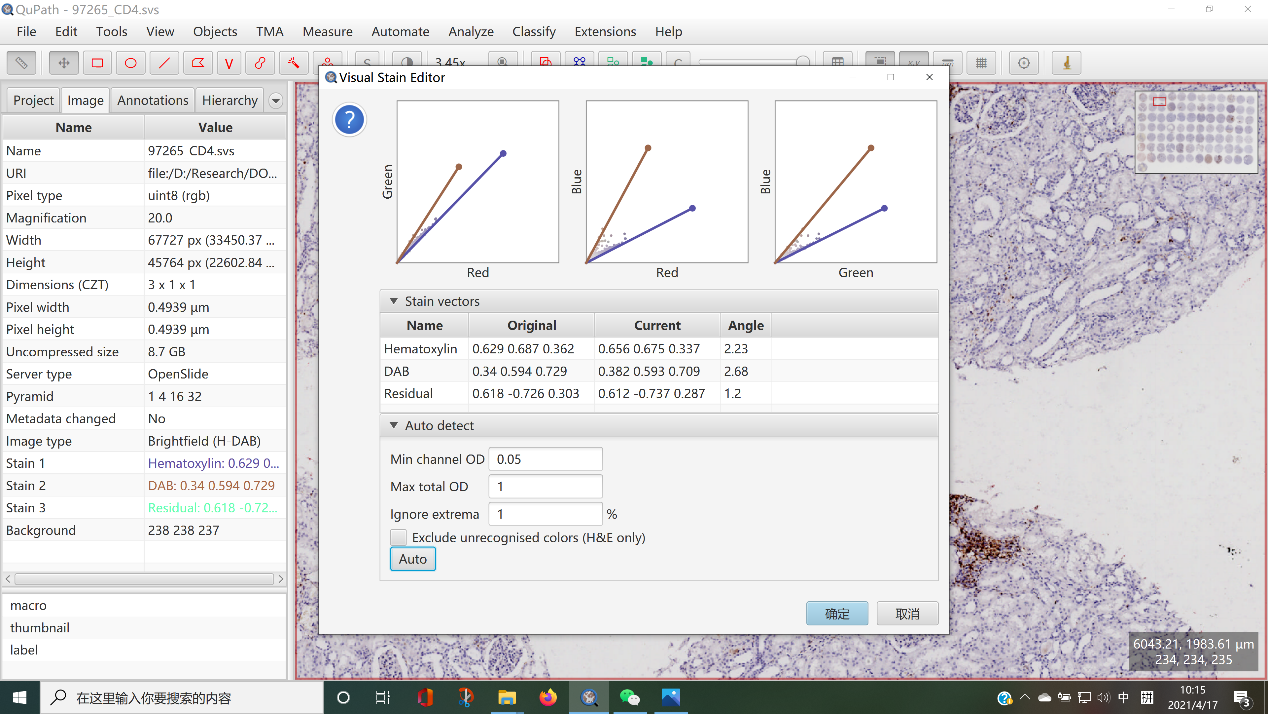


Step2: Annotating each core of microarray. In this step each core was assigned with a name,

peritumoral region of tumoral core and nonspecific staining was excluded.


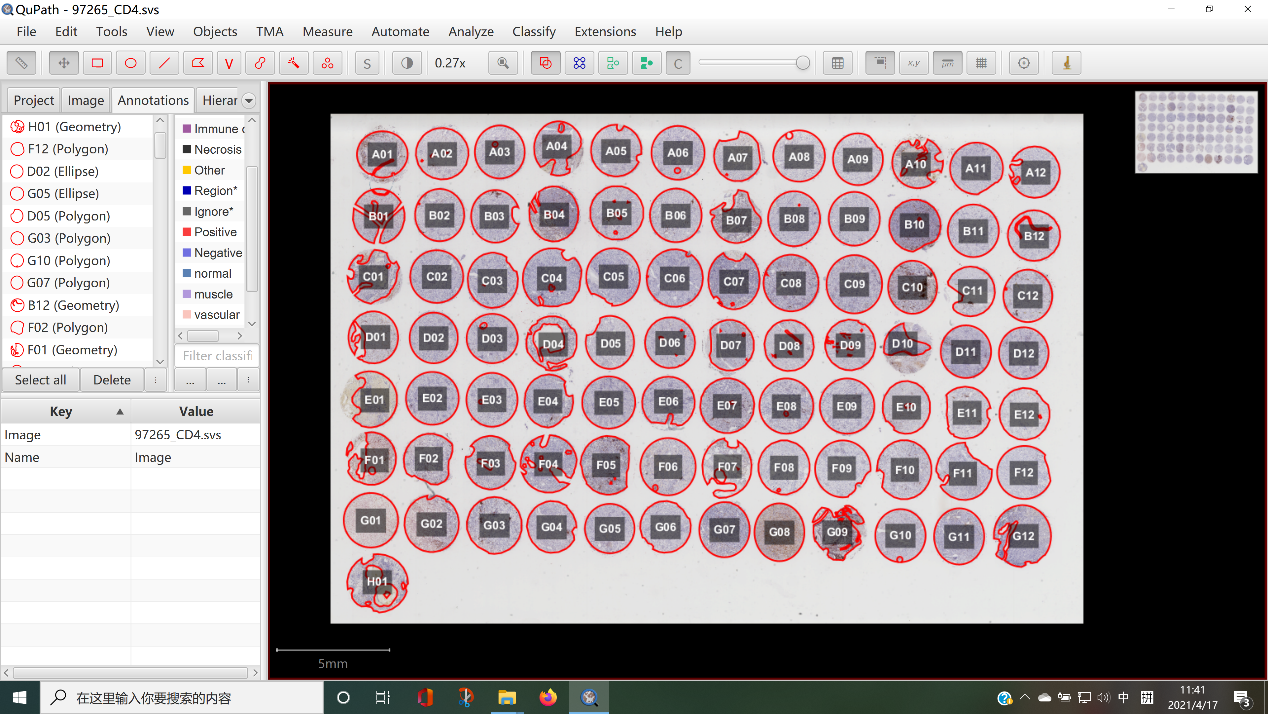


Step3: Detecting all cells within each annotation.


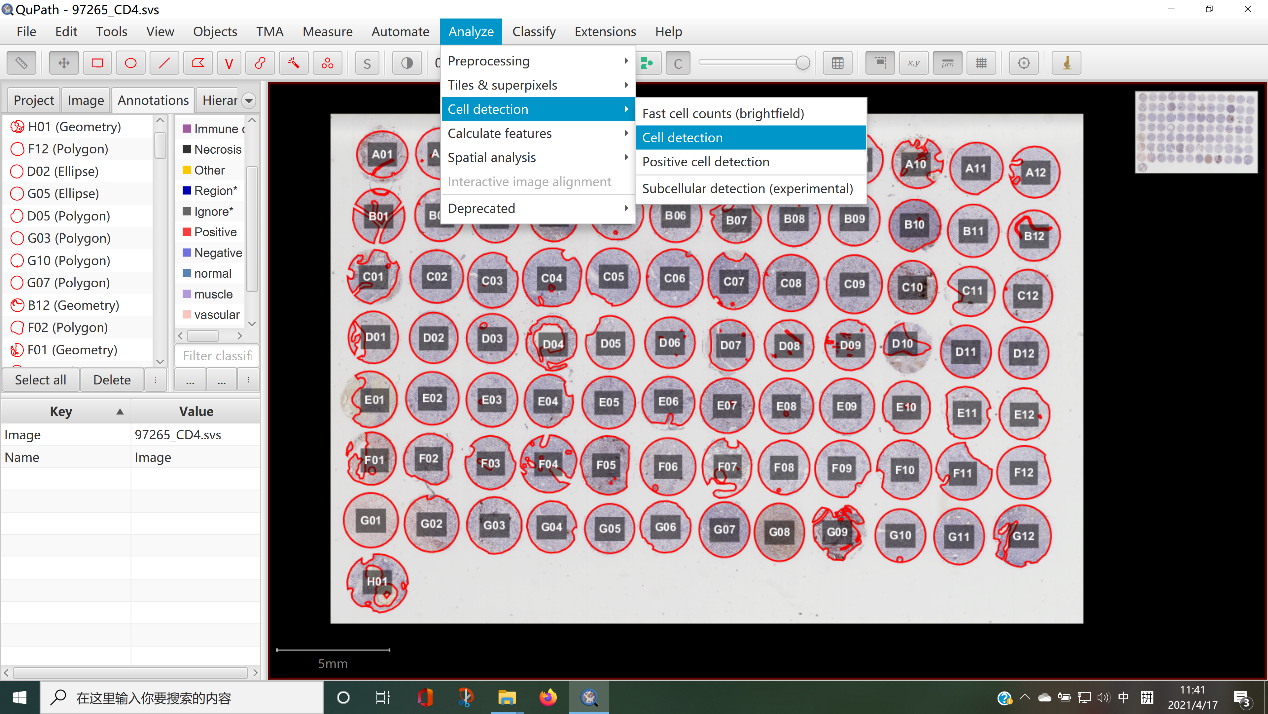


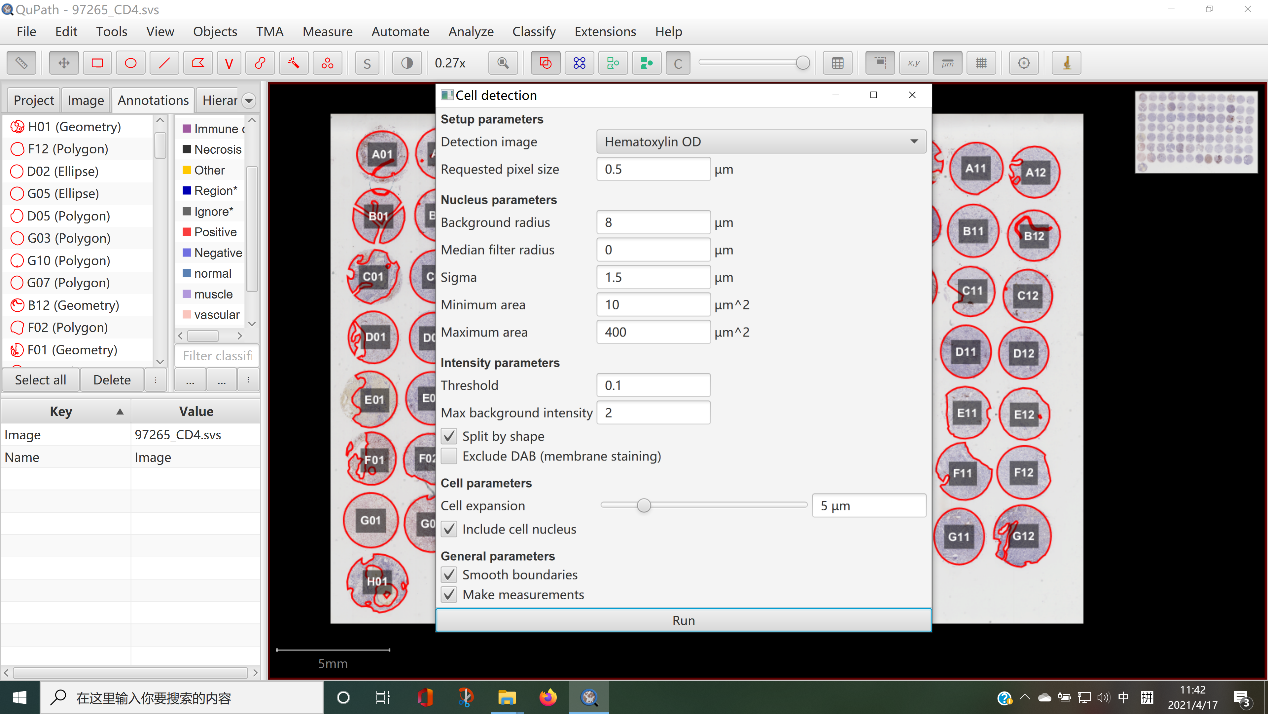


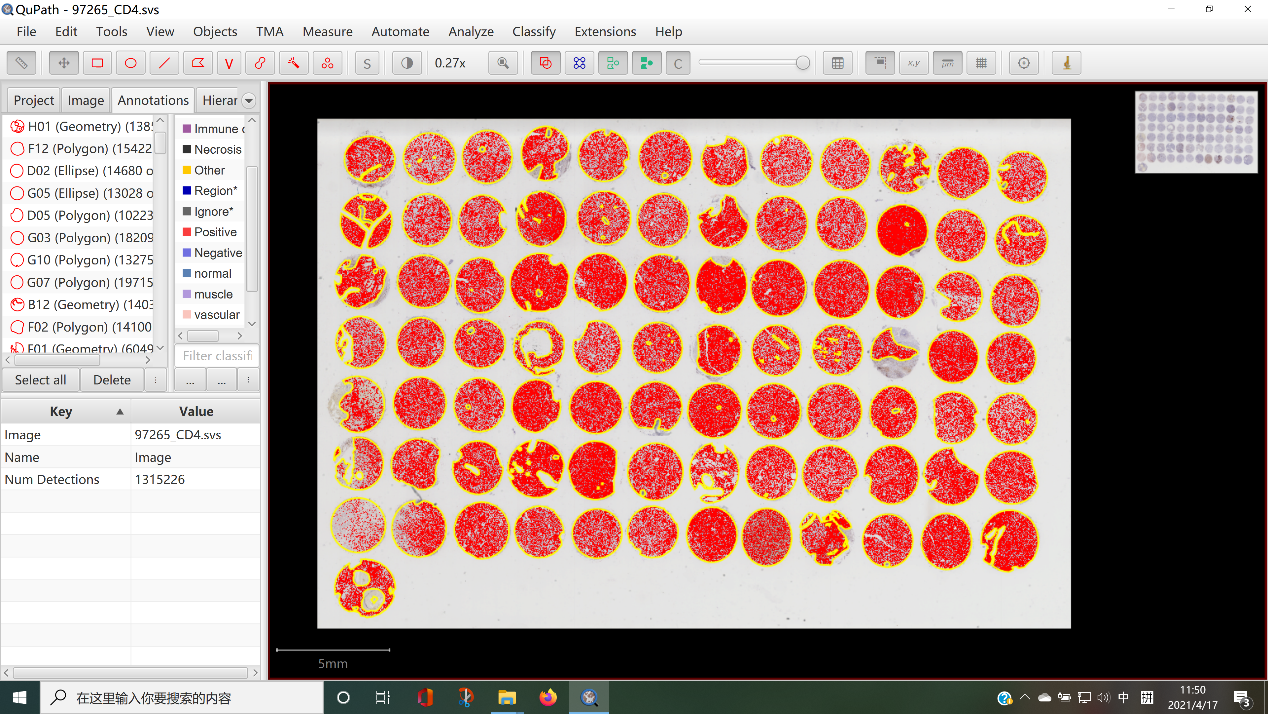


Step4: Estimate the immunohistochemical scores with positive cells ratio, Allred score and H-score.


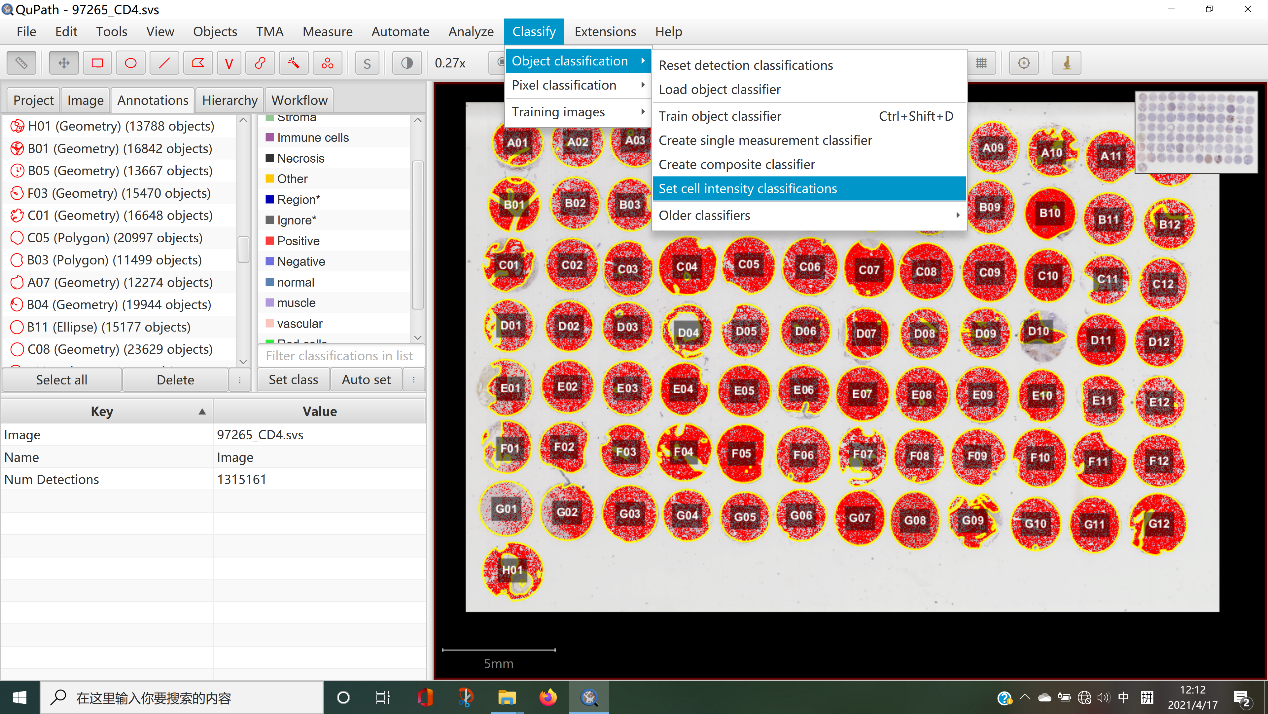


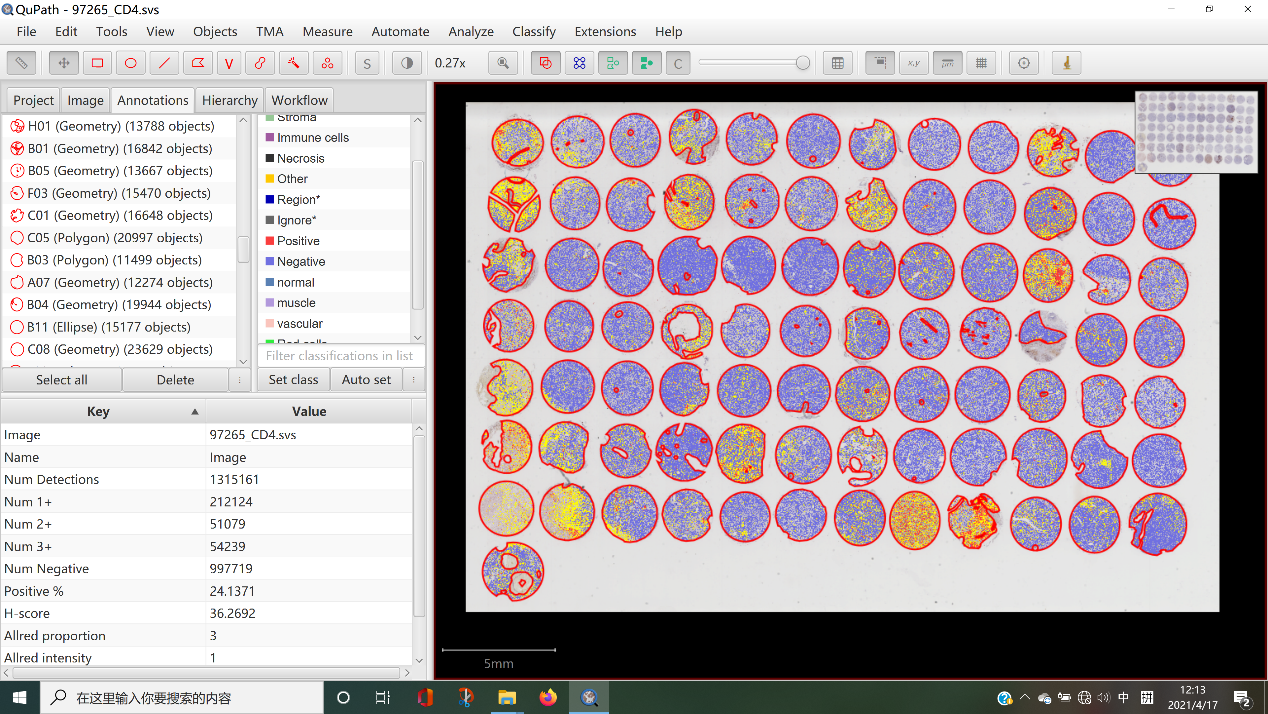


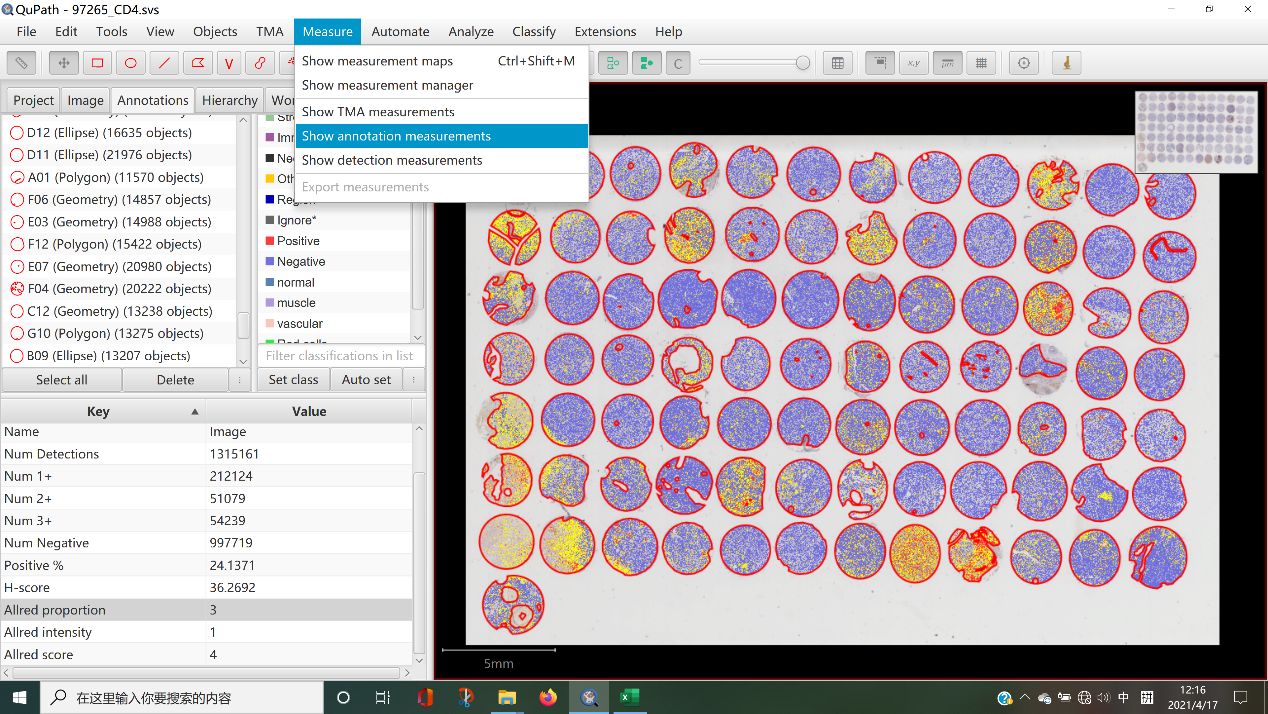


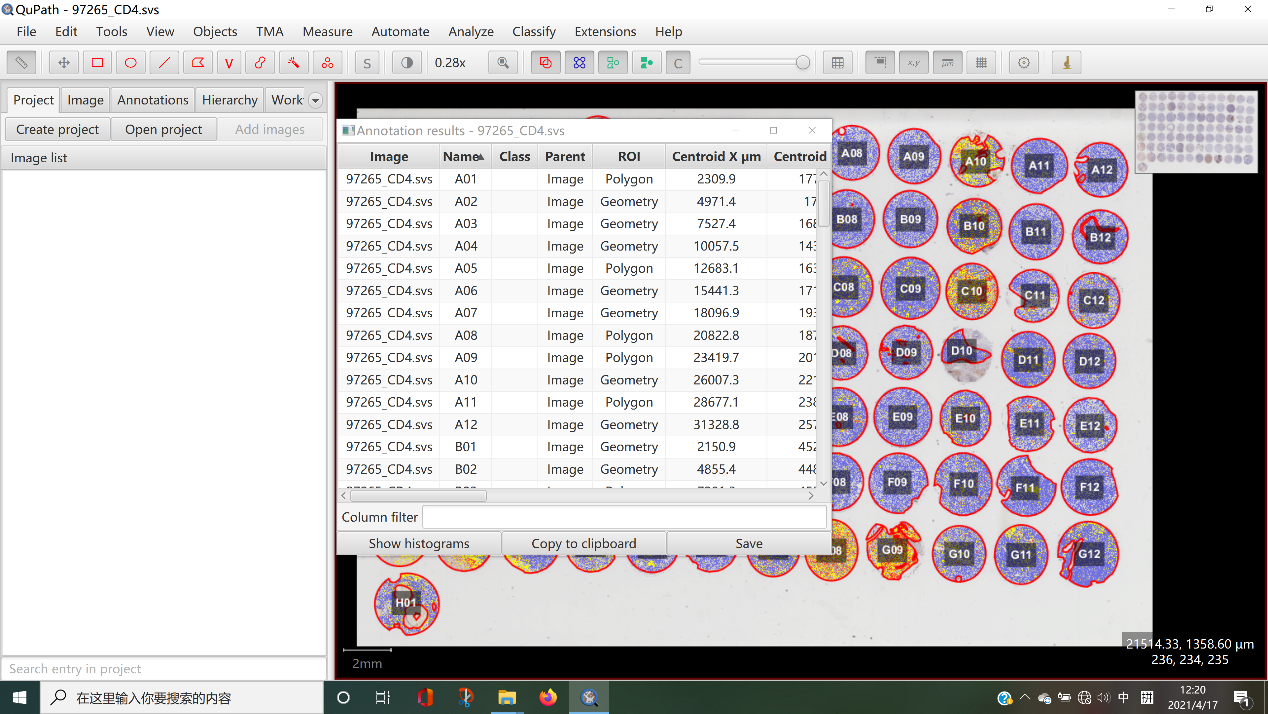

Supplement: Supplementary Materials — Differentially expressed lncRNA, miRNAs, and mRNAs were put in Supplementary Material 1. Gene ID of lncRNAs, miRNAs and mRNAs in the ceRNA network were displayed in Supplementary Material 2. For a comprehensive digital IHC image analysis with Qupath, please refer to the protocol in Supplementary Material 3. [file 4084461.f1.zip › supplementary material 3.docx]
